# Designed Ankyrin Repeat Proteins as a tool box for analyzing p63

**DOI:** 10.1038/s41418-022-01030-y

**Published:** 2022-06-18

**Authors:** Alexander Strubel, Philipp Münick, Apirat Chaikuad, Birgit Dreier, Jonas Schaefer, Jakob Gebel, Christian Osterburg, Marcel Tuppi, Birgit Schäfer, Stefan Knapp, Andreas Plückthun, Volker Dötsch

**Affiliations:** 1 Institute of Biophysical Chemistry and Center for Biomolecular Magnetic Resonance, Goethe University, 60438 Frankfurt, Germany; 2 Institute of Pharmaceutical Chemistry, Goethe University, 60438 Frankfurt, Germany; 3 Structural Genomics Consortium, Goethe University, 60438 Frankfurt, Germany; 4 Department of Biochemistry, University of Zurich, 8057 Zurich, Switzerland

**Keywords:** Biophysical chemistry, X-ray crystallography

## Abstract

The function of the p53 transcription factor family is dependent on several folded domains. In addition to a DNA-binding domain, members of this family contain an oligomerization domain. p63 and p73 also contain a C-terminal Sterile α-motif domain. Inhibition of most transcription factors is difficult as most of them lack deep pockets that can be targeted by small organic molecules. Genetic knock-out procedures are powerful in identifying the overall function of a protein, but they do not easily allow one to investigate roles of individual domains. Here we describe the characterization of Designed Ankyrin Repeat Proteins (DARPins) that were selected as tight binders against all folded domains of p63. We determine binding affinities as well as specificities within the p53 protein family and show that DARPins can be used as intracellular inhibitors for the modulation of transcriptional activity. By selectively inhibiting DNA binding of the ΔNp63α isoform that competes with p53 for the same promoter sites, we show that p53 can be reactivated. We further show that inhibiting the DNA binding activity stabilizes p63, thus providing evidence for a transcriptionally regulated negative feedback loop. Furthermore, the ability of DARPins to bind to the DNA-binding domain and the Sterile α-motif domain within the dimeric-only and DNA-binding incompetent conformation of TAp63α suggests a high structural plasticity within this special conformation. In addition, the developed DARPins can also be used to specifically detect p63 in cell culture and in primary tissue and thus constitute a very versatile research tool for studying the function of p63.

## Introduction

Investigation of the function of a protein can be achieved either at the genetic level or at the biochemical level. While knock-out strategies provide the most robust information, they represent permanent modifications. If temporal flexibility of the induction of the knock-out or only a partial knockdown is of interest, the use of siRNA or proximity-induced degradation are preferred alternatives. The disadvantage of all these methods is that multi-domain proteins are completely removed and the functions of the individual domains cannot be investigated separately. The most flexible way for the knock-out of the function of a particular domain is the use of small-molecule inhibitors which have proven to be very important and flexible tools for the characterization of entire protein classes, for example kinases or bromodomain-containing proteins [1–3]. One prerequisite for the development of a selective inhibitor is the presence of druggable binding pockets into which small organic molecules can bind. Consequently, enzymes are excellent targets. In contrast, protein-protein interactions often involve large and relatively flat interfaces. Thus, developing small organic molecules that target such protein-protein interaction sites has remained difficult.

An alternative is the design of proteins that bind to a certain interface and block its interaction with its natural partner. While antibodies are well established and are often used to detect or to inhibit extracellular proteins, their large size, multi-valency and the dependence on disulfide bonds reduce their usefulness for intracellular applications in living cells. These shortcomings have been addressed by developing single-chain Fv antibodies (scFvs) that were used to create “Intrabodies” without disulfide bonds [4–6]. As a useful alternative Designed Ankyrin Repeat Proteins (DARPins) have been developed [7–9]. These designed proteins use the Ankyrin repeat fold consisting of two antiparallel helices, connected by a loop to the next unit. Three to five of these repeat units can be combined into a single binding protein. It carries at the N- and the C-terminus special capping repeat modules to shield the hydrophobic core. The loops connecting the helices as well as the helix surface can be randomized to create a contiguous concave binding surface. In-vitro selection and evolution procedures such as ribosome display can be used to obtain high-affinity binders to a target [10, 11].

Due to this architecture of linearly arranged loops and parallel helices as binding interface, DARPins do not bind well to unstructured peptides but prefer to bind to structured domains. Because of their rigidity, they can also discriminate between different conformations of the same protein [12] or different isoforms [13]. The small size of DARPins (14–18 kDa), their monomeric nature, lack of disulfide bonds and high thermostability make them ideal tools to block specific protein-protein interaction surfaces. As many transcription factors contain, in addition to the DNA-binding domain, other folded protein-protein interaction modules, using DARPins to block the different domains can be used to investigate the distinct interactions and functions of transcription factors.

One important example is the p53 protein family that is involved in many cellular functions that include not only tumor suppression but also regulation of metabolism and developmental processes [14]. For the initial development of DARPins as tools to study the p53 protein family we have focused on p63 [15]. This transcription factor is involved in the proliferation and differentiation of epithelial tissues [16, 17] as well as in genetic quality control in female germ cells [18]. It is a multi-domain protein with an N-terminal transactivation domain (TAD) [19], a DNA-binding domain (DBD) [20], a tetramerization domain (TD) [21–23], a Sterile α-motif (SAM) domain [24] and a C-terminal transactivation inhibitory domain (TID) [25, 26] (Supplementary Fig. 1A). p63 is expressed in two main isoforms that either contain the N-terminal transactivation domain (TAp63α) or lack this domain (ΔNp63α) [27]. In epithelial tissues ΔNp63α is mainly expressed in the basal compartment and acts as a chromatin-organizing factor by binding to several thousand binding sites on the DNA [28–30]. This function potentially requires interaction with many other proteins including other transcription factors and chromatin-remodeling complexes. In contrast to this role in epithelial tissues, TAp63α in resting oocytes does not interact with the DNA in a sequence-specific manner but adopts a closed, inactive and only dimeric conformation [31]. Detection of DNA damage, however, results in the induction of an open and active tetrameric state through consecutive phosphorylation by the two kinases Chk2 [32] and CK1 [33, 34]. This active state initiates an apoptotic program resulting in the elimination of the damaged oocyte [35, 36]. In addition to TAp63α, two more isoforms, GTAp63α and TA*p63α, exist and are characterized by a 37 and 39 amino acid N-terminal extension, respectively. This extension stabilizes the closed dimeric state further [37]. GTAp63α seems to be involved in quality control in male germ cells [38], the role of TA*p63α, however, is not yet well understood.

To further investigate the involvement of p63 and the role of its domains in these different processes we have developed DARPins as tight binders. Here we describe the characterization of these DARPins as a tool box for intracellular inhibition, immunofluorescence applications, and pulldown assays. All DARPins were selected against and all experiments were performed with human p63 unless stated otherwise.

## Results

We created DARPins against the p63 DBD, the TD and the SAM domain using established in-vitro protein evolution schemes based on ribosome display [10, 11]. The general selection strategy was similar to that described previously [8]. For the initial screen, we used a Homogenous Time-Resolved Fluorescence (HTRF) screen that provided several binding DARPins per domain. For their further characterization we used biophysical methods (Isothermal titration calorimetry (ITC), fluorescence anisotropy, and pulldown assays) as well as structure determination to identify the interaction interface. In addition, we examined the specificity of binding against the corresponding p53 and p73 domains.

### DNA-binding domain

**Fig. 1:**
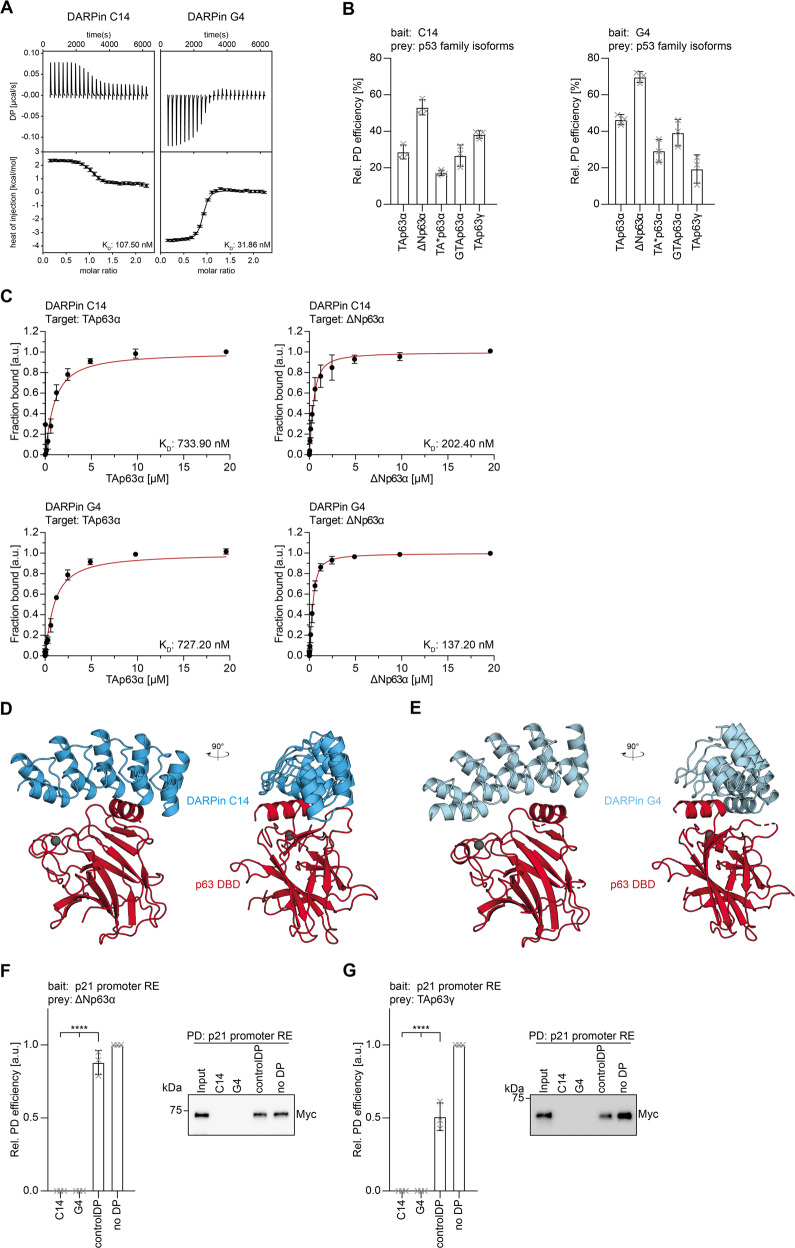
Characterization of DBD-specific DARPins. **A** ITC measurements of the DARPins C14 and G4 interacting with the DBD of p63. In each plot the top diagram displays the raw measurements and the bottom diagram shows the integrated heat per titration step. *K*_D_ values are given for each DARPin. **B** Pulldown experiments with different in-vitro translated p63 isoforms and immobilized DARPins, showing that both DARPins bind all isoforms. The pulldown efficiency relative to the whole protein expression is displayed on the *y*-axis (*n* = 3). **C** Fluorescence anisotropy measurements of Alexa 488 labeled DARPins with purified full-length TAp63α and ΔNp63α isoforms confirming that both DARPins bind all dimeric and tetrameric isoforms. The *K*_D_ values are provided for each experiment. **D** Crystal structure of the DARPin C14 in complex with the p63 DBD shown in two different orientations rotated by 90°. **E** Crystal structure of the DARPin G4 in complex with the p63 DBD shown in two different orientations rotated by 90°. **F**, **G** DNA-pulldown experiments with ΔNp63α (**F**), TAp63γ (**G**), and an immobilized DNA oligomer containing the 20 bp binding site of the human p21 promoter. Pre-incubation of the p63 isoforms with DARPin C14 or DARPin G4 inhibits interaction with the DNA oligomer while a control DARPin does not prevent binding. Corresponding western blot results are shown on the right. The relative pulldown efficiency normalized to no DARPin is shown on the *y* axis (*n* = 3). The bar diagram shows the mean values and error bars show the corresponding SD of three biological replicates. Statistical significance was assessed by ordinary one-way ANOVA.

In the initial screen with the isolated p63 DBD two DARPins were selected for further characterization. Using ITC titration experiments we measured a dissociation constant *K*_D_ of 108 nM for DARPin C14 (Fig. 1A; Supplementary Table 1) and 32 nM for DARPin G4 (Fig. 1A; Supplementary Table 1) while no interaction with a control DARPin could be detected (Supplementary Fig. 1B). Both selected DARPins also showed a significant shift in elution volume when combined with the purified p63 DBD on size exclusion chromatography indicating the formation of stable complexes (Supplementary Fig. 1C).

We further investigated if the DARPins can interact with the p63 DBD in the context of full-length proteins and not only with the isolated domain. This question is important since some of the p63 isoforms form closed, compact, and only dimeric states (TAp63α, TA*p63α, GTAp63α) [31, 37], while others adopt open and tetrameric conformations (TAp63γ, ΔNp63α) [31] (Supplementary Fig. 1A). Dimeric isoforms do bind DNA only weakly, suggesting that the DBD is at least partially buried within the closed dimeric conformation which in turn could prevent interaction with the DARPins [31, 37]. To test if the DBD is accessible we generated by in-vitro translation the proteins TAp63α, TA*p63α, GTAp63α, TAp63γ, and ΔNp63α and incubated the lysate with DARPins immobilized on streptavidin magnetic beads. The results of these pulldown assays demonstrated that both DARPins C14 and G4 bind to all p63 isoforms (Fig. 1B, Supplementary Fig. 1D). To investigate whether DARPin binding affinities are affected by steric hindrance in the full-length protein we performed fluorescence anisotropy measurements. DARPins were labeled outside the binding site with an Alexa 488 fluorophore maleimide by introducing a C-terminal cysteine into the DARPins (Supplementary Fig. 5A, B). Fluorophore-labeled DARPins were titrated with bacterially expressed and purified p63 isoforms (Supplementary Fig. 1F) and the data confirmed that both DARPins C14 and G4 bind to the DBD in both closed dimeric isoforms (TAp63α) as well as to open and tetrameric ones (ΔNp63α) with only weak influence on binding affinities (Fig. 1C; Supplementary Fig. 1E and Supplementary Table 2).

To identify the exact interaction interfaces of the DARPins to the p63 DBD we crystallized both DARPins in complex with the p63 DBD. The structures revealed that both DARPins bind to the DNA-binding interface and interact with the helix H3, the preceding loop and the C-terminal part of β-sheet S10 as well as residues in loop L3 (Fig. 1D, E; Supplementary Fig. 1G, H). All these sites are involved in and crucial for DNA binding [20]. The loop L1 that in p53 provides an additional contact via K120 [39] (conserved in p63), which however is not essential for DNA binding [40], interacts with the non-randomized scaffold of the DARPins, resulting in steric hindrance but no specific interaction. In the second DARPin-DBD complex within the asymmetric units present in both crystals, this L1 loop is disordered. All contacts between both proteins are summarized in Fig. 1D, E and Supplementary Fig. 1G-J. These data suggest that interaction with the DARPins competes with binding to DNA (Supplementary Fig. 1K, L) and that the DARPins act as a competitive inhibitor for DNA binding. To test this prediction, we performed pulldown assays with a biotinylated DNA oligomer comprising the human p21 promoter sequence, coupled to streptavidin magnetic beads. ΔNp63α and TAp63γ expressed in H1299 cells were efficiently pulled down in this experiment. Pre-incubation with either DARPin C14 or G4 resulted in complete inhibition of binding to the DNA oligomer while a control DARPin showed no significant effect (Fig. 1F, G; Supplementary Fig. 7).

In combination the crystal structures and the interaction studies with full-length TAp63α, TA*p63α, and GTAp63α show that the DNA-binding interface of these inhibited and only dimeric conformations is in principle accessible. This is in agreement with earlier studies in which we had detected a strongly reduced but still detectable affinity for DNA of TAp63α as compared to tetrameric ΔNp63α [31]. These studies thus show that DARPins can also be used for probing the accessibility of interfaces in larger complexes.

### Tetramerization domain

The tetramerization domain of p63 differs from the oligomerization domain of p53 by the presence of a second α-helix per monomer that reaches across the tetramerization interface and thus stabilizes the tetramer [21, 22] (Supplementary Fig. 2A). Within the dimeric conformations of TAp63α, TA*p63α, and GTAp63α only half of the tetramerization domain consisting of the β-strand and the first α-helix of two monomers is present. The second helix cannot adapt the orientation known from tetrameric states and might even be unfolded. Tetramerization is prevented by blocking the tetramerization interface by an antiparallel β-sheet [41] formed by sequences near the N-terminal transactivation (TA) domain and the C-terminal transactivation inhibitory (TI) domain [25].

**Fig. 2:**
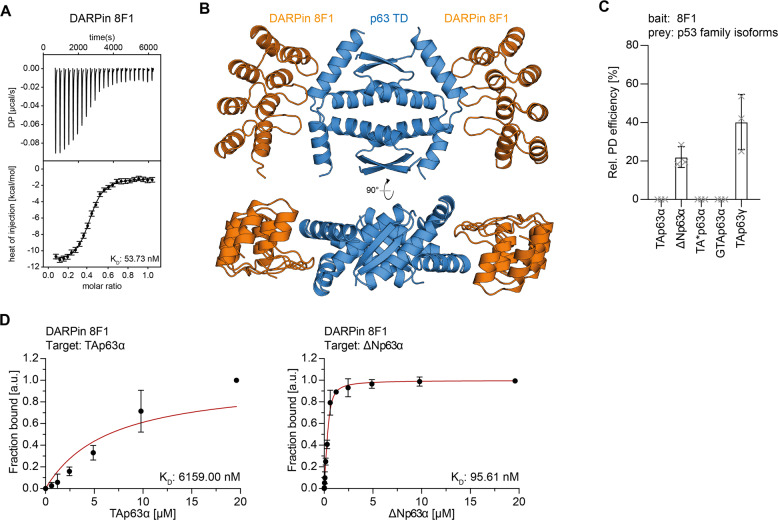
Characterization of the TD-specific DARPin. **A** ITC measurements of the DARPin 8F1 interacting with the TD of p63. The top diagram displays the raw measurement and the bottom diagram shows the integrated heat per titration step. The K_D_ value is given for the interaction. **B** Crystal structure of the DARPin 8F1 in complex with the p63 TD showing a 2:1 stoichiometry in two different orientations that are rotated by 90^°^. **C** Pulldown experiments with different in-vitro translated p63 isoforms and immobilized DARPin 8F1. The results show that only open and tetrameric isoforms that exhibit a complete TD bind to the DARPin. The pulldown efficiency relative to the whole protein expression is displayed on the *y*-axis (*n* = 3). **D** Fluorescence anisotropy measurements of Alexa 488 labeled DARPin 8F1 with purified full-length TAp63α and ΔNp63α isoforms. Only the tetrameric ΔNp63α isoform binds strongly while only a negligible interaction is detected for dimeric TAp63α.

DARPins were generated against the complete tetramerization domain including the second α-helix. After initial selection screens, the identified DARPin 8F1 was further characterized by ITC measurements showing a K_D_ value of 54 nM (Fig. 2A, Supplementary Fig. 2B, and Supplementary Table 1). Size exclusion chromatography experiments of DARPin 8F1 and the p63 TD showed a shift in elution volume indicating a stable complex consisting of both proteins (Supplementary Fig. 2C).

The crystal structure of DARPin 8F1 in complex with the tetramerization domain revealed a 2:1 DARPin:TD ratio, consistent with the stoichiometry observed in ITC, with each DARPin binding to the hinge region connecting helix 1 and helix 2 of two monomers (Fig. 2B, Supplementary Fig. 2D–F). Because these two hinge regions are part of the tetramerization interface this structure predicted that only tetrameric but not dimeric conformations of p63 should interact with this DARPin. To investigate this hypothesis, we performed pulldown experiments with DARPin 8F1 immobilized on streptavidin magnetic beads and with in-vitro translated TAp63α, TA*p63α, GTAp63α, TAp63γ, and ΔNp63α. As expected, the DARPin did not bind to the stable dimeric isoforms TAp63α, TA*p63α, and GTAp63α but did bind to the tetrameric isoforms TAp63γ and ΔNp63α (Fig. 2C, Supplementary Fig. 1D). In addition, we measured fluorescence anisotropy experiments with the fluorescently labeled DARPin (Supplementary Fig. 5A, B) and purified full-length p63 isoforms (Supplementary Fig. 1F). Consistently with the pulldown and ITC data, DARPin 8F1 does not bind dimeric p63 isoforms but does bind tetrameric ΔNp63α (Fig. 2D; Supplementary Fig. 1E and Supplementary Table 2). Intriguingly, our findings thus demonstrated that DARPin 8F1 represents a conformation-specific binder for tetrameric p63 isoforms.

### Sterile α-motif (SAM) domain

**Fig. 3:**
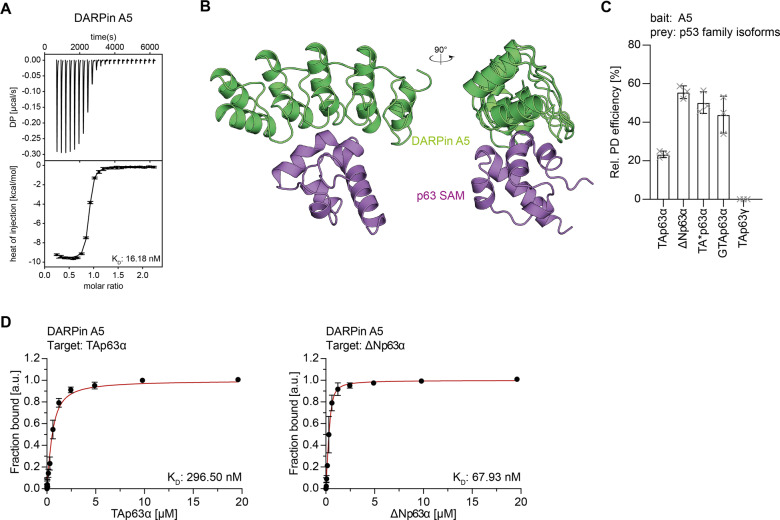
Characterization of the SAM domain-specific DARPin. **A** ITC measurements of the DARPin A5 interacting with the SAM domain of p63. The top diagram displays the raw measurement and the bottom diagram shows the integrated heat per titration step. The *K*_D_ value is given for the interaction. **B** Crystal structure of DARPin A5 in complex with the p63 SAM domain shown in two different orientations rotated by 90°. **C** Pulldown experiments with different in-vitro translated p63 isoforms and immobilized DARPin A5. Only p63 isoforms containing a SAM domain are bound by DARPin A5. The pulldown efficiency relative to the whole protein expression is displayed on the *y*-axis (*n* = 3). **D** Fluorescence anisotropy measurements of Alexa 488 labeled DARPin A5 with purified full-length TAp63α and ΔNp63α isoforms. Both dimeric (TAp63α) and tetrameric (ΔNp63α) bind to the SAM domain.

The third and last folded domain of p63 is the Sterile α-Motif (SAM) domain that is present in p63 and p73 but is missing in p53 [24] (Supplementary Fig. 1A). The initial screens resulted in the identification of p63 SAM domain-specific DARPin A5 that was subject for further characterization. ITC measurements with the isolated p63 SAM domain showed a K_D_ of 16 nM (Fig. 3A, Supplementary Fig. 3A, and Supplementary Table 1) and the formation of a stable complex was confirmed by a shift in elution volume on size exclusion chromatography (Supplementary Fig. 3B). Pulldown assays with in-vitro translated full-length p63 isoforms demonstrated binding to both dimeric (TAp63α, TA*p63α, GTAp63α) and open tetrameric isoforms (ΔNp63α) but showed no binding to TAp63γ, an isoform that lacks the SAM domain (Fig. 3C; Supplementary Fig. 1D). These results were confirmed by fluorescence anisotropy measurements with fluorescently labeled DARPin (Supplementary Figs. 1F and 5A, B), showing interaction with the SAM domain both in closed and dimeric (TAp63α) as well as open and tetrameric (ΔNp63α) isoforms (Fig. 3D; Supplementary Fig. 1E and Supplementary Table 2).

Structure determination of a complex of DARPin A5 with the p63 SAM domain (Fig. 3B) revealed that the DARPin bound to helices 1 and 2 mainly by hydrophobic interactions (Supplementary Fig. 3C–E). Currently, the exact function of the SAM domain is not known [26], therefore we cannot speculate if the interaction with the DARPin results in an inhibition of the SAM domain’s function.

### Selectivity

**Fig. 4:**
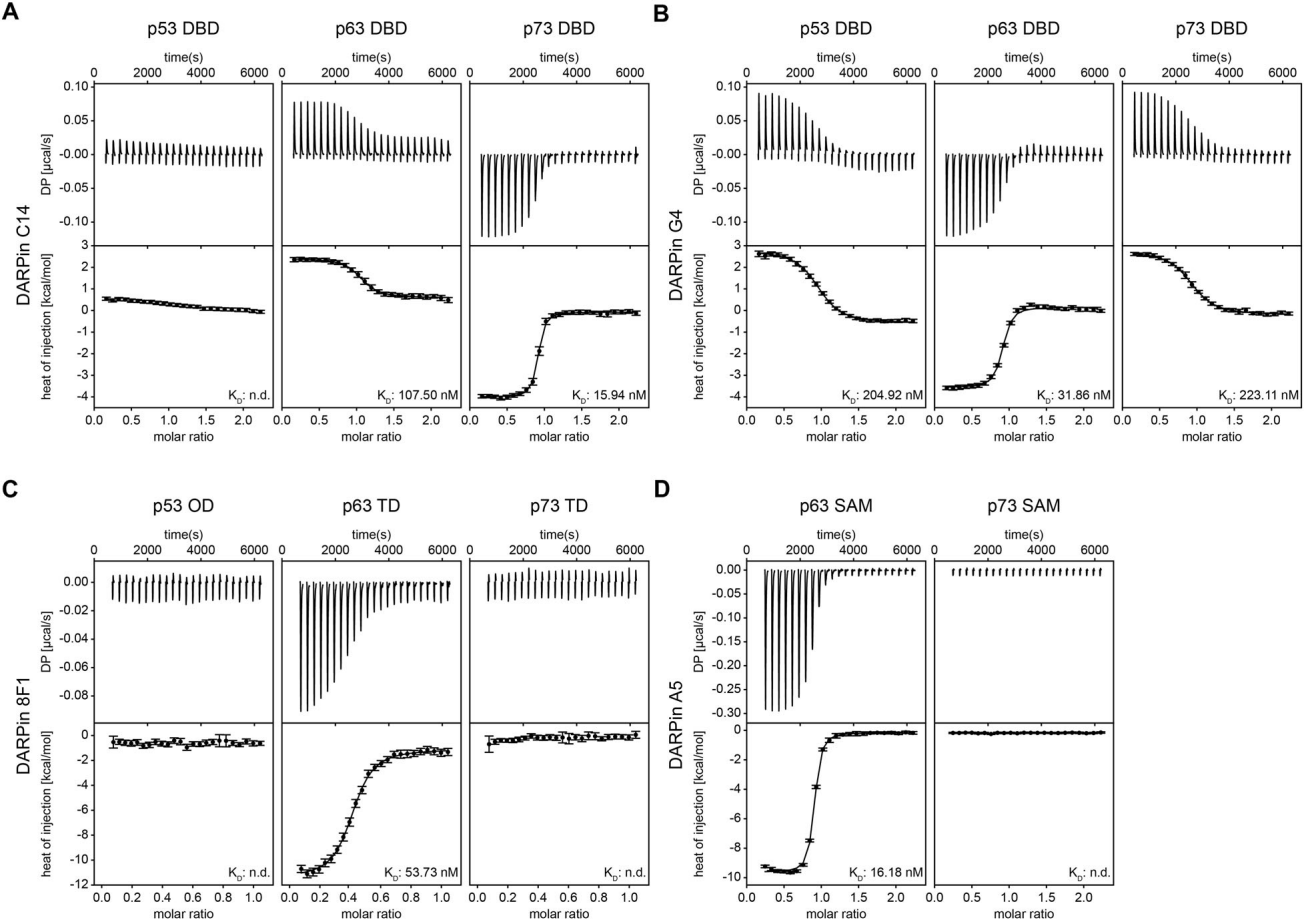
Investigation of the specificity of the DARPins for binding to p63. ITC measurements are shown for the two DBD-specific DARPins C14 (**A**) and G4 (**B**) as well as for the TD-specific DARPin 8F1 (**C**) and the SAM-domain-specific DARPin A5 (**D**). Experiments were performed with the p63 domains and the corresponding domains of p53 and p73. Since p53 does not have a SAM domain, this family member is not included in (**D**). The top diagrams display the raw measurements and the bottom diagrams show the integrated heat per titration step. The *K*_D_ values are given for each interaction. The data show that the DARPin C14 binds the DBD of both p63 and p73 while the DARPin G4 binds all three family members. The DARPins 8F1 and A5 bind specifically only to the p63 domains.

An important question for the development of inhibitors is their selectivity. During the initial HTRF screen DARPins selected against the p63 domains were cross-screened for binding to the corresponding domains of p73 and p53. We further investigated the specificity of the selected DARPins by ITC experiments. These experiments showed that for the DBD a selective p63 binding DARPin was not identified since DARPin C14 bound to the p73 DBD with 16 nM affinity as well. Virtually no interaction was detected with the p53 DBD (Fig. 4A; Supplementary Table 1), however. DARPin G4 bound to the DBD of all three p53 family members (205 nM to p53 DBD, 223 nM to p73 DBD) as this DARPin was not counter-selected against any of the other DBDs (Fig. 4B; Supplementary Table 1). For the TD and the SAM domain, the high selectivity of the p63-only binding DARPins 8F1 and A5 was confirmed (Fig. 4C, D; Supplementary Table 1). The ITC results were further supported by pulldown assays with in vitro translated full-length p63 isoforms and in addition full-length p53 and TAp73α. These data showed high selectivity of the p63 TD and p63 SAM domain-specific DARPins 8F1 and A5, as well as DARPin C14 for p63 and p73 isoforms. DARPin G4 bound to all p53 family members (Supplementary Fig. 4D, E). A control DARPin showed no binding to any of the targets (Supplementary Fig. 4A–D, F; Supplementary Fig. 7).

### Staining of p63 isoforms in cells

**Fig. 5:**
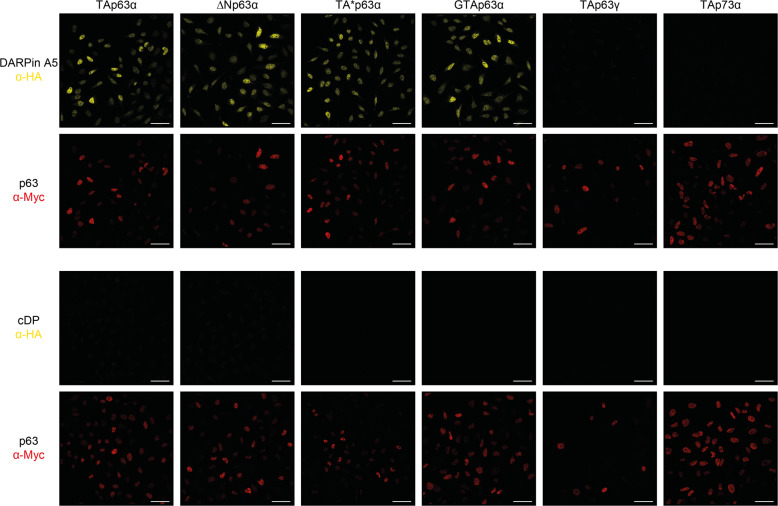
Detection of different p63 isoforms in stably expressing HeLa cells. Cells expressing the indicated p63 isoforms or as a control TAp73α, were fixed with formaldehyde and incubated with HA-tagged DARPin A5, followed by the goat anti-HA antibody (a190138a - Bethyl) and the secondary antibody Alexa Fluor 568 anti-goat (A11057—Life Technologies). The same cells were also stained with the mouse anti-myc antibody 4A6 (Millipore) and the secondary antibody Alexa Fluor 647 anti-mouse (A31571—Life Technologies) as all p63 isoforms and TAp73α are labeled with an N-terminal myc-tag. All SAM domain-containing p63 isoforms show strong staining while TAp63γ which lacks a SAM domain does not show any signal above background. Cells expressing TAp73α, which has a SAM domain, do not show staining either demonstrating the specificity of the DARPin A5 for the p63 SAM domain. A control DARPin does not show any signal above background. Scale bar, 50 µm.

Detection of proteins by immunohistochemistry is a widespread method for diagnostic and experimental examinations. To develop a DARPin-based detection technique we used HeLa cells stably expressing either TAp63α, TA*p63α, GTAp63α, ΔNp63α, TAp63γ, or TAp73α as a control. Cells were fixed with formaldehyde and incubated with a solution containing the SAM domain-specific DARPin A5, modified with a HA-tag at its N-terminus. After washing, cells were incubated with an anti-HA antibody followed by a fluorophore-labeled secondary antibody. As control, cells were also incubated with a myc-tag-specific antibody followed by the secondary antibody as all p63 isoforms and TAp73α were modified with a myc-tag at their N-terminus. Fluorescence microscopy detected strong DARPin-created signals in cells expressing TAp63α, TA*p63α, GTAp63α, and ΔNp63α but not in TAp63γ and TAp73α expressing cells, demonstrating the high specificity for p63 SAM domain-containing proteins of DARPin A5. A control DARPin, also modified with a HA-tag, did not show any signal above background (Fig. 5).

Similar experiments with the DBD and TD DARPins were, unfortunately, not successful. Formaldehyde used for fixing the cells is known to chemically react with amino acid side chains and their modification most likely modifies the binding epitope of these two DARPins.

### DARPins as transcriptional inhibitors

**Fig. 6:**
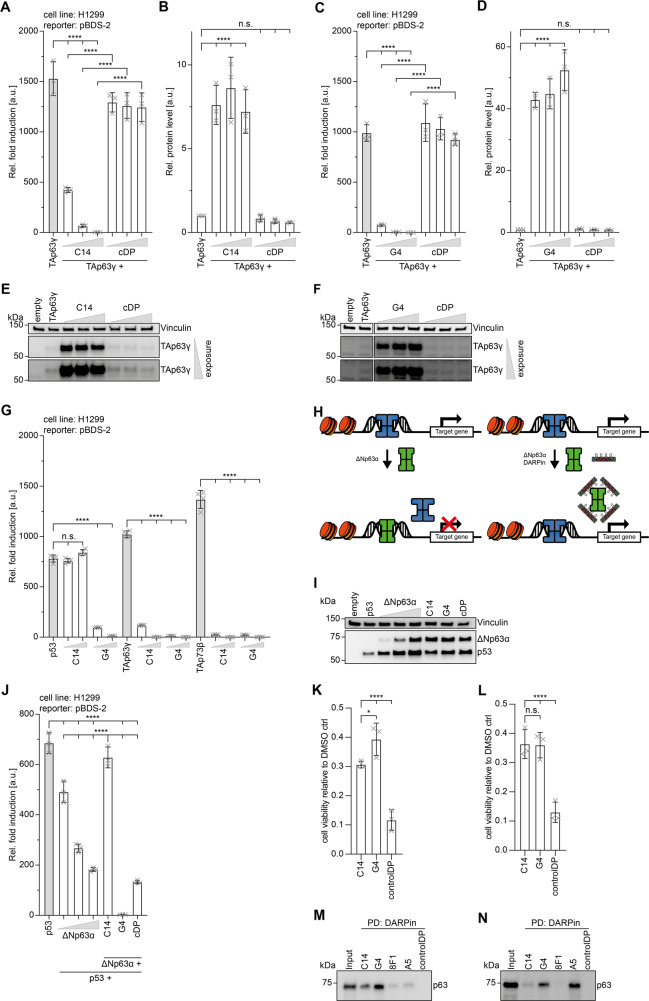
DARPins C14 and G4 as inhibitors and pulldown experiments of p63 from primary tissue. **A** Transactivation assays in H1299 cells with a luciferase expression construct under the control of a pBDS-2 promoter show strong transcriptional activity of TAp63γ. Co-expression of DARPin C14 inhibits transcriptional activity in a concentration-dependent manner. Co-expression of a control DARPin shows no effect. **B** Quantitative analysis of the protein level of TAp63γ of the experiment shown in (**A**). The corresponding western blots are displayed in (**E**). **C** The same experiments as in (**A**) with the DARPin G4. **D** Quantitative analysis of the protein level of TAp63γ of the experiment shown in (**C**). The corresponding western blots are displayed in (**F**). For all experiments in **A**-**D**, the bar diagrams show the mean values and error bars the corresponding SD of three biological replicates. Statistical significance was assessed by ordinary one-way ANOVA. **E**, **F** show the western blot results of the experiments in (**A**) and (**C**) detecting the level of myc-tagged TAp63γ with the anti-myc antibody 4A6 (Millipore). The level of vinculin serves as a loading control. Results with two different exposure times are shown. Co-expression of inhibitory DARPins result in a strong increase of the cellular level of TAp63γ, presumably by inhibiting its degradation. A control DARPin has no effect. The quantitative analysis is provided in (**B**) and (**D**). **G** Transactivation assay with p53, TAp63γ and TAp73β in H1299 cells. All three family members show high transcriptional activity. The transcriptional activity of p53 is inhibited by co-expression of DARPin G4, while DARPin C14 has no effect. The transcriptional activity of p63 and p73 is inhibited by both DARPins. The bar diagram shows the mean values and error bars show the corresponding SD of four technical replicates. Statistical significance was assessed by ordinary one-way ANOVA. **H** Schematic representation of the inhibitory effect of ΔNp63α on the transcriptional activity of p53. High concentrations of ΔNp63α (green) as they occur in squamous cell carcinoma displace p53 (blue) from the promoter sequences (left). Selective binding of DARPins to the DBD of p63 enables binding of p53 to these promoter sequences and thus transcription of the affected genes (right). **I** The transcriptional activity of p53 can be inhibited by ΔNp63α in a concentration-dependent manner as suggested in (**H**). Western blot analysis of the cellular levels of ΔNp63α and p53 of the data quantitatively analyzed in (**J**). Vinculin is used as a loading control. **J** Quantitative analysis of the reduction of the transcriptional activity of p53 with increasing concentration of ΔNp63α. The last three lanes show results of co-expressing ΔNp63α at its highest concentration. Expressing DARPin C14 in addition re-established the transcriptional activity of p53 while expression of DARPin G4 which binds to both the p53 and p63 DBD inhibits transcriptional activity completely. Co-expression of a control DARPin does not re-establish p53-based activity beyond the level seen without any DARPin but in the presence of the highest ΔNp63α concentration used. The bar diagram shows the mean values and error bars show the corresponding SD of three biological replicates. Statistical significance was assessed by ordinary one-way ANOVA. **K** Endpoint analysis of viable cells 89 h after 0.5 µM DOX treatment. The number of viable cells expressing control DARPin and TAp63α with a T2A self-cleaving peptide as a linker is significantly reduced compared to cells expressing DARPin C14 or G4. The bar diagram shows the mean values and error bars show the corresponding SD of three biological replicates. Statistical significance was assessed by ordinary one-way ANOVA. **L** Endpoint analysis of viable cells 89 h after 0.7 µM DOX treatment. The number of viable cells expressing control DARPin and TAp63α with a T2A self-cleaving peptide as a linker is significantly reduced compared to cells expressing C14 or G4. The bar diagram shows the mean values and error bars show the corresponding SD of three biological replicates. Statistical significance was assessed by ordinary one-way ANOVA. **M** Pulldown experiment of ΔNp63α from mouse skin extract. DBD-specific DARPins C14 and G4, TD-specific DARPin 8F1 and the SAM-domain-specific DARPin A5 were bound to streptavidin magnetic beads and incubated with the extract. The levels of ΔNp63α pulled down by the DARPins were measured by western blot analysis using an anti-p63 antibody (ab124762 - Abcam). All four DARPins show signals, with DARPin G4 binding strongest. The experiment was done in biological triplicates. One representative replicate is shown. **N** Same experiment as in (**M**) with extract produced from mouse ovaries. The pulldown experiments show that the oocyte-specific isoform TAp63α gets efficiently pulled down by DARPins G4 and in particular DARPin A5 but not DARPin 8F1, proving DARPin 8F1 as a conformation-specific binder of tetrameric p63 isoforms. The experiment was done in biological triplicates. One representative replicate is shown.

One of the main advantages of DARPins over antibodies is that they can be employed as selective inhibitors in intracellular assays. To test if the DARPins selected for the DBD can act as inhibitors in living cells we used a luciferase-based transactivation assay. Transient transfection of the transcriptionally most active isoform, TAp63γ, into H1299 cells showed a strong transactivation on the pBDS-2 promoter. Increasing amounts of expressed DARPin C14 or G4 in these cells reduced the transactivation in a concentration-dependent manner while expression of a control DARPin had no effect (Fig. 6A, C). Interestingly, the western blot analysis of the cellular level of TAp63γ showed that co-expression of both inhibitory DARPins stabilizes the protein (Fig. 6B, D–F; Supplementary Fig. 7). This is consistent with earlier observations that transcriptionally active p63 isoforms are efficiently degraded while isoforms with a low transcriptional activity (ΔNp63α, dimeric TAp63α) accumulate in cells [42]. Likewise, mutations in the DNA-binding domain, as they occur in patients suffering from Ectrodactyly-ectodermal dysplasia-cleft (EEC) syndrome [43] inhibit DNA binding and result in accumulation of mutant p63 [44]. These observations that suggest the existence of a negative feedback loop similar to the Mdm2-p53 system were so far based on mutational analysis. Our DARPin inhibitor study is the first confirmation of this effect with wild-type protein and shows the power of functional inhibitors.

Our study also showed that DARPin C14 bound selectively to the p63 and p73 DBDs while the DARPin G4 bound the DBD of all three family members in a concentration-dependent manner, whereas a control DARPin showed no significant effect (Fig. 6G; Supplementary Fig. 4G). These findings were confirmed by DNA-pulldown assays, performed as described before (Fig. 1F, G). Pulldown with the immobilized p21 promoter oligomer revealed inhibition of binding of p53 by DARPin G4 whereas DARPin C14 and a control DARPin showed no effect (Supplementary Fig. 6A). p73 was significantly blocked from DNA binding by both DARPins but not by a control DARPin (Supplementary Fig. 6B).

### Reactivation of p53 transcriptional activity

The tumor suppressor function of p73 and in particular that of p53 have been very well documented [14]. In contrast, a role of p63 in tumor suppression remains much debated. Nonetheless, its function as an oncogene is well established for the ΔNp63α isoform. This isoform is highly expressed in squamous cell carcinoma (SSC) cells and its overexpression is believed to be the driving force for tumorigenesis [45]. Furthermore, SSC cells seem to be dependent on ΔNp63α overexpression. Mechanistically, ΔNp63α competes with p73 and p53 and when overexpressed inhibits their binding to promoter regions of genes important for tumor suppression (Fig. 6H) [46]. We wanted to test if inhibition of DNA binding of ΔNp63α through DBD-binding DARPins may reactivate p53 by using a luciferase-based transactivation assay. Co-expression of p53 and increasing amounts of ΔNp63α showed the expected and previously reported inhibition of the transcriptional activity of p53 in H1299 cells [45–47] (Fig. 6H–J; Supplementary Fig. 7). Co-expression of DARPin C14 reactivated the transcriptional activity of p53. However, co-expression with DARPin G4 did not result in an increase of the transcriptional activity as DARPin G4 also binds and inhibits the p53 DBD. A control DARPin had no effect on the transcriptional activity of p53 (Fig. 6I, J).

### Cell survival assays

The inhibitory effect of the DARPins on the transcriptional activity of p63 predicts that they could suppress p53 family dependent induction of apoptosis. To test the effect of the DARPins C14 and G4 we created cell lines that stably express a fusion construct of the DARPin and TAp63α. Both proteins are linked via a T2A (thosea asigna virus 2A) peptide that on translation becomes ‘cleaved’ by ribosome skipping [48], releasing the DARPin (Supplementary Fig. 6C). This procedure ensured a ~1:1 expression level of DARPin and TAp63α. Cells were treated with 0.5 µM or 0.7 µM doxorubicin to activate TAp63α from the inactive dimeric to the active tetrameric state which induces apoptosis via expression of Puma and Noxa [35, 36]. Cell viability was measured for 89 h using an ATP-based assay. The data showed that DARPins C14 and G4 protected cells compared to the control DARPin, suggesting that both DARPins suppressed the induction of apoptosis by inhibiting the transcriptional activity of activated TAp63α (Fig. 6K, L; Supplementary Fig. 6D, E).

### Detection of p63 in primary tissues

So far, our experiments have either been performed with purified proteins or in cell culture. We also investigated if our DARPins can be used to detect p63 in primary tissue. As human and mouse p63 have a very high sequence identity [15], we used mice as the source for primary tissue. The ΔNp63α isoform is highly expressed in the basal layers of epithelial tissues [15]. We created homogenous extracts from mouse skin and incubated these extracts with our DARPins, biotinylated at their C-terminal Avi-tag and immobilized on streptavidin magnetic beads. All our selected DARPins were effective in pulldown experiments showing a pulldown signal for p63. The DBD DARPins C14, and in particular G4, were most effective in these pulldown experiments while the TD (8F1) and SAM domain (A5) DARPins showed only weak pulldown signals (Fig. 6M; Supplementary Fig. 7).

The other tissue with high p63 content are the ovaries in which the TAp63α isoform is highly expressed in oocytes [18]. We prepared mouse ovary extracts and used our DARPins for pulldown experiments similar to the skin experiments. DARPin G4 and the SAM domain-specific DARPin A5 showed a strong pulldown signal for p63 while the tetramer-specific DARPin 8F1 showed no pulldown signal at all, consistent with the fact that oocytes only contain dimeric TAp63α [31] (Fig. 6N; Supplementary Fig. 7).

## Discussion

DARPins are a very versatile class of molecules that can be used for detection as well as intracellular inhibitors for functional studies. Due to their specificity for folded domains, they can selectively recognize different conformations and oligomeric states. Selective DARPins have for example been raised against the extracellular signal-regulated kinase (ERK) both in its nonphosphorylated (inactive) or doubly phosphorylated (active, p-ERK) form [49] and the c-Jun N-terminal kinase-1 and 2 (JNK1 and JNK2) [13]. The JNK DARPins can be used as intracellular inhibitors of the kinase activity in either an isoform-specific way or as pan-JNK inhibitors.

Here we could show that the DARPin raised against the TD selectively recognizes only tetrameric but not dimeric p63 isoforms and can thus be used as a conformation-specific binder. Furthermore, the ability of the DBD-specific DARPins and the SAM domain-specific DARPin to bind their respective domains also in the closed and dimeric conformations of TAp63α, TA*p63α and GTAp63α shows that within these complexes these domains are most likely not rigidly connected to the other domains but show high flexibility. This is in agreement with earlier studies in which we could replace the DBD with GFP in TAp63α without disrupting the closed dimeric state. Similarly, the SAM domain can be completely removed without conformational change of TAp63α [41].

The binding affinities of the DARPins that we characterized in this study are in the nanomolar range. The fluorescence anisotropy experiments showed some variations of affinities compared to those of the ITC experiments using isolated domains. This can be explained by the aggregation propensity of the α-C-terminus of some isoforms, making it hard to measure the real concentration of the protein applied in the experiment [50]. In particular, the ΔNp63α isoform that contains an open and accessible TI domain shows a higher aggregation tendency and can only be expressed with an N-terminal MBP fusion. While the measured affinity is high enough for many studies, in particular when the amount of DARPins to be used is not limited it might not be sufficient for other applications. One of the big advantages of the DARPin system, however, is that it can easily be adjusted and modified. For example, two or more DARPins can be covalently linked as a single-chain construct. Since the p53 protein family forms multimers combining for example two or more DARPins binding the DNA-binding domain can increase the binding affinity by avidity effects. Alternatively, DARPins targeting different domains can be linked as well. A further alternative to single-chain constructs is to combine two (or also more) DARPins via fusion with leucine zipper domains which creates a different geometry compared to the “beads on a string” mode [51–53].

## Methods

### Selection and screening of DARPin binders specific for p63 domains

To generate DARPin binders for human p63 protein and subdomains, *Escherichia coli* expression plasmids of *E. coli* biotin ligase BirA and full-length target proteins containing an Avi-tag were co-transformed in BL21(DE3) Rosetta cells (SGC Frankfurt) for protein production and in-vivo biotinylation. Cells were grown in 2xYT medium supplemented with 100 µM ZnCl_2_ and 10 µM biotin. Proteins were expressed and purified as described. The biotinylated target protein was immobilized alternating on either MyOne T1 streptavidin-coated beads (Thermo Fisher Scientific) or Sera-Mag neutravidin-coated beads (Cytiva), depending on the selection round. Ribosome display selections were performed essentially as described [10], using a semi-automatic KingFisher Flex MTP96 well platform.

The fully synthetic library consists of N3C-DARPins with three randomized internal repeats, containing a mixture of non-randomized and randomized N-terminal and C-terminal capping repeats [7, 11, 54]. Selections were performed over four rounds with decreasing concentrations of biotinylated target protein for the first three cycles, an off-rate selection using non-biotinylated target protein in the third cycle followed by a fourth round with less stringent conditions [10, 55].

The final enriched pool of the DARPin-encoding cDNA was cloned as fusions with a N-terminal MRGSH_8_- and C-terminal FLAG tag into a derivative of pQE30 (QIAGEN) containing a *lacI*^q^ gene via unique *Bam*HI and *Hind*III restriction sites under the control of a T5lac promoter. After transformation of *E*. *coli*, 380 single DARPin clones were expressed in 96-well format and lysed by addition of a concentrated Tris-HCl-based HT-lysis buffer containing n-octyl β-D-thioglucopyranoside (OTG), lysozyme and universal nuclease (Pierce). These bacterial crude extracts of single DARPin clones were centrifuged and supernatants subjected to Homogeneous Time-Resolved Fluorescence (HTRF)-based screening to identify potential binders. Binding of the FLAG-tagged DARPins to streptavidin-immobilized biotinylated target protein was measured using FRET (donor: Streptavidin-Tb cryptate (610SATLB, Cisbio), acceptor: mAb anti-FLAG M2-d2 (61FG2DLB, Cisbio)). Experiments were performed at room temperature in white 384-well Optiplate plates (PerkinElmer) using the Taglite assay buffer (Cisbio) at a final volume of 20 μl per well. FRET signals were recorded after an incubation time of 30 min using a Varioskan LUX Multimode Microplate (Thermo Scientific) with the following settings: Delay time: 60 μs, integration time: 200 μs, measurement time: 1000 ms, dynamic range: automatic. HTRF ratios were obtained by dividing the acceptor signal (665 nm) by the donor signal (620 nm) and multiplying this value by 10,000 to derive the 665/620 ratio. The same HTRF assay conditions were used to analyze cross-reactivity to other isoforms or domains. If DARPins showed high affinity to the corresponding domains of p53 and/or p73 they were no further characterized. The exception are the DBD-binding DARPins as no DARPin only selective for p63 could be identified. In addition, the DARPin G4 was not analyzed for cross-reactivity at this stage at all.

From the identified binders, usually 32 were sequenced and single clones identified. For the selection of p63 SAM specific DARPins, those that were unique and single clones were expressed on a small scale and purified using a 96-well IMAC column (HisPur^TM^ Cobalt plates, Thermo Scientific). DARPins after IMAC purification were analyzed for potential oligomerization tendency at a concentration of 10 µM on a Superdex 200 increase 5/150 GL column (GE Healthcare) using a LC1200 HPLC system (Agilent) with PBS containing 400 mM NaCl as the running buffer. Absorbance at 280 nm was recorded.

### Cell culture

The non-small cell lung cancer cell line H1299 was cultured in RPMI medium 1640 (Gibco), containing 10% FBS (Capricorn Scientific), 100 U/ml penicillin (Gibco) and 100 µg/ml streptomycin (Gibco) at 37 °C and 5% CO_2_. H1299 cell line was obtained from ATCC. T-REx-HeLa cell line was cultured in DMEM medium (Gibco), containing 10% FBS (Capricorn Scientific), 4 µg/ml blasticidin (Gibco), 333 µg/ml Zeocin (Gibco), 100 U/ml penicillin (Gibco), 100 µg/ml streptomycin (Gibco) and 1 mM pyruvate (Gibco) at 37 °C and 5% CO_2_. The T-REx-HeLa cell line was a gift from Christian Behrends (Munich Cluster for Systems Neurology (SyNergy), Ludwig-Maximilians-University (LMU), Munich, Germany).

All cell lines used in this study were routinely tested for mycoplasma contaminations.

For recombinant protein expression, H1299 cells in medium without antibiotics were transfected using Lipofectamine 2000 transfection reagent (Thermo Fisher Scientific) according to the manufacturer’s recommendation. 6 h after transfection the medium was exchanged to standard H1299 culturing medium.

### Generation of HeLa cells stably expressing p63 isoforms

For generation of stable inducible expressing p53 family isoforms HeLa cell lines the Flp-In T-REx system (Thermo Fisher Scientific) for homologous recombination of the target genes was used. After two weeks of culturing the T-REx-HeLa cells were transfected in a six-well plate using the Lipofectamine 2000 transfection reagent (Thermo Fisher Scientific) with pcDNA5/FRT/TO (Thermo Fisher Scientific) containing p53 family isoforms, respectively, as well as pOG44 (Thermo Fisher Scientific) containing the Flp recombinase according to the manufacturer’s recommendation. After transfection DMEM medium containing 10% tetracycline-free FBS (Bio Cell) was used. The next day after transfection cells were reseeded in 15 cm dishes. One day after cell transfer the medium was exchanged to Selection Medium with DMEM containing 10% tetracycline-free FBS, 4 µg/ml blasticidin, 200 µg/ml hygromycin (Thermo Fisher Scientific), 100 U/ml penicillin, 100 µg/ml Streptomycin and 1 mM pyruvate. Cells were cultured until a non–transfected control showed no viable cells (about 10–14 days). Twelve single colonies of each cell line were isolated, cultured and inducible expression of desired protein was tested using western blot. Protein expression was induced by adding 1 µg/mL tetracycline (Thermo Fischer Scientific) to selection medium for 24 h. For further experiments three individual clones of each p53 family isoform were chosen.

### Molecular cloning

For recombinant expression in *E. coli*, PCR-generated inserts were introduced in pET-15b-His_10_-TEV (N-terminal His_10_-tag followed by a tobacco etch virus (TEV) protease cleavage side), pET-15b-His_10_-TEV-Avi (N-terminal His_10_-tag followed by a TEV protease cleavage side and Avi-tag), pET-15b-His_10_-TEV-HA (N-terminal His_10_-tag followed by a TEV protease cleavage side and HA-tag), pET-15b-His_10_-TEV-GGC (N-terminal His_10_-tag followed by a TEV protease cleavage side and C-terminal GGC-tag), pET-15b-GFP-His_8_-TEV (N-terminal GFP followed by a His_8_-tag and a TEV protease cleavage side), pGEX-6P-2-His_8_-TEV (N-terminal GST-tag followed by His_8_-tag and TEV protease cleavage side) or pMal-His_10_-TEV (N-terminal MBP-Tag followed by His_10_-tag and TEV protease cleavage side) by subcloning using *Bam*HI and *Xho*I restriction sides. DARPin sequence information was provided by Andreas Plückthun. For transient expression in mammalian cells, PCR-generated inserts were introduced in pcDNA3.1(+)-Myc by subcloning using *Bam*HI and *Xho*I. An overview with detailed protein and domain definitions corresponding to construct design is shown in Supplementary Fig. 1A.

### Protein expression and purification

*DARPins*. *E. coli* BL21(DE3) Rosetta cells (SGC Frankfurt) were transformed with individual expression plasmids for protein production. Cells were grown in 2xYT medium to an OD of 0.8. Protein expression was induced with 0.6 mM IPTG for 16 h at 16 °C. Cells were harvested by centrifugation, resuspended in IMAC A buffer (50 mM Tris, pH 8, 400 mM NaCl) supplemented with RNAse (Sigma), DNAse (Sigma), lysozyme (Sigma), self-made protease inhibitors and lysed by sonification. The lysate was cleared by centrifugation at 4 °C, supernatant was supplemented with 30 mM imidazole and applied onto a pre-equilibrated immobilized metal affinity chromatography (IMAC) column (HiTrap IMAC Sepharose FF, Cytiva) following an IMAC purification protocol. Bound protein was washed with IMAC A buffer supplemented with 50 mM imidazole and eluted by a step gradient with IMAC A buffer supplemented with 300 mM imidazole. The eluted protein was then simultaneously dialyzed to IMAC A buffer and digested with TEV protease (self-made). TEV protease and undigested protein were separated by a reverse IMAC step. For DARPin constructs with a C-terminal cysteine for labeling all IMAC buffers were supplemented with 40 mM β-mercaptoethanol. The purified proteins were further polished and buffer exchanged by size exclusion chromatography (SEC) with SEC buffer (50 mM Tris, pH 8, 150 mM NaCl, 0.5 mM TCEP) using a Superdex 75 10/300 column (Cytiva). Central monomeric and monodisperse peak fractions were collected, concentrated to the desired concentration (Amicon Ultra Centrifugal Filters, Millipore) and flash-frozen in liquid nitrogen prior to storage at −80 °C until use. Purity and molecular size of purified proteins were monitored by SDS-PAGE and LC-ESI-TOF-mass spectrometry.

*p63 SAM, p53 family domains and p63 isoforms*. Individual *E. coli* expression plasmids were transformed and expressed as described before. Except for plasmids containing a DNA-binding domain (DBD), the medium was supplemented with 100 µM ZnCl_2_. Proteins were purified as described before using IMAC buffers supplemented with 20 mM β-mercaptoethanol. For proteins harboring a DBD IMAC buffers supplemented with 20 mM β-mercaptoethanol and 10 µM ZnCl_2_ were used. The purified proteins were polished, concentrated and stored as described before. p63 isoform constructs were applied onto a Hiload Superose 6 16/600 (Cytiva) column and TAp63α onto a Hiload Superdex 200 16/600 (Cytiva) column. Purity and molecular size of purified proteins was monitored by SDS-PAGE and LC-ESI-TOF-mass spectrometry.

### DARPin biotinylation

For in-vitro enzymatic biotinylation *E. coli* biotin ligase BirA was subcloned into a pET-15b-GFP-His_8_-TEV *E. coli* expression vector. GFP-BirA was expressed and purified as described before, except for a TEV cleavage and reverse IMAC step.

DARPins containing an Avi-tag were enzymatically biotinylated in-vitro by mixing with GFP-BirA in a 1:50 molecular ratio in SEC buffer supplemented with 10 mM ATP, 10 mM MgCl_2_, 0.5 mM biotin and incubating for 16 h at 16 °C. For separation the reaction mix was applied onto a Superdex 75 10/300 column (Cytiva). DARPin fractions were pooled and analyzed by LC-ESI-TOF-mass spectrometry. Only DARPins showing one hundred percent labeling efficacy were used for experiments.

### Gel electrophoresis and western blotting

Purified proteins were mixed with SDS loading buffer (250 mM Tris, pH 8.0, 7.5% (w/v) SDS, 25 % (w/v) glycerol, 12.5 % (v/v) β-mercaptoethanol, 0.025 % (w/v) bromophenol blue), denatured at 95 °C and separated on manually prepared discontinuous 4–16 % Tris-Glycine gels. The gels were subsequently stained using Quick Coomassie Stain (NeoBiotech) according to the manufacturer’s recommendation.

Samples for immunoblotting were either mixed with SDS loading buffer or NuPAGE LDS buffer (Thermo Fisher Scientific) supplemented with DTT, denatured at 95 °C and applied on 4–15 % Mini-PROTEAN TGX Stain-Free Precast Protein gels (Bio-Rad). The gels were transferred using the Trans-Blot Turbo Transfer System (Bio-Rad) according to the manufacturer’s recommendation. Membranes were blocked for 1 h in TBS-T with milk (TBS, 0.05 % (v/v) Tween-20, 5 % skim milk powder, Sigma-Aldrich), followed by incubation with primary antibody in TBS-T containing milk and overnight shaking at 4 °C. Membranes were washed three times with TBS-T and secondary antibody in TBS-T-containing milk was incubated under shaking for 1 h at room temperature. Thereafter, membranes were washed three times with TBS-T and analyzed by adding Amersham ECL Prime WB Detection Reagent (Cytiva). Quantification of western blot signals was performed using ImageJ (Version 1.51). The following antibodies and dilutions were used for immunoblotting detection: anti-myc (1:2000, clone 4A6, Millipore), anti-p63 (1:2000, ab124762, Abcam), anti-vinculin (1:2000, clone 7F9, Santa Cruz), goat anti-mouse HRP (1:5000, A9917, Sigma-Aldrich) and goat anti-rabbit HRP (1:2000, Jackson ImmunoResearch Europe Ltd).

### DARPin fluorescence labeling

DARPins containing C-terminal cysteines were fluorescently labeled using Alexa Fluor 488 C5 Maleimide (Invitrogen). Thiol groups were reduced with freshly degassed reduction buffer (PBS with 5 mM DTT, 1 mM EDTA, pH 7.4) for 4 h at 37 °C. DTT was removed from protein using HiTrap desalting columns (Cytiva) with freshly degassed PBS on an Äkta purifier system (Cytiva) and subsequently incubated with a 2:1 molar excess of maleimide dye for 1 h at 25 °C in the dark. The reaction was stopped by adding excess DTT. For separation of free fluorophores and different labeling derivatives, the reaction mix was reduced in salt to below 50 mM by dilution with IEX A (50 mM Tris, pH 8.3) and applied onto a Q HP anion exchange chromatography column (Cytiva). Labeling derivates were eluted and separated by applying an increasing gradient of IEX B (50 mM Tris, pH 8.3, 1000 mM NaCl) for 30 min from 0–60 % IEX B, 3 ml/min. The chromatography was monitored at 280 nm and 495 nm, central fluorescent peak fractions were pooled and subsequently buffer was exchanged using SEC in SEC buffer with a Superdex 75 10/300 column (Cytiva). Central peak fractions were pooled, concentrated to the desired concentration (Amicon Ultra Centrifugal Filters, Millipore) and analyzed by LC-ESI-TOF-mass spectrometry. DARPin samples containing only one single fluorophore were used for experiments. The concentration of labeled DARPin was determined as described by the Alexa Fluor 488 C5 Maleimide manufacturer’s instructions.

### Transactivation assay

One day after H1299 cells were seeded in 12-well plates the cells were transfected using Lipofectamine 2000 transfection reagent (Thermo Fisher Scientific) using the same plasmid amounts of pRL-CMV (Promega) and pBDS2 (Addgene plasmid #16515) in combination with varying pcDNA3.1(+) construct concentrations, dependent on the experimental application, according to the manufacturer’s instructions. 24 h after transfection, cells were washed in PBS (Gibco), detached and distributed in RPMI medium 1640 (Gibco) into white Nunc 96-well microplates (Thermo Fisher Scientific) in quadruplicates. The firefly and renilla luciferase activity was measured on a Spark plate reader (Tecan) using the Dual-Glo Luciferase reporter assay kit (Promega). The remaining sample was centrifuged for 5 min at 500 g, pelleted cells were mixed with SDS loading buffer and protein expression levels were analyzed by western blot. The experiment was repeated in three biological replicates and the ratio of firefly to renilla signal was normalized to empty vector control for each biological replicate. For statistical analysis the significance was analyzed by ordinary one-way ANOVA (n.s.: *P* > 0.05, *P ≤ 0.05, **P ≤ 0.01, ***P ≤ 0.001, ****P ≤ 0.0001) using Prism (Version 8.2.1, GraphPad).

### Reticulocyte lysate protein expression

Proteins were translated in-vitro using the TnT Coupled Reticulocyte Lysate System (Promega). Constructs in a pcDNA3.1(+) vector were diluted to 100 ng/µl with nuclease-free water (Promega) and mixed in a 1:4 volume ratio with rabbit reticulocyte lysate (RRL) and incubated for 90 min at 30 °C. The reaction was stopped by adding Benzonase (Millipore) for 30 min. The supernatant was cleared by centrifugation at 16,100 × *g* for 10 min at 4 °C and stored on ice.

### Pulldown assays

#### DARPin Pulldown assays

Target proteins were in-vitro translated. An excess of biotinylated DARPins were pre-incubated with pre-equilibrated magnetic Dynabeads MyOne Streptavidin T1 (Thermo Fisher Scientific) in Pulldown (PD) wash buffer (50 mM Tris, pH 8, 150 mM NaCl, 0.1 % (v/v) Tween-20) while rotating for 2 h at 4 °C. The magnetic beads were washed three times with PD wash buffer to remove unbound DARPins and were resuspended in PD wash buffer with the same volume as before to maintain magnetic bead concentrations. 10 µl DARPin loaded beads were mixed with 10 µl in-vitro translated protein, 1× complete protease inhibitor (Roche) and adjusted to a total volume of 1000 µl with PD wash buffer. The PD mix was incubated while rotating overnight at 4 °C. Pulldown samples were washed five times with 1000 µl PD wash buffer and eluted with LDS buffer by boiling at 70 °C for 10 min. Samples were analyzed by western blot as described before.

#### DARPin Pulldown assays of tissue samples

Skin tissue and ovaries were dissected from eight-day-old (P8) female CD-1 mice, purchased from Charles River Laboratories. Animal care and handling was performed according to the guidelines of the World Health Organization (Geneva, Switzerland). The Tierschutzbeauftragte of the Goethe University Frankfurt/Main approved the protocol for harvesting mouse ovaries and skin. Isolated ovaries were lysed with RIPA buffer (50 mM HEPES, pH 7.5, 150 mM NaCl, 1 mM MgCl_2_, 1 mM DTT, 1 % (v/v) NP40, 1 % (v/v) sodium deoxycholate, 1x complete protease inhibitor, Roche, 1× PhosSTOP, Roche) by five freeze and thaw cycles, grinding under liquid nitrogen using a reaction tube mini mortar (Bel-Art) followed by 1 h rotating at 4 °C. Isolated mouse skin tissue was lysed using a mortar (Sigma Aldrich) to grind tissue under liquid nitrogen. Ground skin powder was resuspended in RIPA buffer followed by 1 h rotating at 4 °C.

Ovary and skin lysates were cleared by a two-step centrifugation at 16,100 × *g* at 4 °C for 10 min each and the protein concentration of whole tissue lysates was assessed by a Pierce BCA Protein Assay Kit (Thermo Fisher Scientific). Benzonase (Millipore) was added to the lysate for 1 h. 0.25 mg of whole skin lysate or 0.12 mg whole ovary lysate, respectively, 20 µl pre-loaded DARPin magnetic beads (described before) and 1x complete protease inhibitor (Roche) were adjusted to a total volume of 1000 µl with PD wash buffer and were incubated while rotating overnight at 4 °C. Final samples were prepared and analyzed as described before.

#### DNA-pulldown assays

Target proteins were expressed in H1299 cells as described before. H1299 cells were harvested and lysed with lysis buffer (50 mM Tris, pH 8, 150 mM NaCl, 2 mM MgCl_2_, 0.5 mM TCEP, 20 mM CHAPS, 1× complete protease inhibitor, Roche) for 1 h on ice. Cell debris was removed by centrifugation at 16,100 × *g* for 10 min at 4 °C and whole lysate protein concentration was assessed by Pierce BCA Protein Assay Kit (Thermo Fisher Scientific). Dynabeads MyOne Streptavidin T1 (Thermo Fisher Scientific) were pre-incubated with a biotinylated 20-bp p63 response element as previously described for biotinylated DARPins. 0.1 mg whole H1299 lysate, 20 µl pre-loaded DNA magnetic beads and 1x complete protease inhibitor (Roche) were adjusted to 500 µl with PD wash buffer and incubated rotating 2 h at 4 °C. Final samples were prepared and analyzed as described before.

All pulldown experiments in this study were performed as biological triplicates. DARPin pulldowns were normalized to input and DNA pulldowns were normalized to sample without DARPin. For statistical analysis of all pulldowns the significance was analyzed by ordinary one-way ANOVA (n.s.: *P* > 0.05, *P ≤ 0.05, **P ≤ 0.01, ***P ≤ 0.001, ****P ≤ 0.0001) using Prism (Version 8.2.1, GraphPad).

### Immunofluorescence staining

Stable T-REx-HeLa cells were seeded on coverslips (Carl Roth) and expression of p53 family isoforms was induced as described before. Twenty-four hours after induction cells were washed twice with PBS and fixed with ROTI Histofix 4 % (Carl Roth) for 10 min at room temperature. Fixed cells were washed twice with PBS and permeabilized with PBS-T (PBS supplemented with 0.1 % Triton X-100, Carl Roth) for 5 min two times. Permeabilized cells were blocked with blocking buffer (PBS-T supplemented with 1 % BSA, Carl Roth) for 20 min at room temperature. Blocked cells were incubated with 100 nM HA-tagged DARPin and mouse anti-myc (1:500, clone 4A6, Millipore) antibody in blocking buffer overnight at 4 °C. Cells were washed five times with PBS-T and incubated with goat anti-HA (1:200, a190138a, Bethyl) antibody in blocking buffer for 2 h at room temperature. Cells were washed five times with PBS-T and incubated with Alexa Fluor 568 anti-goat antibody (1:200, A11057, Life Technologies) and Alexa Fluor 647 anti-mouse antibody (1:200, A31571, Life Technologies) in blocking buffer for 2 h at room temperature. Slides were washed five times with PBS-T and coverslips were mounted using Mowiol (Carl Roth) mounting medium which was supplemented with DAPI (Thermo Fisher Scientific). Detailed recipe of the mounting medium can be found at CSH protocols (http://cshprotocols.cshlp.org/content/2006/1/pdb.rec10255). The slides were dried several days before imaging with a LSM 780 confocal laser scanning microscope (Zeiss).

### Fluorescence anisotropy

Target proteins and DARPins were purified and labeled as described before. Fresh SEC of target proteins was performed freshly before use, and central peak fractions were used for the experiments. A constant concentration of 500 nM of Alexa 488 labeled DARPin was used for the measurements with a linear dilution series of target protein from 0–10 µM in a black 384-well plate (Corning) in anisotropy buffer (50 mM Tris, pH 8, 150 mM NaCl, 0.5 mM TCEP, 0.1 % Tween-20). Samples were incubated for 20 min at room temperature. Anisotropy data were assessed by using a Spark plate reader (Tecan) at 16 °C with a total assay volume of 10 µl. All measurements were carried out in triplicates. Data were analyzed and fitted using Prism (Version 8.2.1, GraphPad).

### Isothermal titration calorimetry

All titration experiments were performed using a MicroCal VP-ITC microcalorimeter (Malvern Instruments Ltd, UK). DARPins and target proteins were dialyzed against ITC buffer (50 mM HEPES, pH 7.4, 150 mM NaCl, 0.5 mM TCEP). Target proteins were titrated to constant concentrations of DARPin in 10 µl steps with total injections of 25 and 250 s spacing time at indicated temperatures. The reference power was set to 25 µCal/s and stirring speed to 307 rpm. NITPIC was used for unbiased baseline calculation and curve integration [56, 57]. Thermodynamic parameters and final binding affinity were generated by SEDPHAT [58] assuming an AB hetero-association model. The first data point was excluded from the analysis. Final publication grade figures were generated by GUSSI [59].

### Crystallization

Protein complexes were prepared by mixing corresponding purified proteins in 1:1 or 1:2 molar ratio in accordance with their binding stoichiometry in SEC buffer. Protein mixes were incubated overnight at 16 °C. Formed protein complexes were separated from unbound proteins by SEC in crystallization buffer (20 mM Tris, pH 7.8, 50 mM NaCl, 0.5 mM TCEP) using a Superdex 75 10/300 column. Central peak fractions corresponding to complex protein were pooled and concentrated to a concentration of ~ 300 µM. Complexes were analyzed by SDS-PAGE and LC-ESI-TOF-mass spectrometry before plate set-up. Crystallization was performed using the sitting drop vapor diffusion method at 20 °C with conditions shown in Supplementary Table 3. Viable crystals were mounted in mother liquor containing 22 % glycerol before being flash-frozen in liquid nitrogen. Diffraction data were collected at the Swiss Light Source and processed and scaled using XDS [60] and Aimless [61], respectively. All crystal structures were determined by molecular replacement using Phaser [62] with published structures with PDB IDs 3QYN, 3US0, 6FPB, 6S9S, 2Y9U, 3ZUV and 4A9Z as search models. Model rebuilding was performed using COOT [63] and REFMAC5 [64] for refinement. Crystal statistics are summarized in Supplementary Table 3.

### Size exclusion chromatography (SEC)

All size exclusion chromatography (SEC) experiments were carried out using an Äkta purifier system (Cytiva) with indicated buffers and columns. 10/300 columns were loaded with max. 8 mg protein with 0.5 ml/min flow rate at 4 °C. 16/600 columns were loaded with max. 80 mg protein with 1 ml/min flow rate at 4 °C.

### Cell survival assay (Cell viability assay)

T-REx-HeLa cells stably expressing DARPin C14, G4 or control DARPin as well as TAp63α separated by a T2A self-cleaving peptide as a linker were seeded into white Nunc 96-well microplates (Thermo Fisher Scientific). Protein expression was induced as described before. Twenty-four hours after induction cells were treated with different concentrations of doxorubicin (DOX) or DMSO for 6 h. The medium was exchanged to medium containing substrate and NanoLuc enzyme according to the manufacturer’s instructions using the RealTime-Glo MT assay kit (Promega). Luminescence was monitored continuously using a Spark plate reader (Tecan).

### Statistic and reproducibility

ITC measurements were performed twice, the determination of the *K*_D_ value is, however, based on a single measurement. The value and confidence intervals were obtained by SEDPHAT [58]. Pulldown experiments were performed in biological triplicates. All individual data points are shown in the corresponding figures. In addition, the bar diagram presents the mean value and the error bar the SD. Fluorescence anisotropy measurements were performed in triplicates. *K*_D_ and SD were determined by the program Prism (Version 8.2.1, GraphPad). Each pulldown from primary tissues was performed in triplicates with lysates derived from different mice. The cell survival assays were performed in triplicates. Each data point presents the mean value and the error bar the SD.

### Reporting summary

Further information on research design is available in the Nature Research Reporting Summary linked to this article.

## Supplementary information

Supplementary_Material

Supplementary Figure1

Supplementary Figure2

Supplementary Figure3

Supplementary Figure4

Supplementary Figure5

Supplementary Figure6

Supplementary Figure7

Supplementary Table1

Supplementary Table2

Supplementary Table3

Reporting Summary

PDB validation report 1

PDB validation report 2

PDB validation report 3

PDB validation report 4

## Data Availability

All data are fully available upon request. PDB accession codes for the four crystal structures are 7Z71, 7Z72, 7Z73, and 7Z7E.
