# Supplementary material for: Designed Ankyrin Repeat Proteins as a tool box for analyzing p63
Source: Cell Death Differ. 2022 Jun 18;29(12):2445–58. doi: 10.1038/s41418-022-01030-y (PMC9751120; doi:10.1038/s41418-022-01030-y)
Supplement: Supplementary file 1 — Supplementary_Material [file 41418_2022_1030_MOESM1_ESM.docx]

**Supplementary Material**

**Figure Captions**

**Supplementary Fig.1 Characterization of DBD-specific DARPins**

**A** Schematic representation of the domain structure of different p63 isoforms and of p53 and p73. TAD: transactivation domain; DBD: DNA-binding domain; TD: tetramerization domain; SAM: sterile α-motif domain; TID: transcriptional inhibitory domain. TA*p63α and GTAp63α contain at their respective N-termini additional sequences that further stabilize the dimeric conformation. ΔNp63α and TAp63γ isoforms form open tetrameric states as they either lack the TAD or the TID which are both necessary to form the closed dimeric state. The oligomeric state is symbolized on the right. The domain definition of used constructs is indicated as numbers corresponding to TAp63α numbering. **B** ITC measurements with the control DARPin and the DBD of p63 showing no binding. The top diagram displays the raw measurement and the bottom diagram shows the integrated heat per titration step**. C** Size exclusion chromatography of either DARPin C14 or G4 with p63 DBD. The elution volume of a sample containing both proteins show a shift to lower volume indicating the formation of a stable complex. A Superdex 75 10/300 column was used. **D** Pulldown experiments with different *in-vitro* translated p63 isoforms and immobilized control DARPin showing no unspecific binding. **E** Fluorescence anisotropy measurements of Alexa 488 labelled control DARPin and purified full‑length TAp63α and ΔNp63α isoforms. Size exclusion profiles with indicated peak fractions used for the experiment are shown in **F**. For TAp63α, a Superdex 200 16/600 and for ΔNp63α a Superose 6 16/600 column was used. **G** Detailed analysis of the interface of DARPin C14 with the p63 DBD showing the interacting side chains of the complex crystal structure analyzed by LigPlot^[63](#_ENREF_63)^. **H** Detailed analysis of the interface of DARPin G4 with the p63 DBD showing the interacting side chains of the complex crystal structure analyzed by LigPlot. **I** Sequence alignments of p63, p53 and p73 DBDs with amino acids highlighted involved in the interaction with DARPin C14 and G4 respectively. Hydrogen bonds are highlighted in red, hydrophobic contacts in blue, unique interactions of DARPin C14 are highlighted with a box and of DARPin G4 with a dashed box. **J** Sequence alignments of DARPin C14 and G4 with highlighted amino acids following the same color code as in (I). Unique interacting amino acids with the p63 DBD are highlighted with a box and numbers referring to numbers shown in (I). **K** Comparison of the DARPin C14-p63 DBD complex with a complex of the p63 DBD and DNA (PDB code: 3US1). The comparison shows that the DARPin C14 blocks the DNA binding interface of the p63 DBD. **L** Comparison of the DARPin G4-p63 DBD complex with a complex of the p63 DBD and DNA (PDB code: 3US1). The comparison shows that the DARPin G4 blocks the DNA binding interface of the p63 DBD.

**Supplementary Fig.2 Characterization of the Tetramerization domain-specific DARPin 8F1**

**A** Structures of the tetramerization domain of p63 (PDB code: 4A9Z) and the oligomerization domain of p53 (PDB code: 1SAF). Each monomer is colored differently. The p63 TD has one additional helix that crosses the tetramerization interface and stabilizes the tetrameric state. This additional helix is missing in p53. **B** ITC measurement with the control DARPin and the TD of p63 showing no binding. The top diagram displays the raw measurement and the bottom diagram shows the integrated heat per titration step. **C** Size exclusion chromatography of DARPin 8F1 with the p63 TD. The elution volume of a sample containing both proteins show a shift to lower volume indicating the formation of a stable complex. A Superdex 75 10/300 column was used. **D** Detailed view of the DARPin 8F1–p63 TD interface showing the most critical amino acid side chains involved in the interaction analyzed by LigPlot^[63](#_ENREF_63)^. **E** Crucial amino acids are also indicated within the sequences with hydrogen bonds highlighted in red, hydrophobic contacts in blue. **F** Sequence comparison of the p63 TD with the p53 TD as well as with the p73 TD. * indicates identical amino acids, : homologous amino acids and . a lower degree of homology between amino acids. Solid frames indicate residues that are recognized by the DARPin and that are not conserved in the sequences of p53 and p73, frames with broken lines those residues that are identical or homologous.

**Supplementary Fig.3 Characterization of the SAM domain-specific DARPin A5**

**A** ITC measurement with the control DARPin and the SAM domain of p63 showing no binding. The top diagram displays the raw measurement and the bottom diagram shows the integrated heat per titration step. **B** Size exclusion chromatography of DARPin A5 with the p63 SAM domain. The elution volume of a sample containing both proteins show a shift to lower volume indicating the formation of a stable complex. A Superdex 75 10/300 column was used. **C** Detailed view of the DARPin A5–p63 SAM domain interface showing the most critical amino acid side chains analyzed by LigPlot^[63](#_ENREF_63)^. **D** Crucial amino acids are also indicated within the sequences shown below the structure with hydrogen bonds highlighted in red, hydrophobic contacts in blue. **E** Sequence comparison of the p63 SAM domain with the p73 SAM domain. * indicates identical amino acids, : homologous amino acids and . a lower degree of homology between amino acids. Solid frames indicate residues that are recognized by the DARPin and that are not conserved in the sequences of p73, frames with broken lines those residues that are identical or homologous.

**Supplementary Fig.4 Experimental overview of control DARPin experiments and pulldowns with full‑length p53 family isoforms**

**A** ITC measurements of the control DARPin with the SAM domains of p63 and p73. Measurements with p53 are not shown, as p53 does not have a SAM domain. **B** ITC measurements of DBDs and **C** TDs of p53, p63 and p73 with control DARPin. **D** Western blot analysis of the pulldown experiments of *in‑vitro* translated TAp63α, ΔNp63α, TA*p63α, GTAp63α, TAp63γ as well as p53 and TAp73α and the indicated DARPins. On the left, the level of the different proteins in the lysate is indicated, on the right the pulled down protein levels. All p53 family members contain a myc‑tag at their N‑terminus that was used for detection with the anti‑myc antibody 4A6 (Millipore). The experiment was carried out in biological triplicates and one representative experiment is shown. **E** Bar diagrams of the results shown in the western blots in (D) for all selected DARPins. The data for the p63 isoforms are identical to the data shown in Fig. 1B, 2C and 3C, the results for p53 and TAp73α are added (n=3). **F** Same as (E) but for control DARPin (n=3). **G** Transactivation assay with p53, TAp63γ and TAp73β in H1299 cells. All three family members show high transcriptional activity. The transcriptional activity of all three p53 family members is not significantly influenced by increasing concentrations of co‑expressed control DARPin. The bar diagram shows the mean values and error bars the corresponding SD of four technical replicates. Statistical significance was assessed by ordinary one-way ANOVA.

**Supplementary Fig.5 Fluorescence labelling of all DARPins.**

**A** All DARPins used in this study were side directed labelled with the fluorophore Alexa 488 via a cysteine introduced at their C‑terminus via a glycine‑glycine linker. On the left, full chromatograms of runs on an HiTrap Q HP anion exchange column (Cytiva) are shown with a NaCl gradient from 50 mM to 600 mM. On the right the DARPin peaks of a subsequent run on a size exclusion chromatography Superdex 75 10/300 column (Cytiva) are shown. The 8F1-A488 DARPin seems to interact with the column material and shows therefore a higher elution volume. Absorption traces for excitation with both 280 nm and 493 nm wavelength are indicated. **B** Schematic representation of a DARPin with a cysteine introduced at the C‑terminus together with the Alexa Fluor 488 C5 Maleimide before reaction (left). Site-directed fluorophore labelled DARPin at its C‑terminal cysteine as used in the fluorescence anisotropy experiments (right).

**Supplementary Fig.6 DNA pulldown experiments with p53 and TAp73α and cell viability assay**

**A** DNA‑pulldown experiments with p53, **B** TAp73α and an immobilized DNA oligomer containing the 20bp binding site of the human p21 promoter. Pre-incubation of the indicated proteins with DARPin C14 or DARPin G4 significantly inhibits interaction with the DNA oligomer while a control DARPin does not significantly prevent binding. Corresponding western blot results are shown above the bar diagram. The relative pulldown efficiency as normalized to no DARPin is shown on the y-axis (n=3). The bar diagram shows the mean values and error bars show the corresponding SD of three biological replicates. Statistical significance was assessed by ordinary one-way ANOVA. **C** Schematic overview of the fusion construct design and cleaving mechanism. DARPins C14, G4 or control DARPin were fused N-terminal to TAp63α with a T2A peptide in between. The T2A peptide is a viral oligopeptide derived from thosea asigna virus which causes the ribosome to skip the formation of a glycyl-prolyl peptide bond due to steric hindrance. This process is called ribosome skipping and causes the cleavage of the translated peptide from its downstream peptide leading to the translation of separated DARPin and TAp63α. **D** Timeline of viability assay over 89h after 0.5 µM or **E** 0.7 µM DOX treatment showing reduction of the number of viable cells of a TAp63α and control DARPin expressing stable cell line compared to cells stably expressing TAp63α and either C14 or G4. All data are normalized to treatment with DMSO only. The standard deviations obtained from biological triplicates are shown.

**Supplementary Table 1** Overview of the ITC parameters of the interaction between the selected DARPins and target domains.

**Supplementary Table 2** Overview of the fluorescence anisotropy parameters of the interaction between the selected DARPins and target proteins.

**Supplementary Table 3** Summary of DARPin-p63 domain complex crystal structure data collection and refinement statistics.
