# Supplementary material for: Designed Ankyrin Repeat Proteins as a tool box for analyzing p63
Source: Cell Death Differ. 2022 Jun 18;29(12):2445–58. doi: 10.1038/s41418-022-01030-y (PMC9751120; doi:10.1038/s41418-022-01030-y)

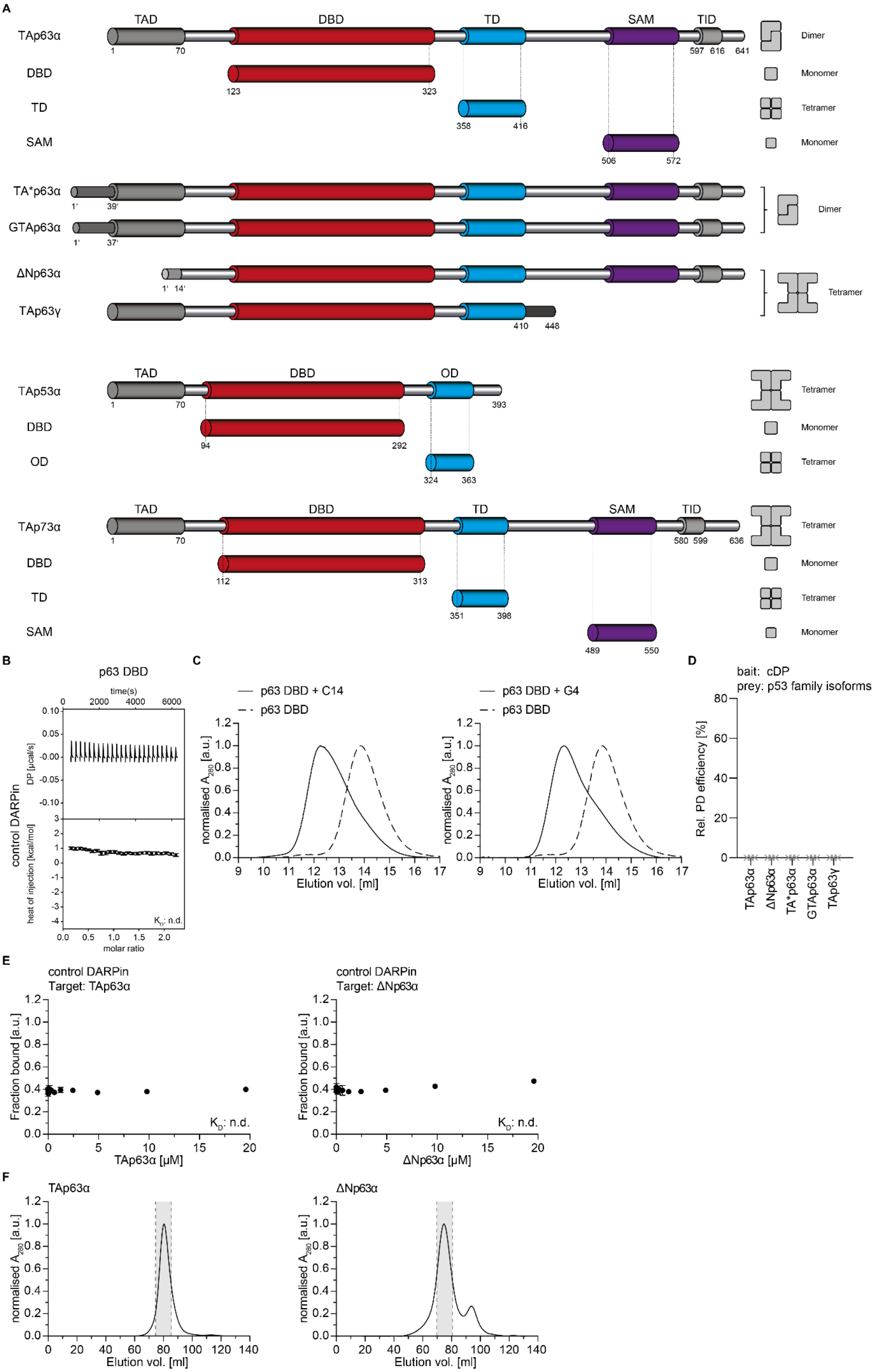

A
TAD
DBD
TD
SAM
TID
TAp63α
DBD
TD
SAM
TA*p63α
GTAp63α
ΔNp63α
TAp63γ
Dimer
Monomer
Tetramer
Monomer
Dimer
Tetramer
OD
TAp53α
DBD
OD
Tetramer
Monomer
Tetramer
TAp73α
DBD
TD
SAM
Tetramer
Monomer
Tetramer
Monomer
B
p63 DBD
control DARPin
time(s)
DP [µcal/s]
heat of injection [kcal/mol]
molar ratio
K_D: n.d.
C
p63 DBD + C14
p63 DBD
p63 DBD + G4
p63 DBD
normalised A280 [a.u.]
Elution vol. [ml]
D
bait: cDP
prey: p53 family isoforms
Rel. PD efficiency [%]
TAp63α
ΔNp63α
TA*p63α
GTAp63α
TAp63γ
E
control DARPin
Target: TAp63α
control DARPin
Target: ΔNp63α
Fraction bound [a.u.]
K_D: n.d.
TAp63α [µM]
ΔNp63α [µM]
F
TAp63α
ΔNp63α
normalised A280 [a.u.]
Elution vol. [ml]

G

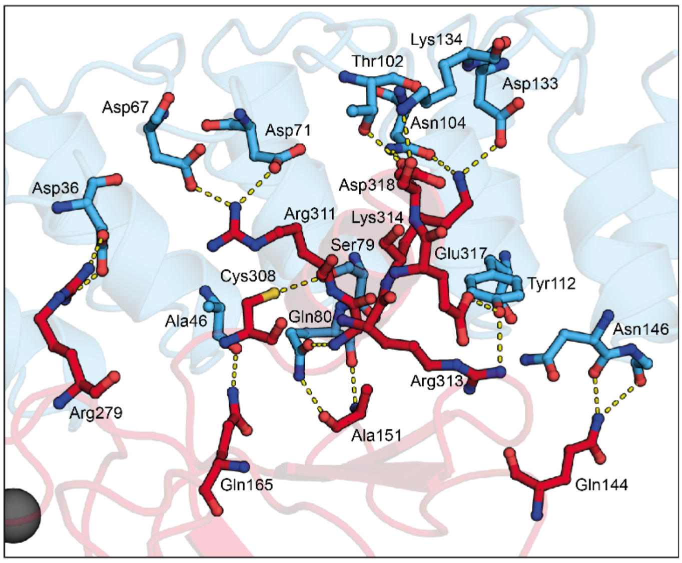

Lys134
Thr102
Asp133
Asp67
Asp71
Asn104
Asp36
Asp318
Arg311
Lys314
Ser79
Glu317
Cys308
Tyr112
Ala46
Gln80
Asn146
Arg313
Arg279
Ala151
Gln165
Gln144

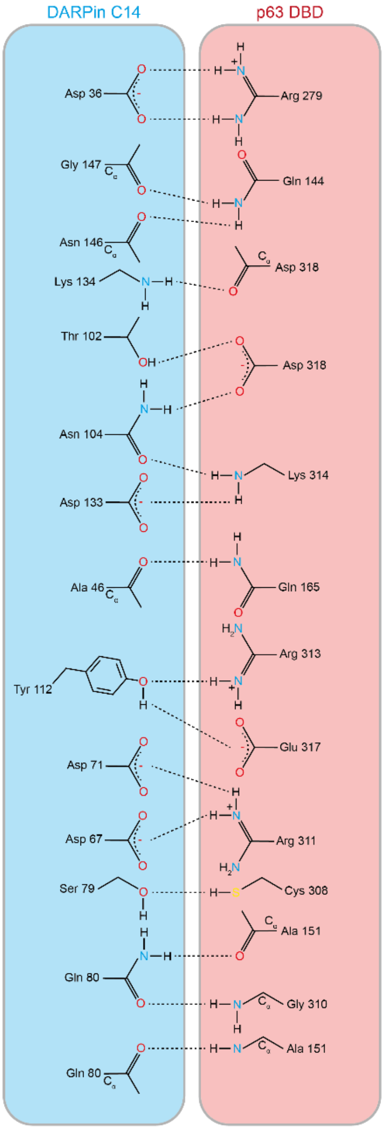

DARPin C14
p63 DBD
Asp 36
Arg 279
Gly 147
Gln 144
Asn 146
Lys 134
Asp 318
Thr 102
Asp 318
Asn 104
Lys 314
Asp 133
Ala 46
Gln 165
Arg 313
Tyr 112
Glu 317
Asp 71
Asp 67
Arg 311
Ser 79
Cys 308
Ala 151
Gln 80
Gly 310
Ala 151
Gln 80

H

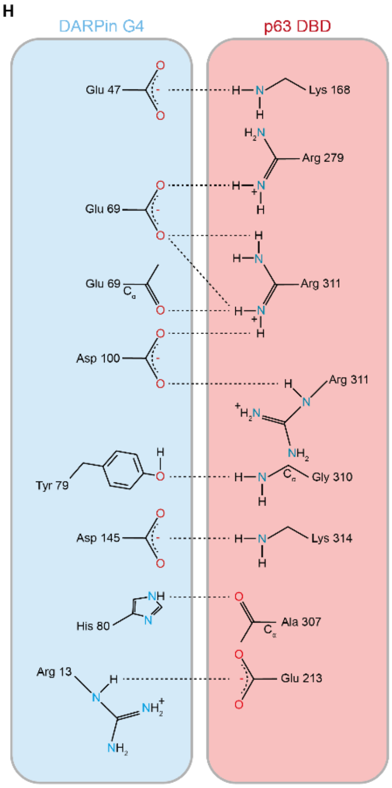

DARPin G4
p63 DBD
Glu 47
Lys 168
Arg 279
Glu 69
Glu 69
Arg 311
Asp 100
Arg 311
Tyr 79
Gly 310
Asp 145
Lys 314
His 80
Ala 307
Arg 13
Glu 213

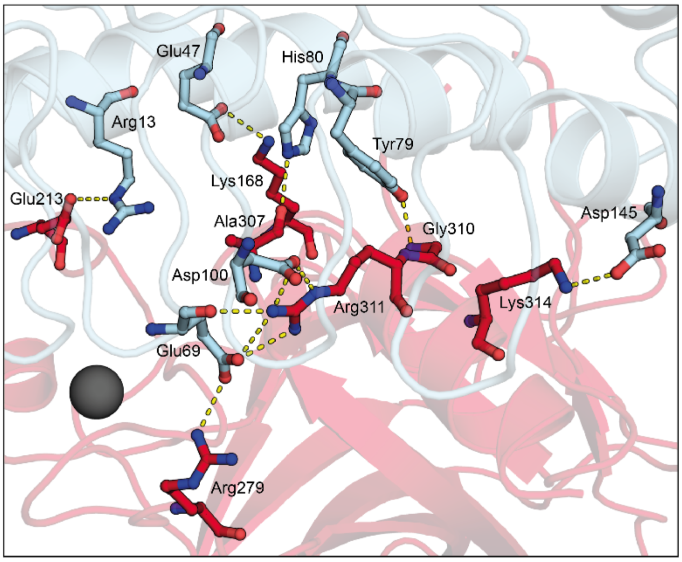

Glu47
His80
Arg13
Tyr79
Lys168
Glu213
Ala307
Gly310
Asp145
Asp100
Arg311
Lys314
Glu69
Arg279

I

```
                1          2
p63 123-223
C14          SPAIPSNTDYPGPHSFDVSFQQSSTAKSATWTYSTELKKLYCQIAKTCPIQIKVMTPPPQGAVIRAMPVYKKAEHVTEVVKRCPNHELSREFNEGQIAPPS
G4           SPAIPSNTDYPGPHSFDVSFQQSSTAKSATWTYSTELKKLYCQIAKTCPIQIKVMTPPPQGAVIRAMPVYKKAEHVTEVVKRCPNHELSREFNEGQIAPPS
             :  :**:. * * : * : * :*.****.* ***   *:*::**:*****:*: * : ** *: :*** :**:::*:*:**:***:**    :  ..  ** .

p53 94-192   SSSVPSQKTYQGSYGFRLGFLHSGTAKSVTCTYSPALNKMFCQLAKTCPVQLWVDSTPPPGTRVRAMAIYKQSQHMTEVVRRCPHHERCSD--SDGLAPPQ
p73 112-212  APVIPSNTDYPGPHHFEVTFQQSSTAKSATWTYSPLLKKLYCQIAKTCPIQIKVSTPPPPGTAIRAMPVYKKAEHVTDVVKRCPNHELGRDFNEGQSAPAS
```

```
p63 224-323
C14          HLIRVEGNSHAQYVEDPITGRQSVLVPYEPPQVGTEFTTVLYNFMCNSSCVGGMNRRPILIIVTLETRDGQVLGRRCFEARICACPGRDRKADEDSIRKQ
G4           HLIRVEGNSHAQYVEDPITGRQSVLVPYEPPQVGTEFTTVLYNFMCNSSCVGGMNRRPILIIVTLETRDGQVLGRRCFEARICACPGRDRKADEDSIRKQ
             ********   :*::*  * *:**:******:**:: **: **:******:********* *:***  .*::***..** *:********:::*:  *::

p53 193-292  HLIRVEGNLRVEYLDDRNTFRHSVVVPYEPPEVGSDCTTIHYNYMCNSSCMGGMNRRPILTIITLEDSSGNLLGRNSFEVRVCACPGRDRRTEEENLRKK
p73 213-313  HLIRVEGNNLSQYVDDPVTGRQSVVVPYEPPQVGTEFTTILYNFMCNSSCVGGMNRRPILIIITLEMRDGQVLGRRSFEGRICACPGRDRKADEDHYREQ
```

■ Hydrogen Bonds
■ Hydrophobic Contacts
□ Unique interactions of DARPin C14
⬚ Unique interactions of DARPin G4

J

```
                    1        10        20        30        40        50        60        70        2
                                                                                                     80
C14     1-80   GSDLGKKLLEAAQIGQLDEVRILMANGADVNASDTDGLTPLHLAAASGHLEIVEVLLKTGADVNATDKWGDTPLHLAASQ
G4      1-80   GSDLGKKLLEAARAGQDDEVRILMANGADVNAADHSGDTPLHLAAMEGHLEIVEVLLKTGADVNAHDLEGYTPLHLAAYH
               ************: ** ***************:* .* ******* .****************** *  * ******* :

                                                                   1
                        90       100       110       120       130       140       150
C14     81-159 GHLEIVEVLLKAGADVNATDFTGNTPLHLAAYIGHLEIVEVLLKHGADVNAQDKFGKTPFDLAIDNGNEDIAEVLQKAA
G4      81-159 GHLEIVEVLLKAGADVNAWDSYGYTPLHLAAMTGHLEIVEVLLKHGADVNAQDKFGKTPFDLAIDNGNEDIAEVLQKAA
               ****************** *  * *******  ************************************************
```

■ Hydrogen Bonds
■ Hydrophobic Contacts

K

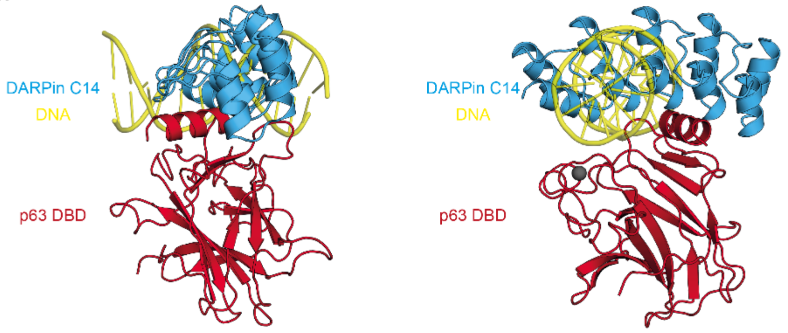

L

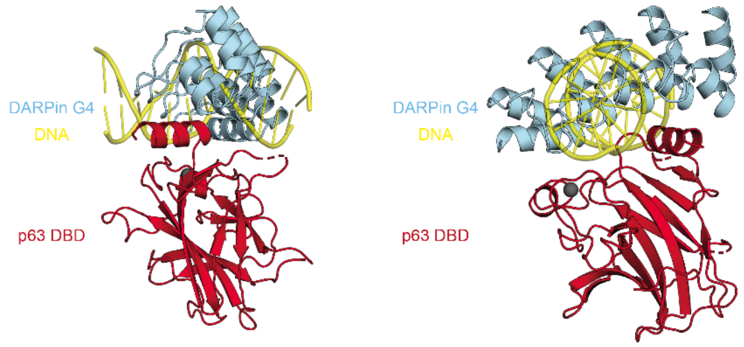

Supplement: Supplementary file 2 — Supplementary Figure1 [file 41418_2022_1030_MOESM2_ESM.pdf]
