# Supplementary figures and images for: Designed Ankyrin Repeat Proteins as a tool box for analyzing p63
Source: Cell Death Differ. 2022 Jun 18;29(12):2445–58. doi: 10.1038/s41418-022-01030-y (PMC9751120; doi:10.1038/s41418-022-01030-y)

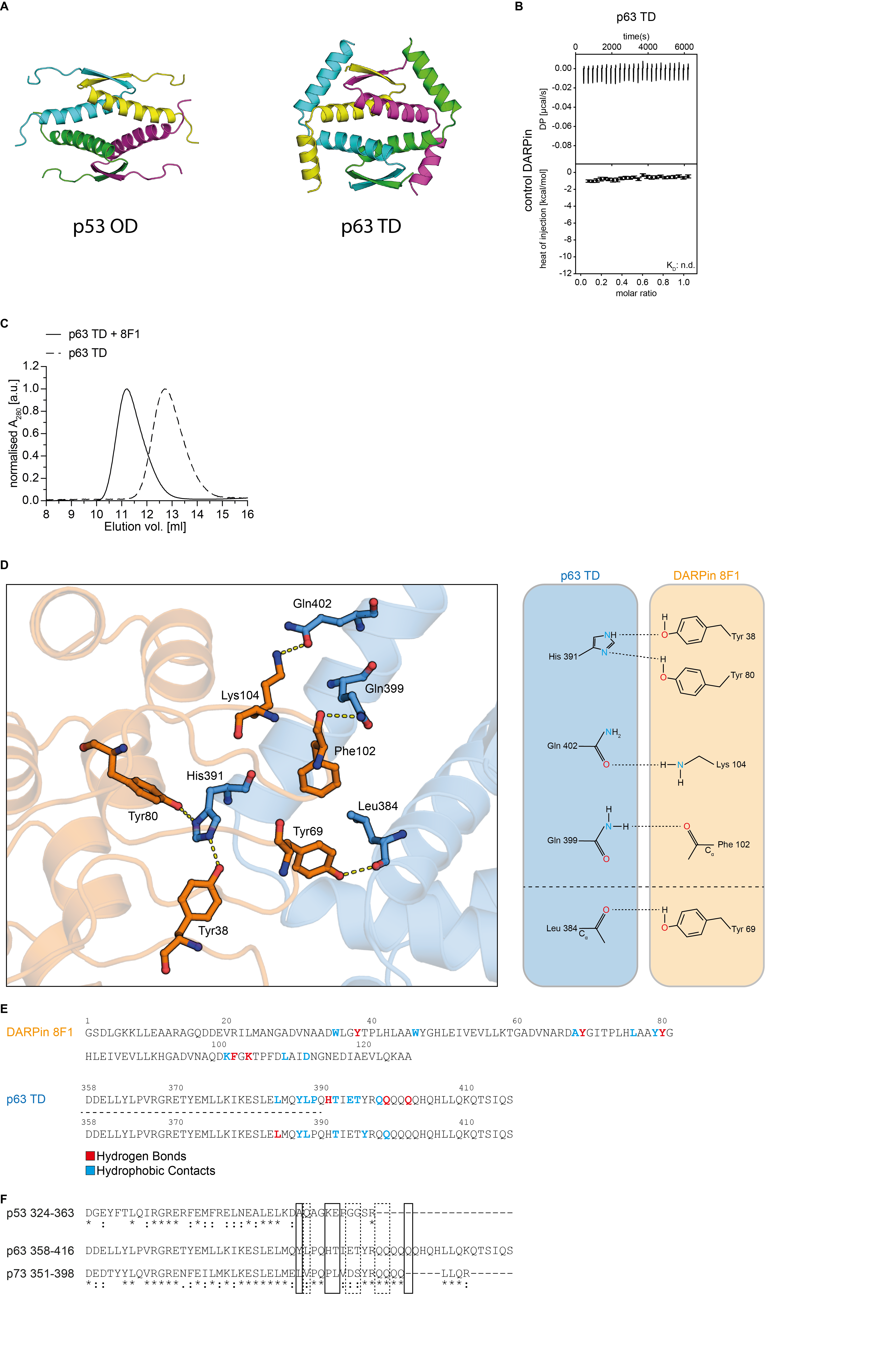

Supplement: Supplementary file 3 — Supplementary Figure2 [file 41418_2022_1030_MOESM3_ESM.png]

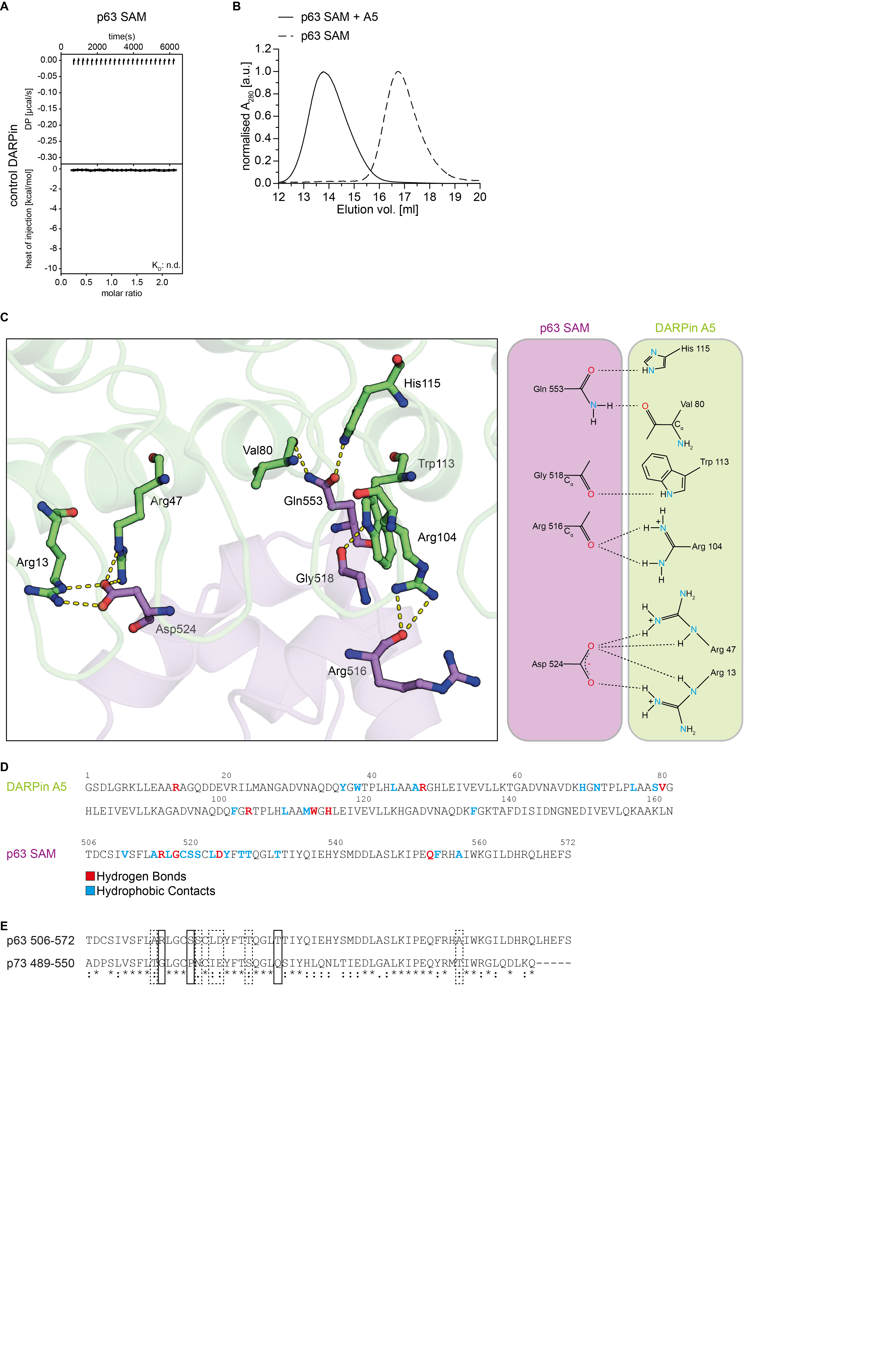

Supplement: Supplementary file 4 — Supplementary Figure3 [file 41418_2022_1030_MOESM4_ESM.png]

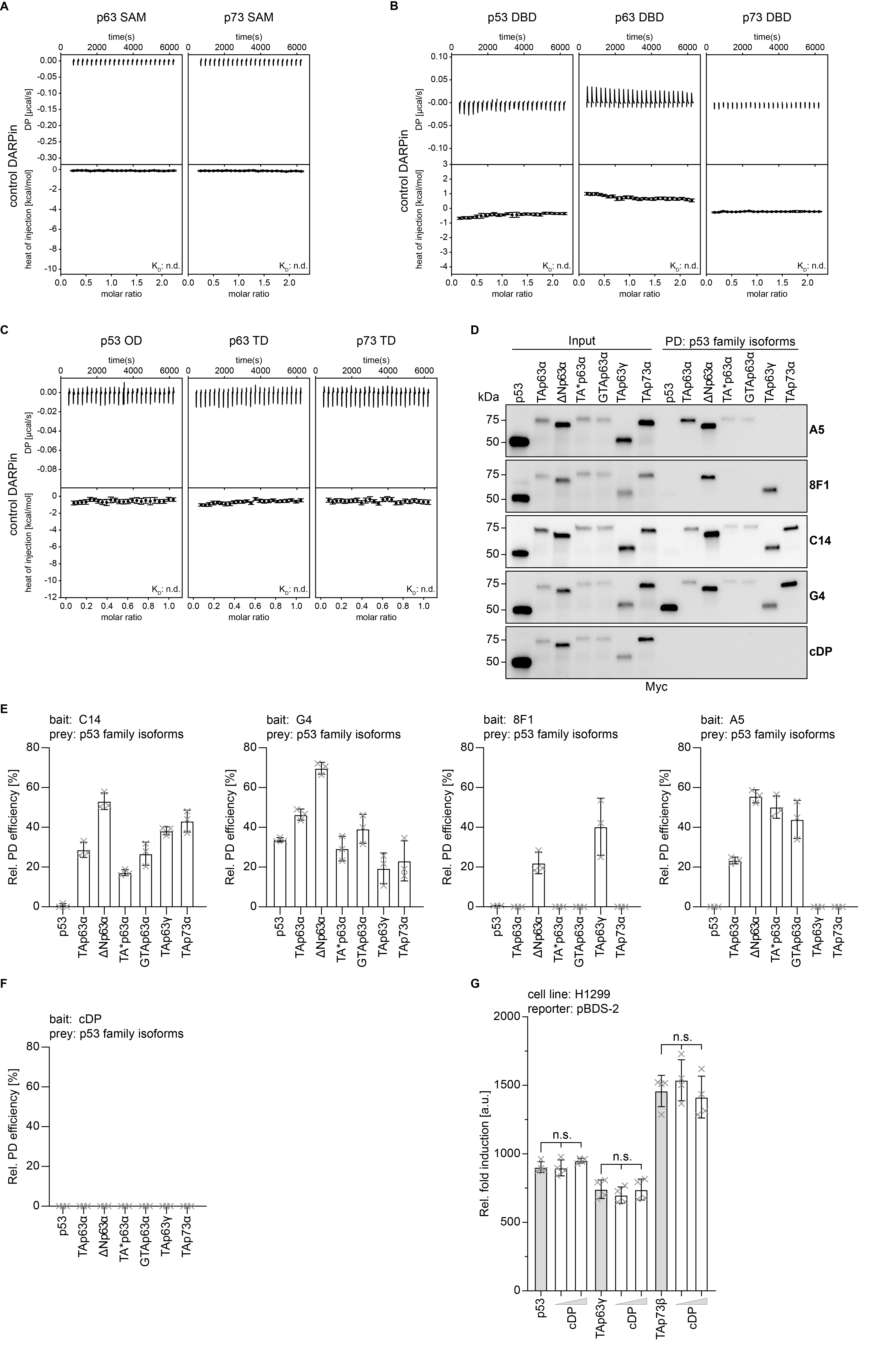

Supplement: Supplementary file 5 — Supplementary Figure4 [file 41418_2022_1030_MOESM5_ESM.png]

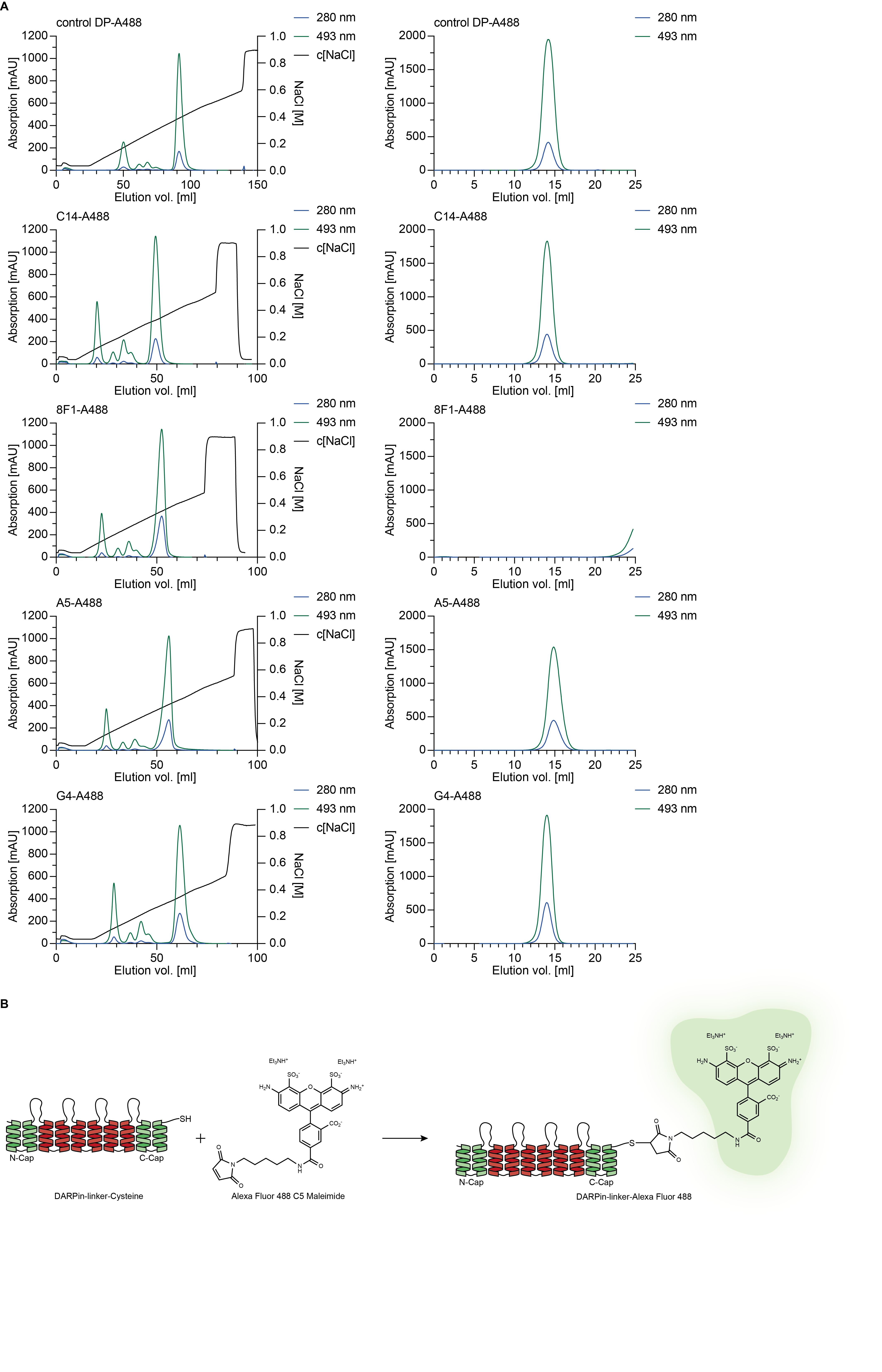

Supplement: Supplementary file 6 — Supplementary Figure5 [file 41418_2022_1030_MOESM6_ESM.png]

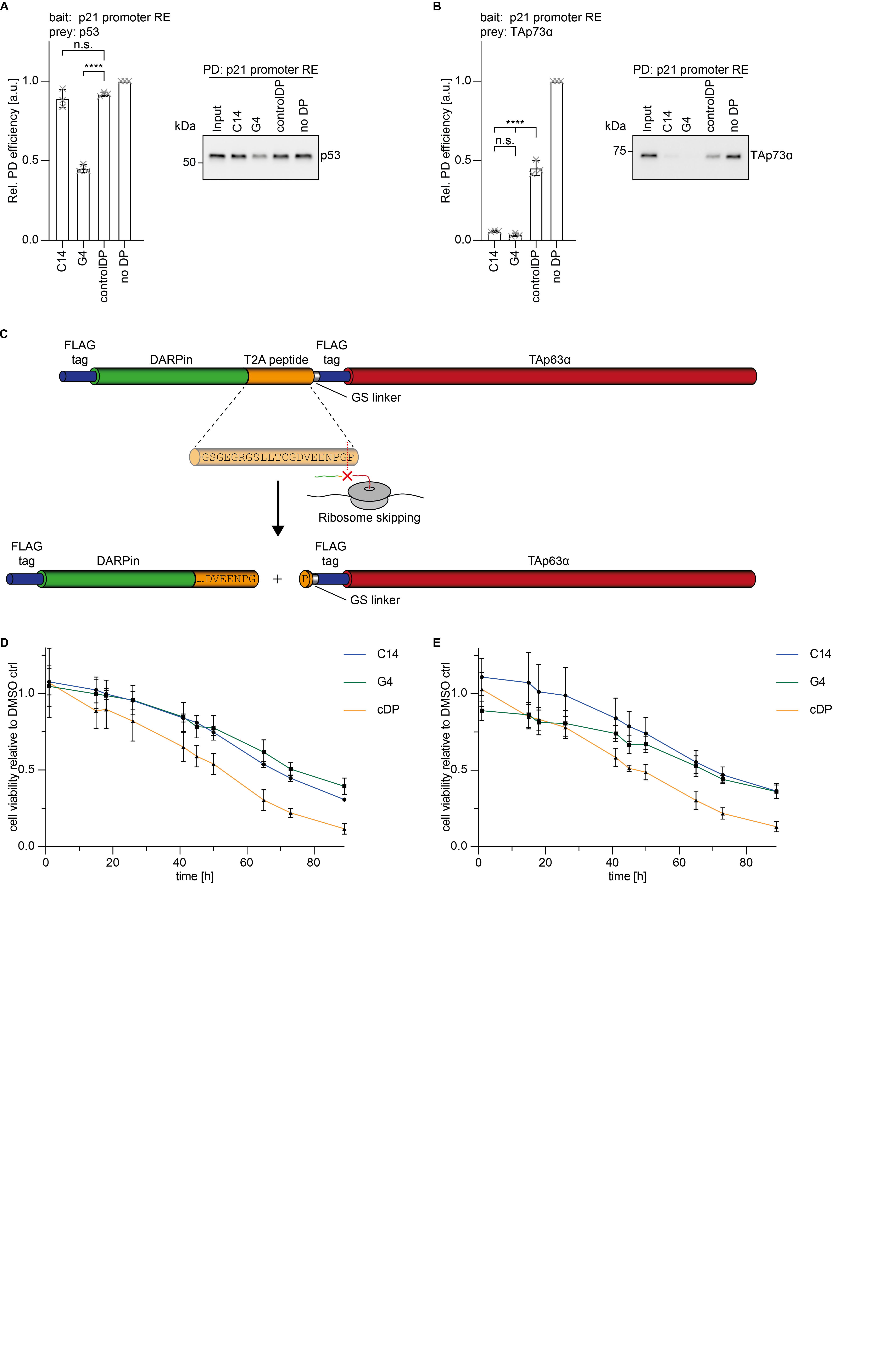

Supplement: Supplementary file 7 — Supplementary Figure6 [file 41418_2022_1030_MOESM7_ESM.png]
