# Supplementary material for: Designed Ankyrin Repeat Proteins as a tool box for analyzing p63
Source: Cell Death Differ. 2022 Jun 18;29(12):2445–58. doi: 10.1038/s41418-022-01030-y (PMC9751120; doi:10.1038/s41418-022-01030-y)

Raw data figure 1F

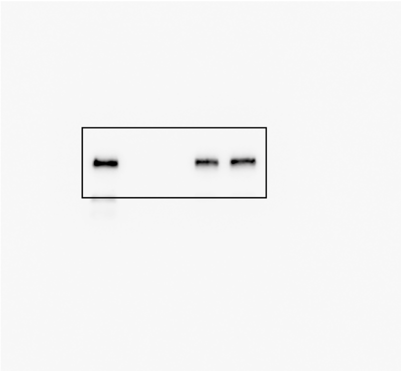

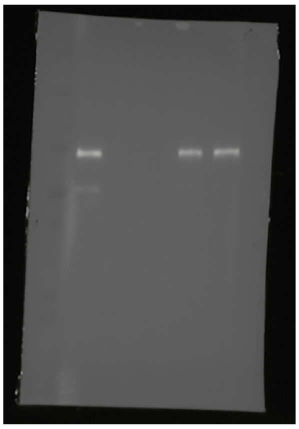

ΔNp63α Replicate 1

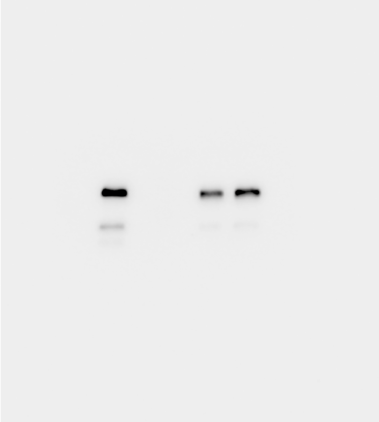

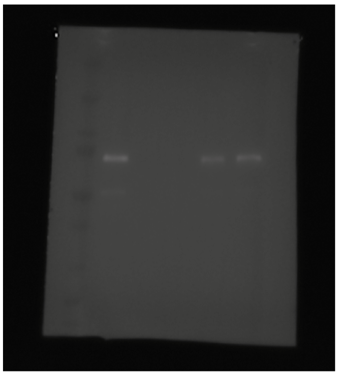

ΔNp63α Replicate 2

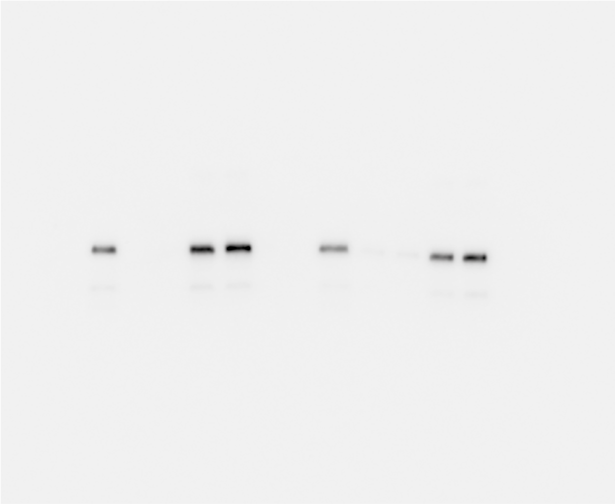

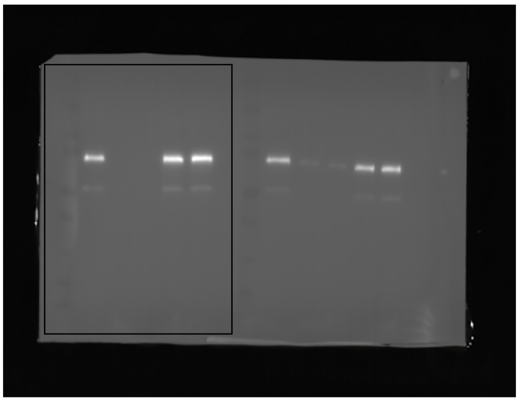

ΔNp63α Replicate 3

Raw data figure 1G

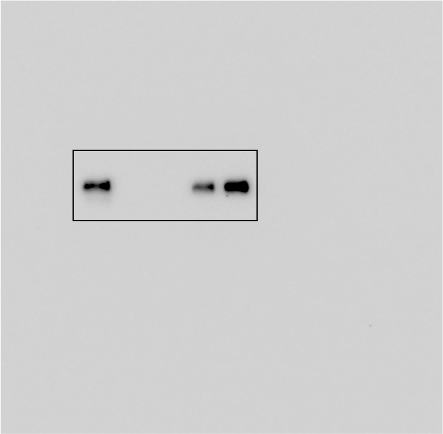

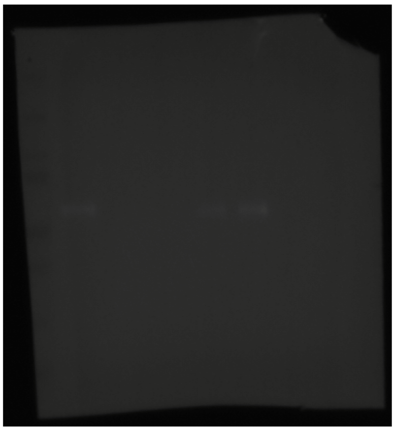

TAp63γ Replicate 1

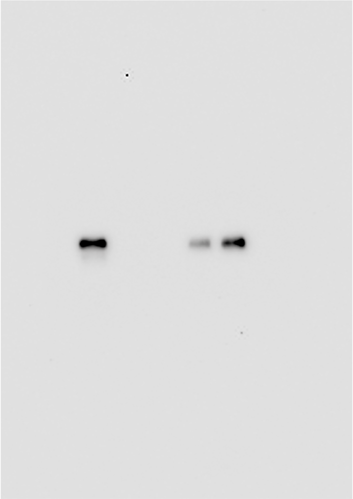

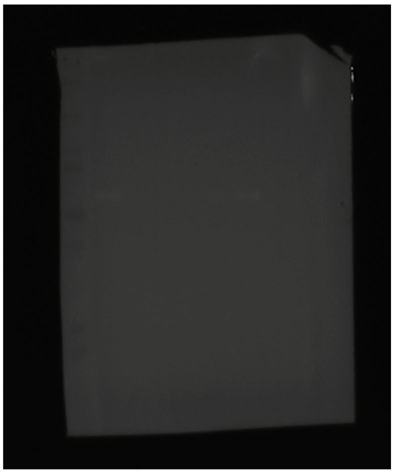

TAp63γ Replicate 2

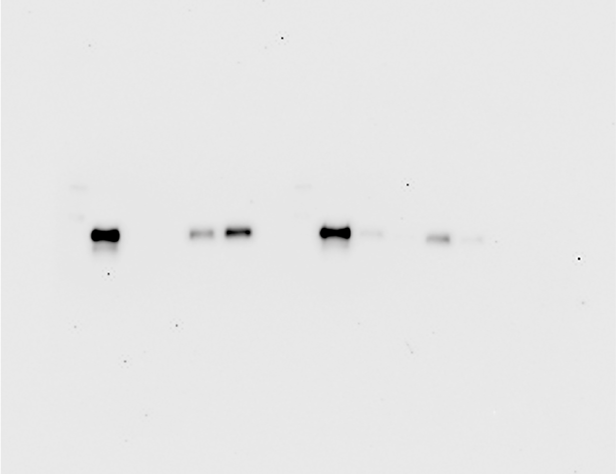

TAp63γ Replicate 3

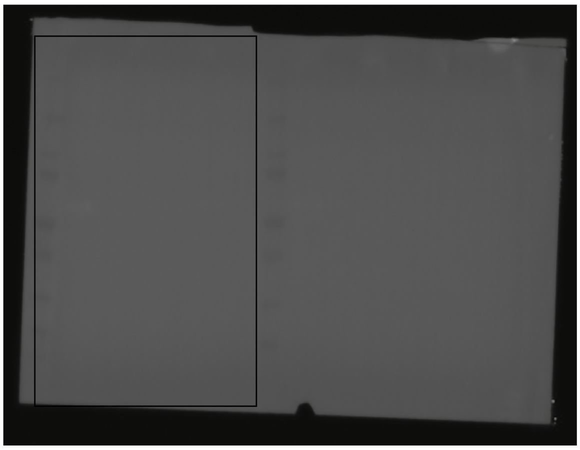

Raw data figure 6E Vinculin

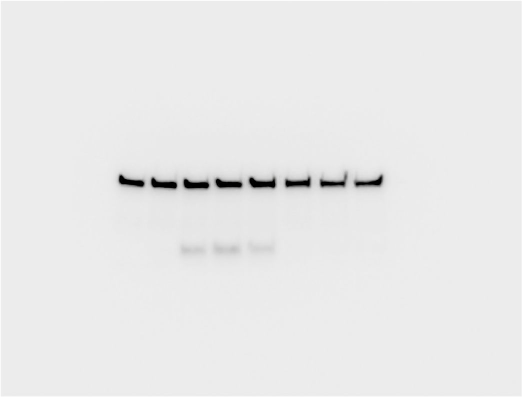

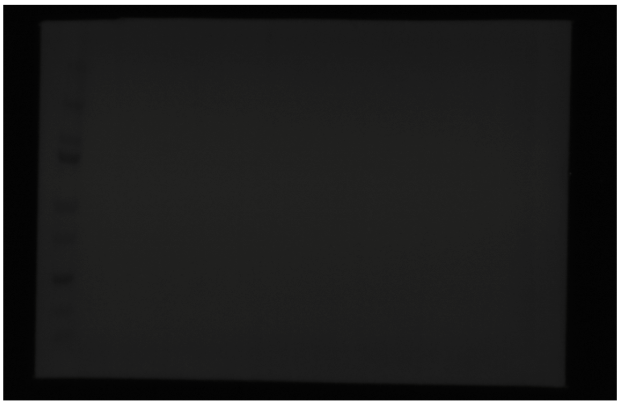

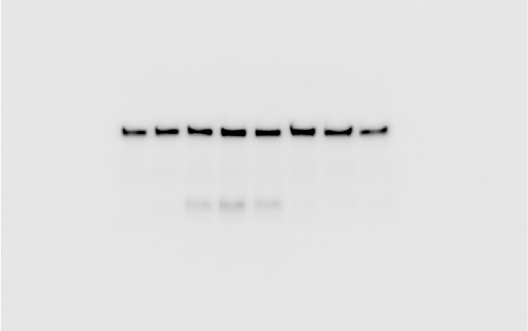

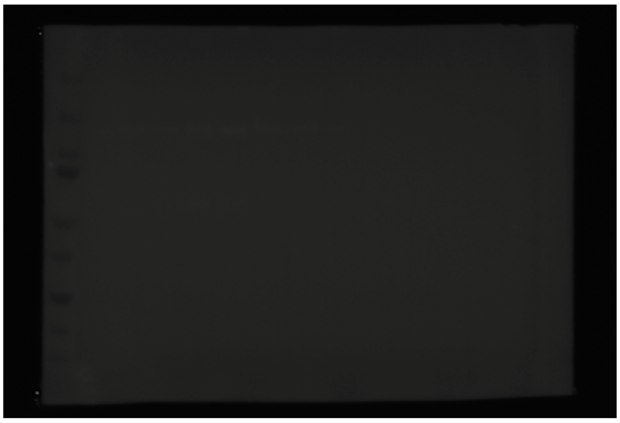

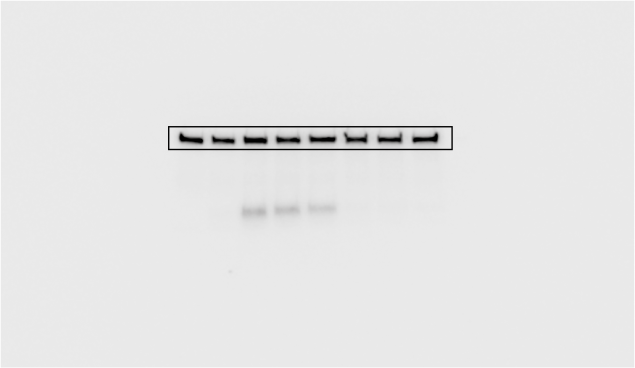

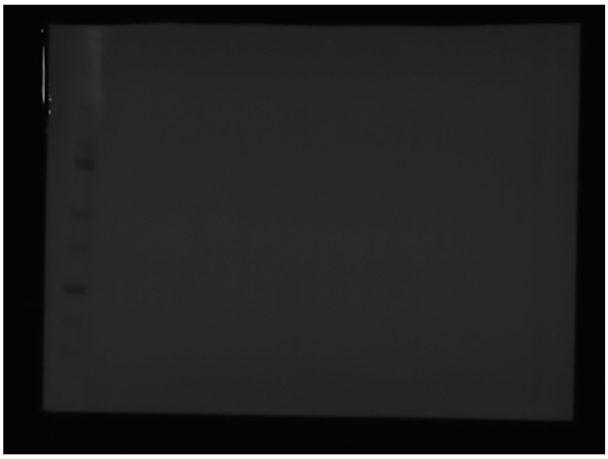

Raw data figure 6E TAp63γ

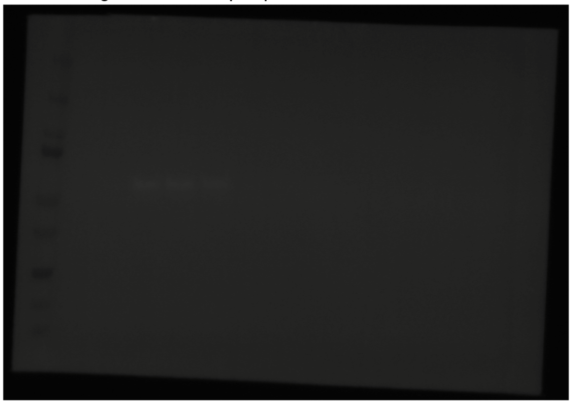
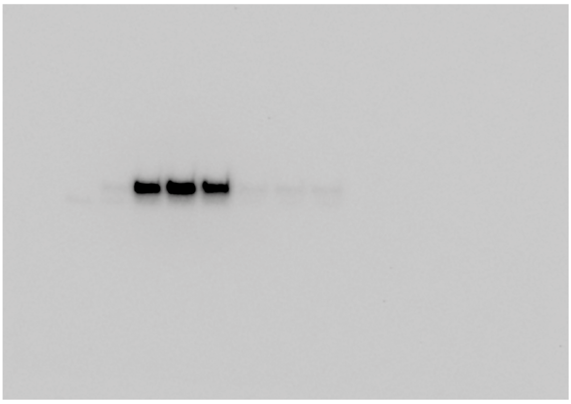

Replicate 1

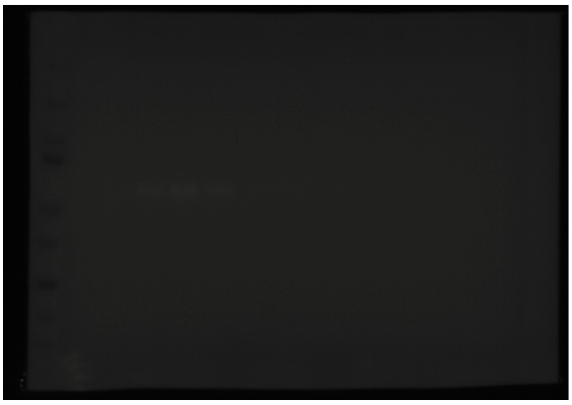
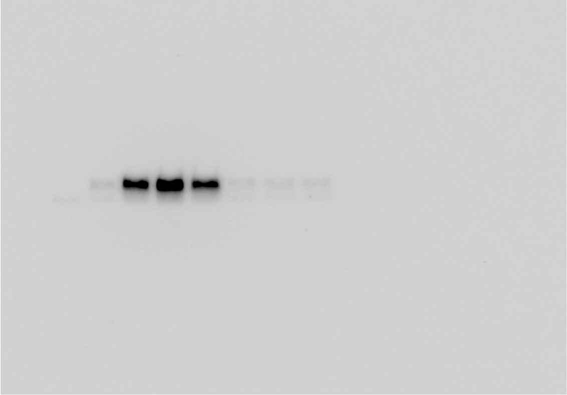

Replicate 2

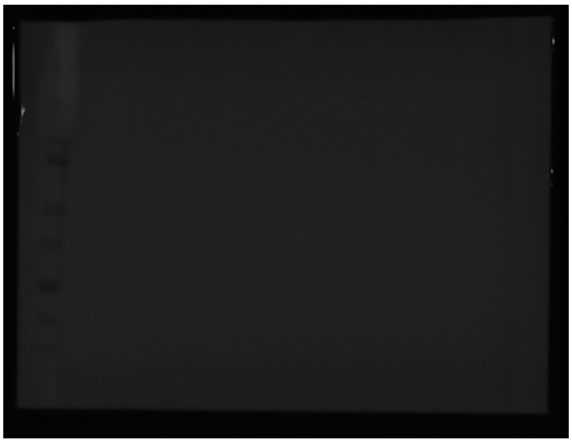
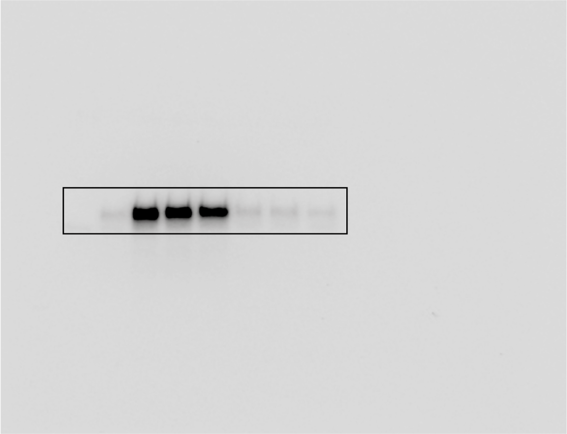

Replicate 3

Raw data figure 6E TAp63γ - longer exposure

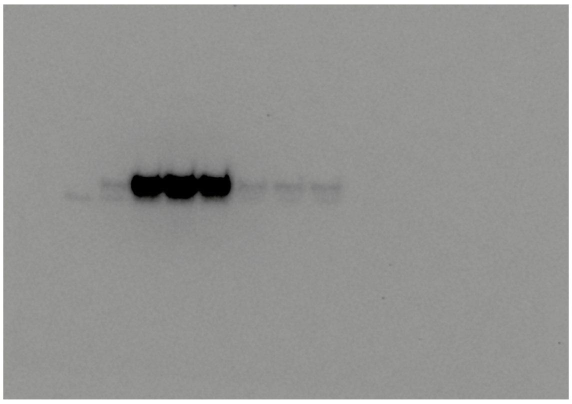

Replicate 1

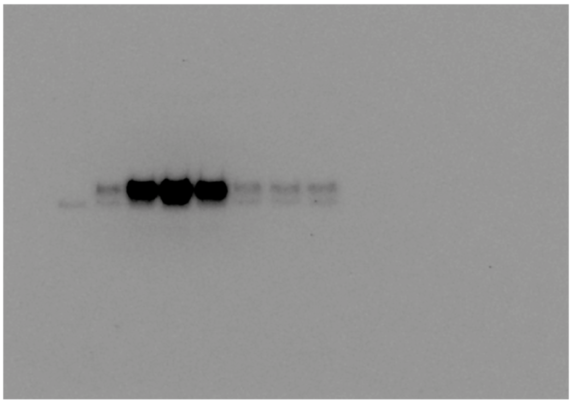

Replicate 2

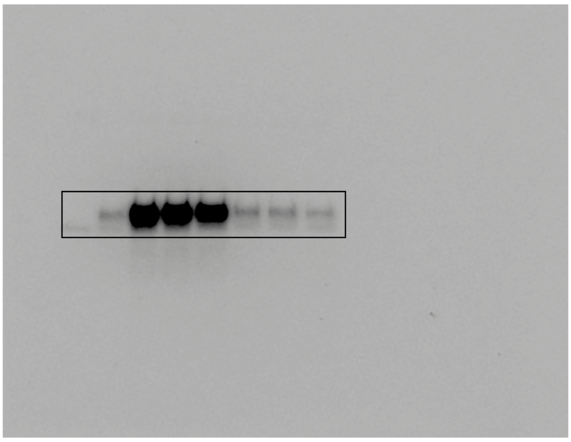

Replicate 3

Raw data figure 6F Vinculin

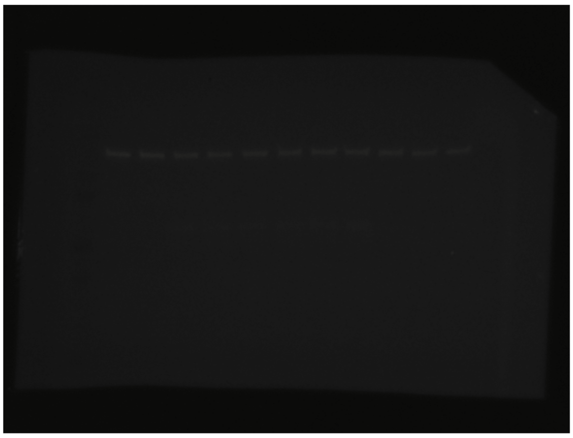

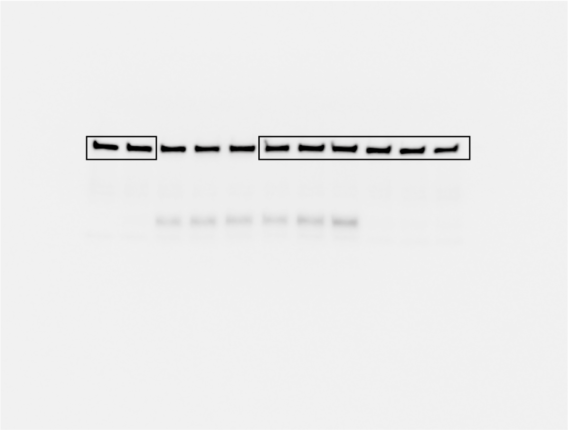

Replicate 1

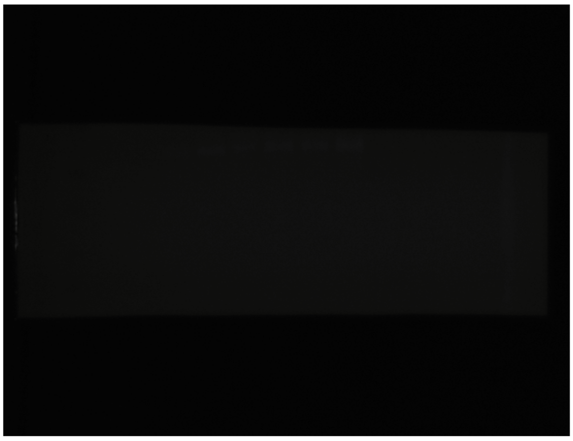

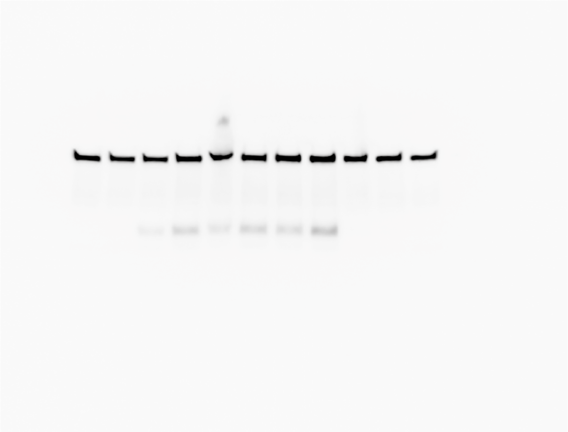

Replicate 2

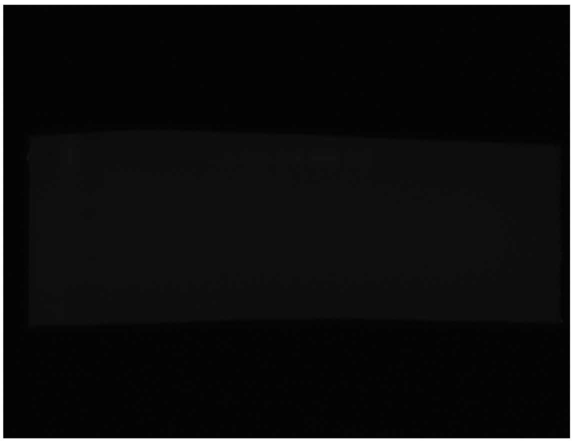

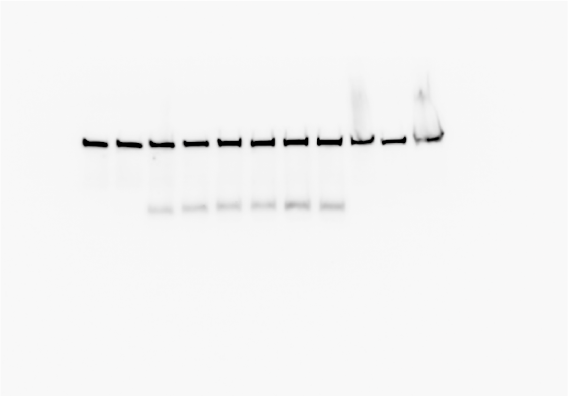

Replicate 3

Raw data figure 6F TAp63γ

longer exposure

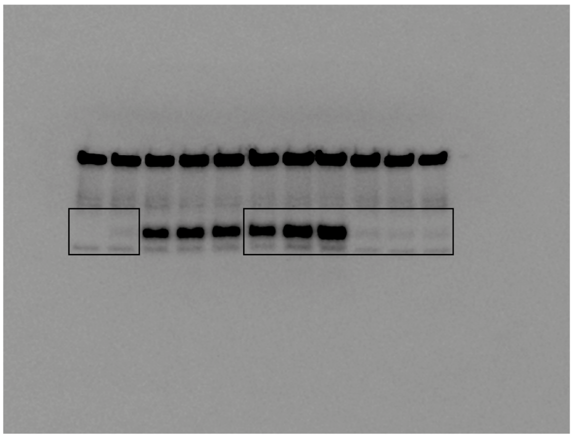

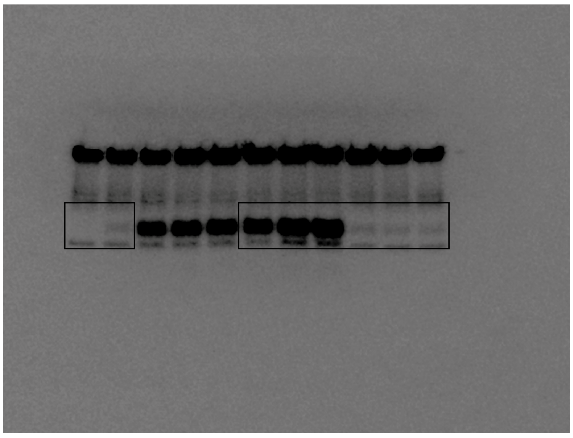

Replicate 1

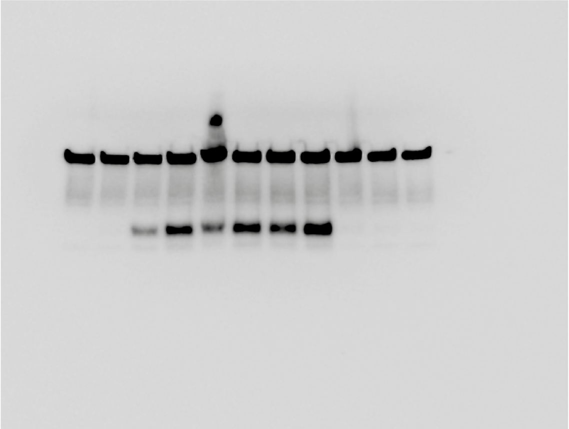

Replicate 2

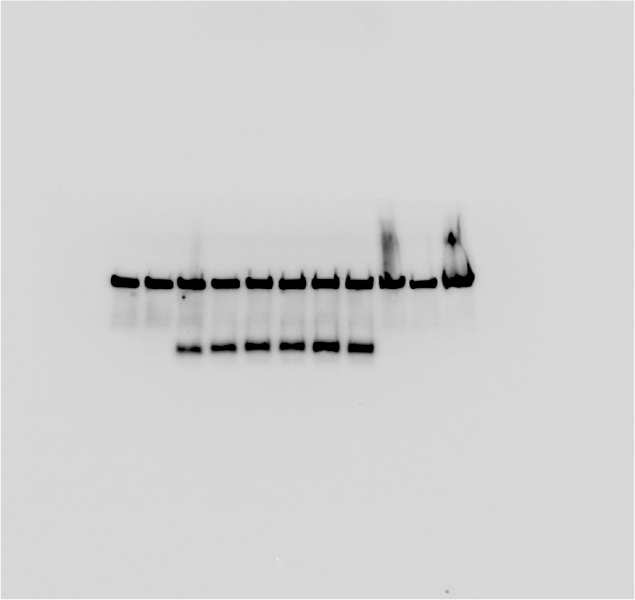

Replicate 3

Raw data figure 6l

Vinculin

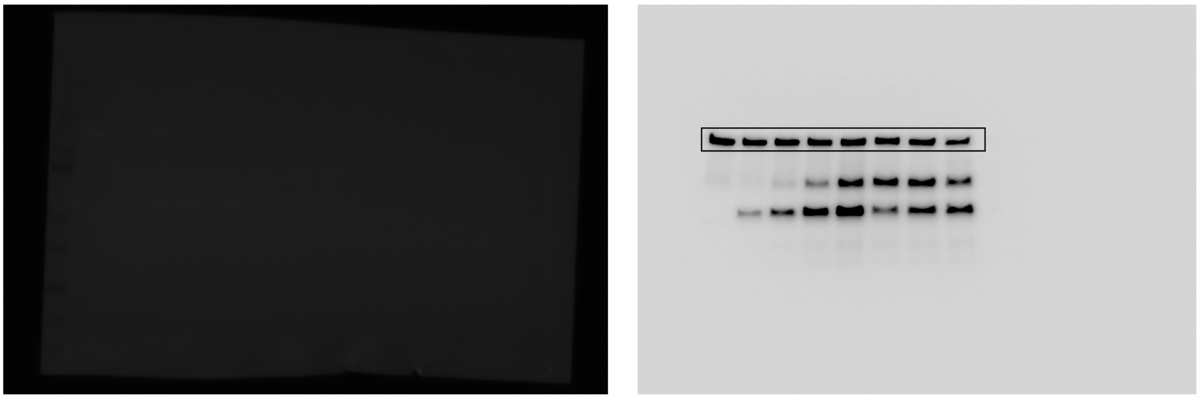

Replicate 1

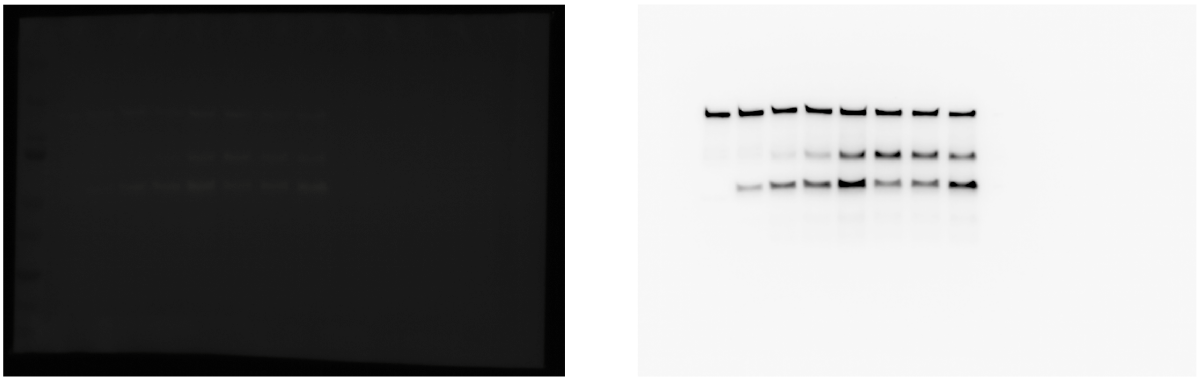

Replicate 2

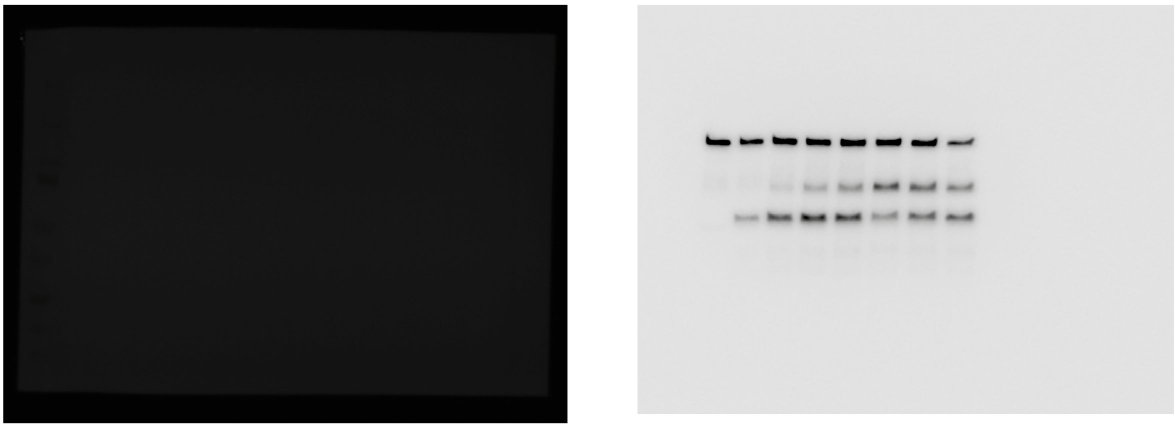

Replicate 3

Raw data figure 6l

ΔNp63α/p53

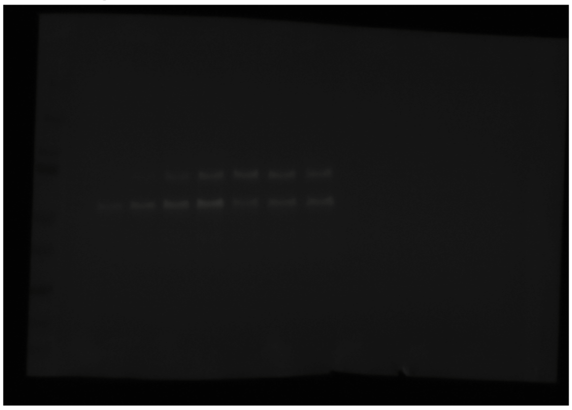

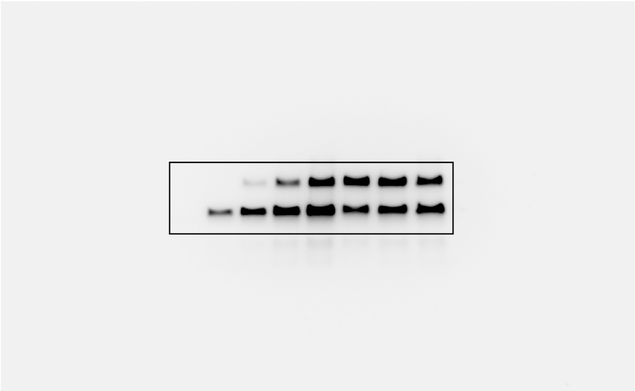

Replicate 1

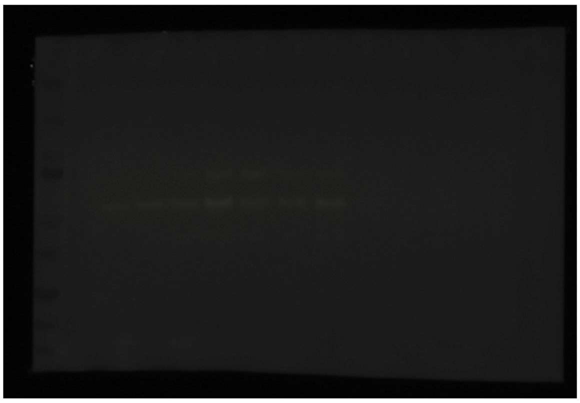

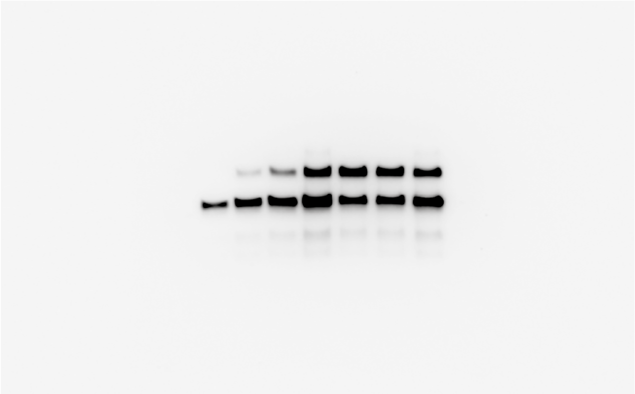

Replicate 2

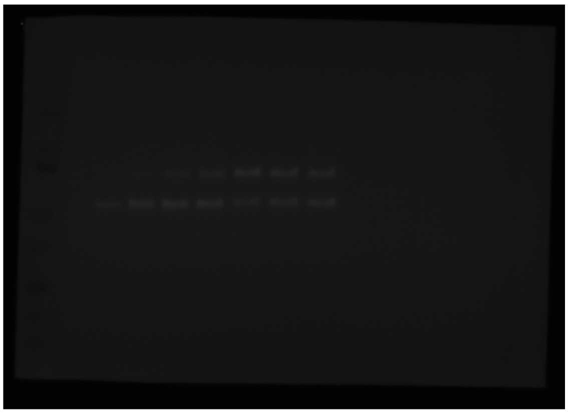

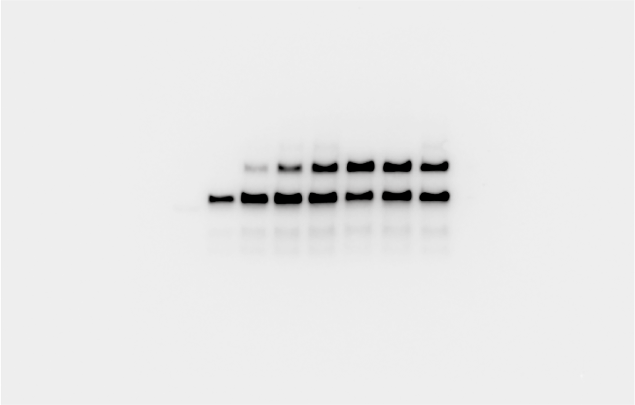

Replicate 3

Raw data figure 6M

p63

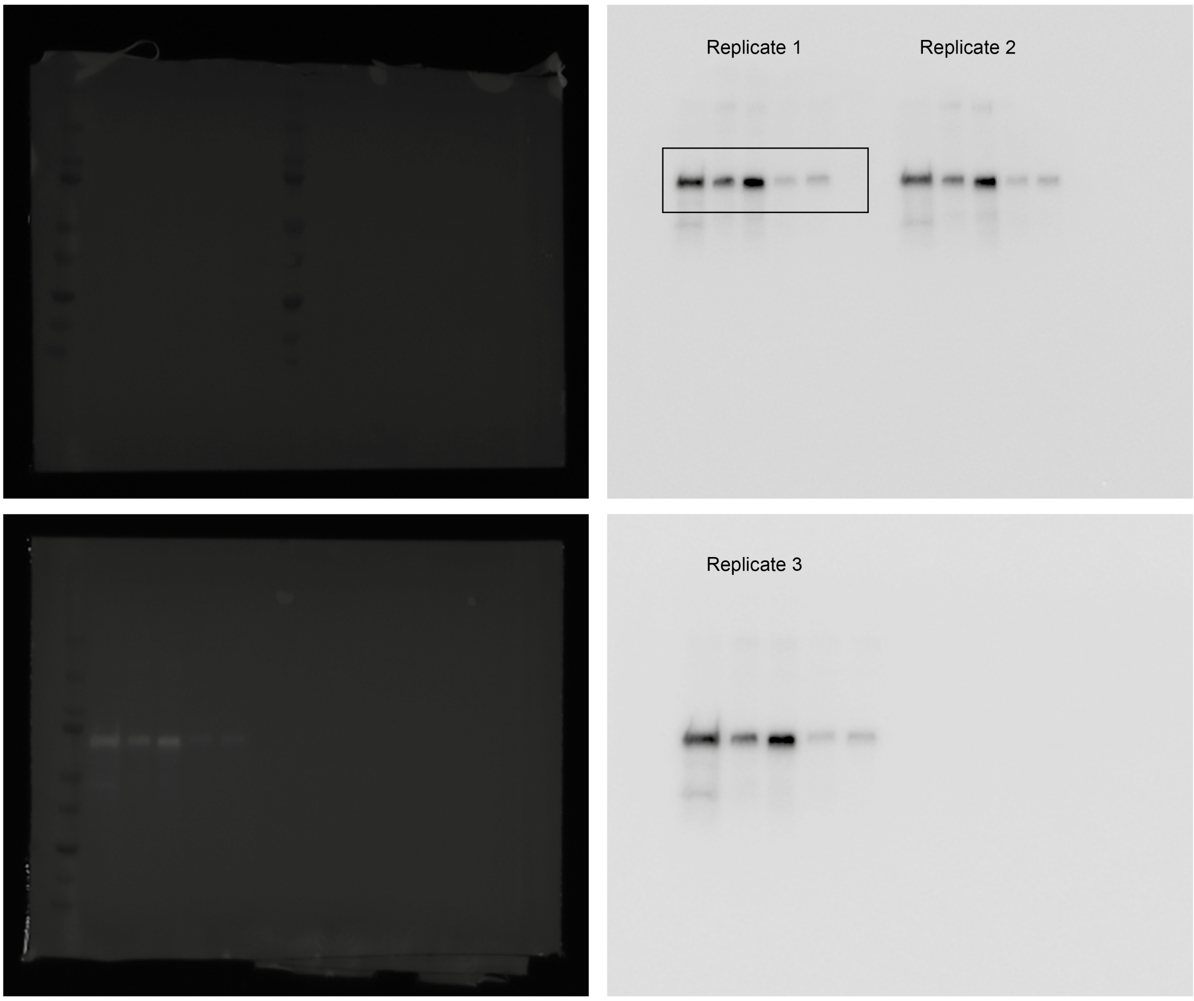

Raw data figure 6N

p63

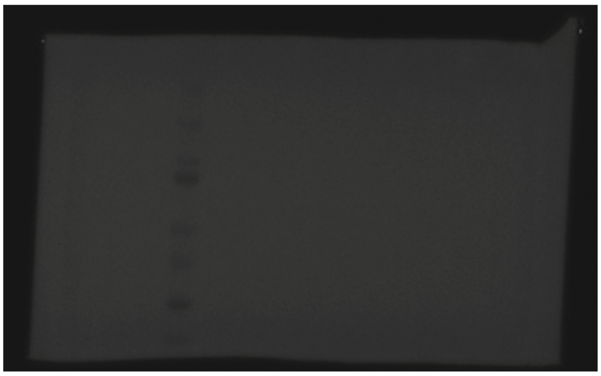
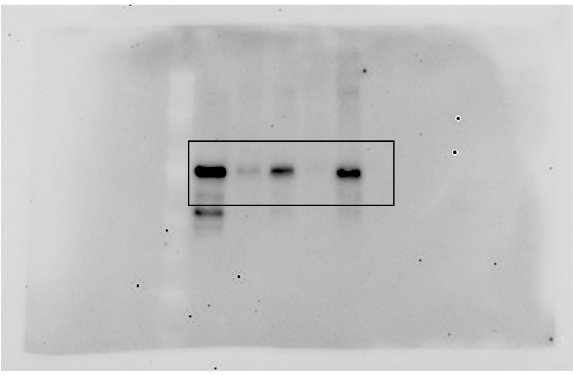

Replicate 1

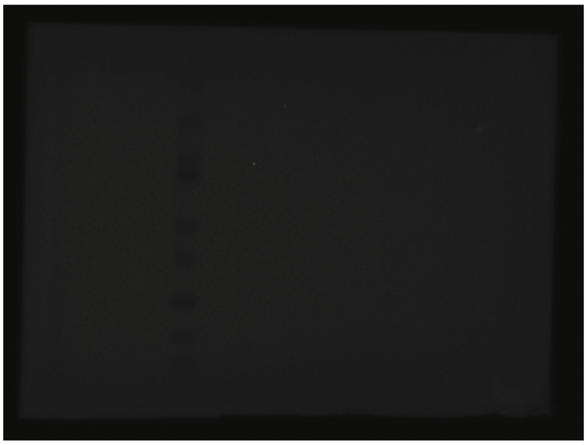
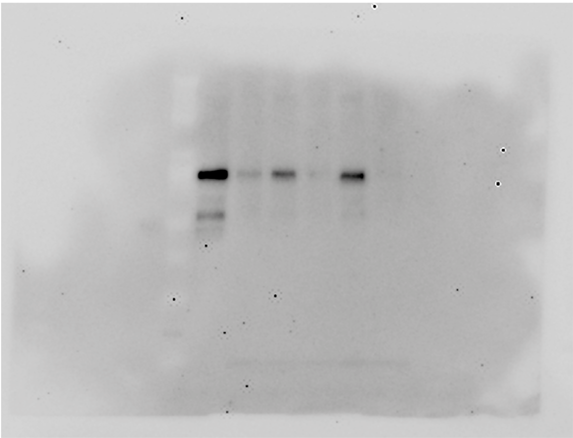

Replicate 2

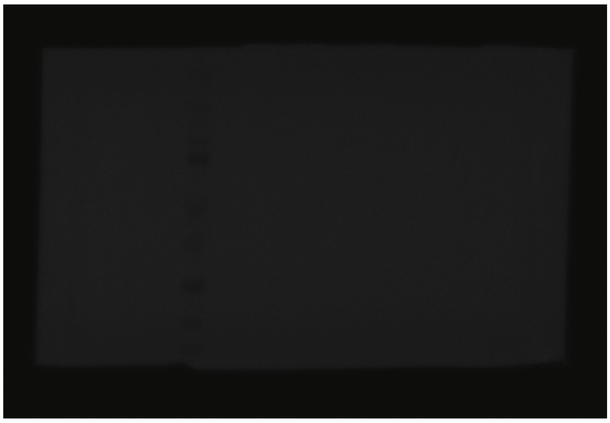
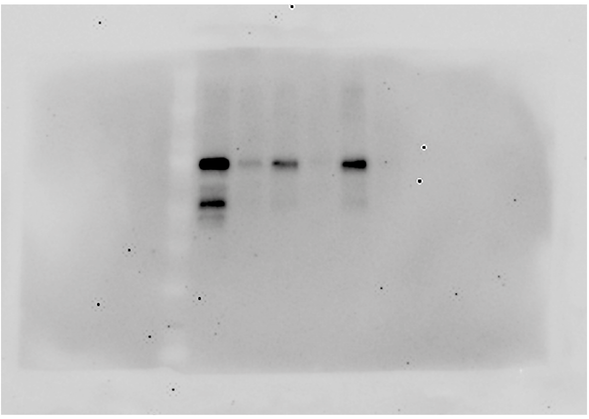

Replicate 3

Raw data Sup. Figure 4D

Myc

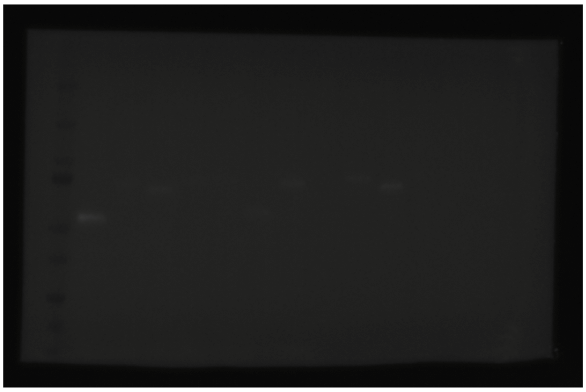

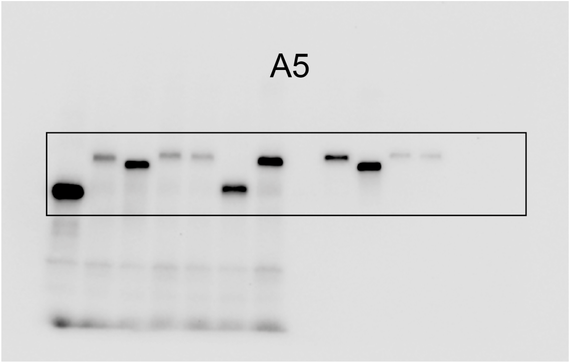

Replicate 1

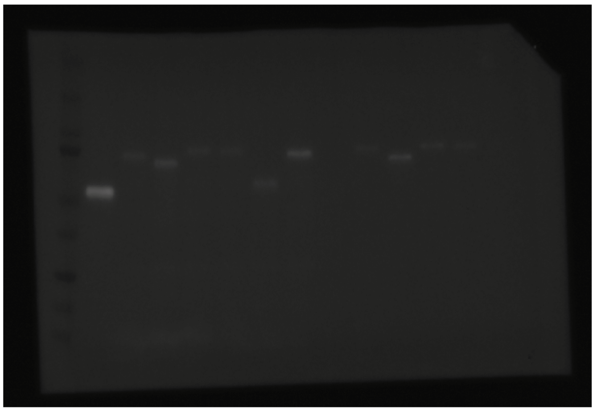

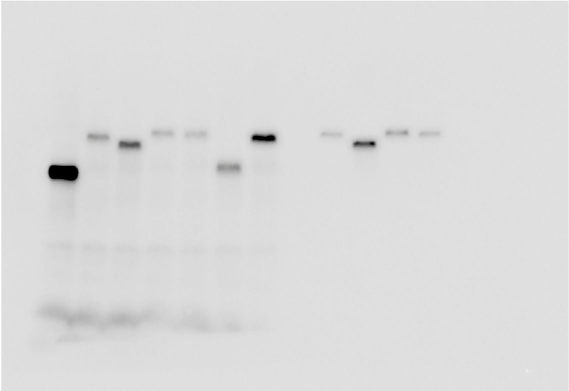

Replicate 2

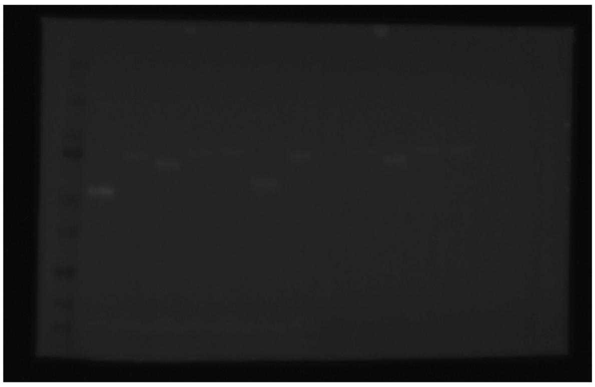

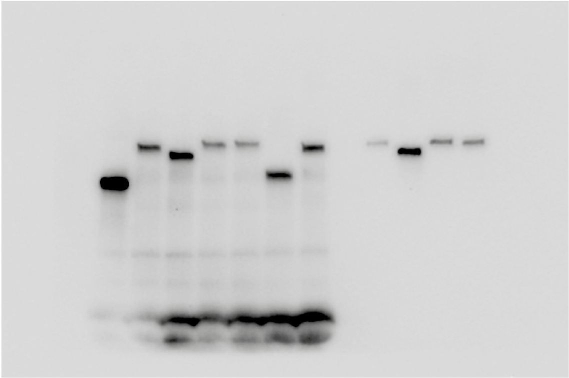

Replicate 3

Raw data Sup. Figure 4D

Myc

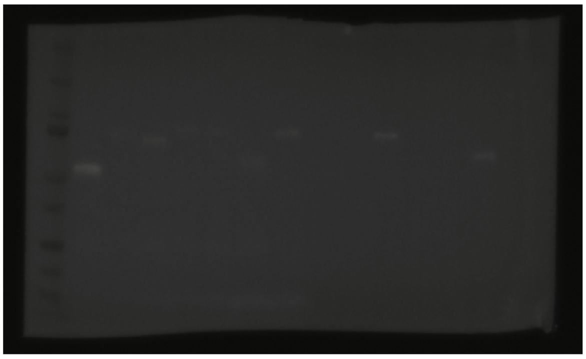

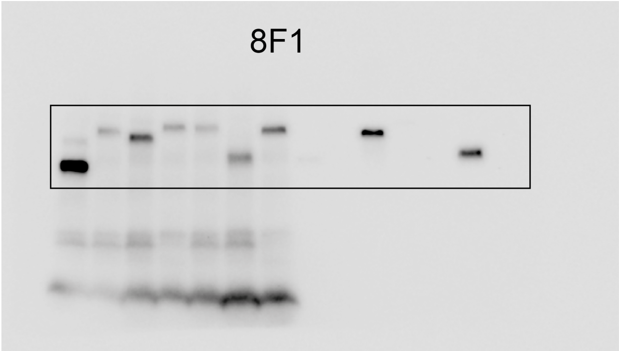

Replicate 1

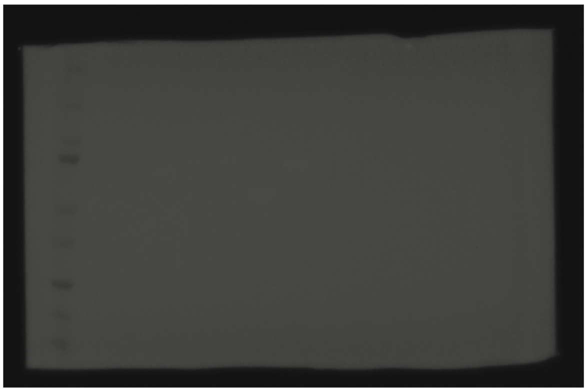

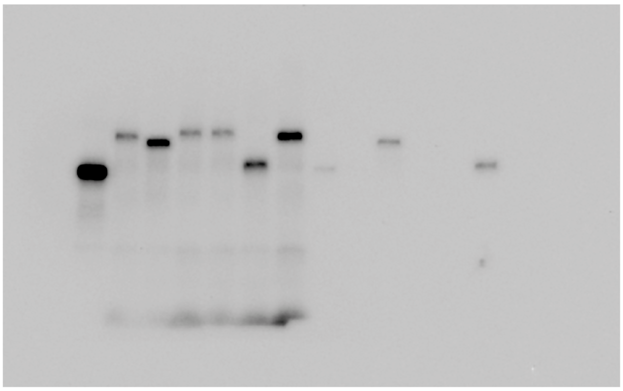

Replicate 2

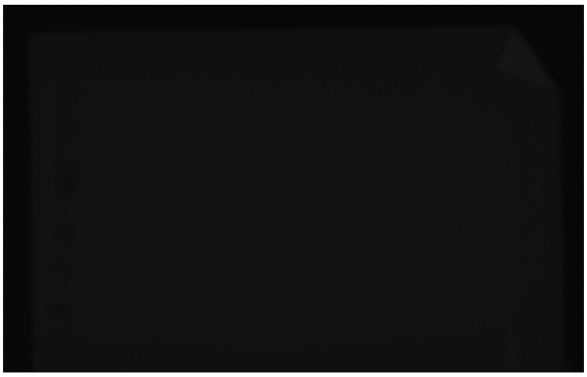

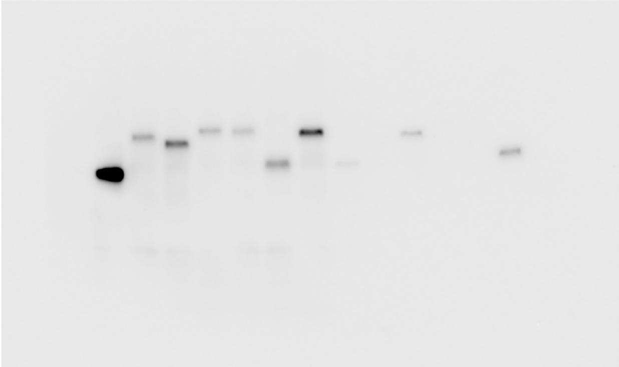

Replicate 3

Raw data Sup. Figure 4D

Myc

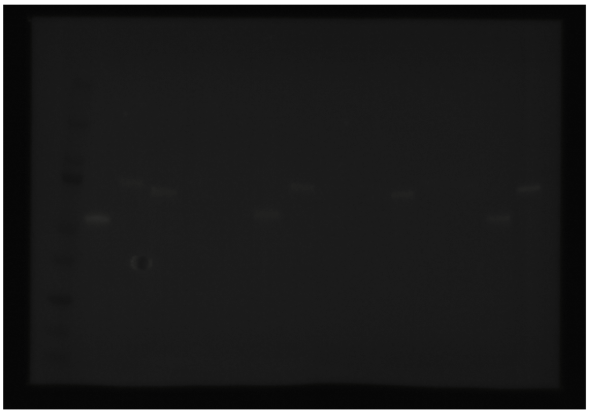

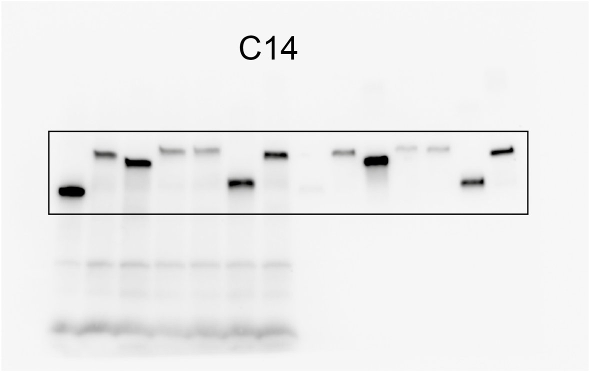

Replicate 1

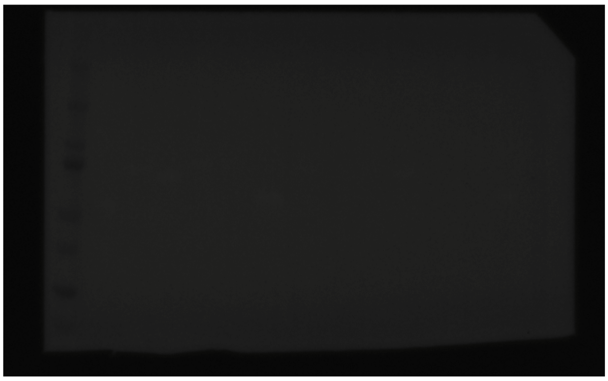

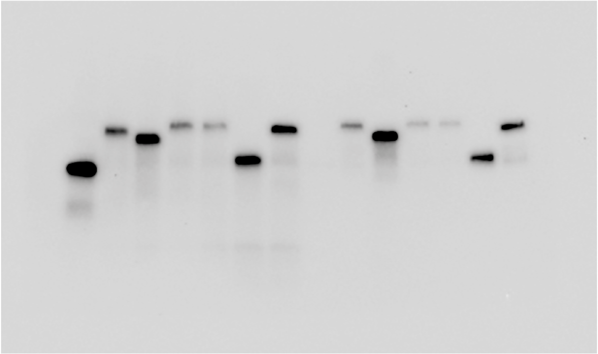

Replicate 2

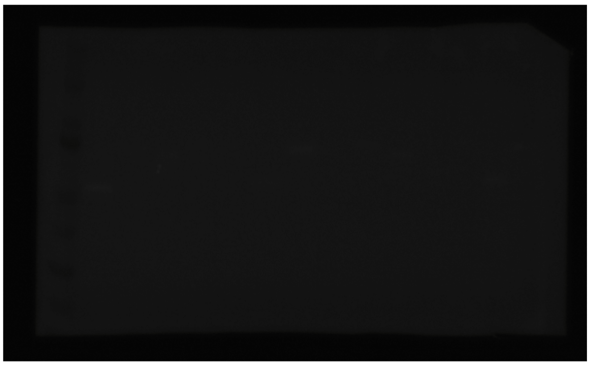

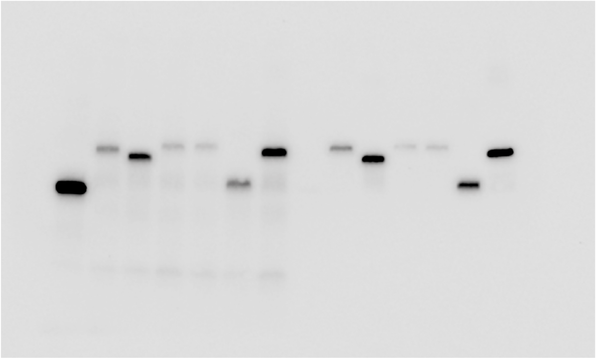

Replicate 3

Raw data Sup. Figure 4D

Myc

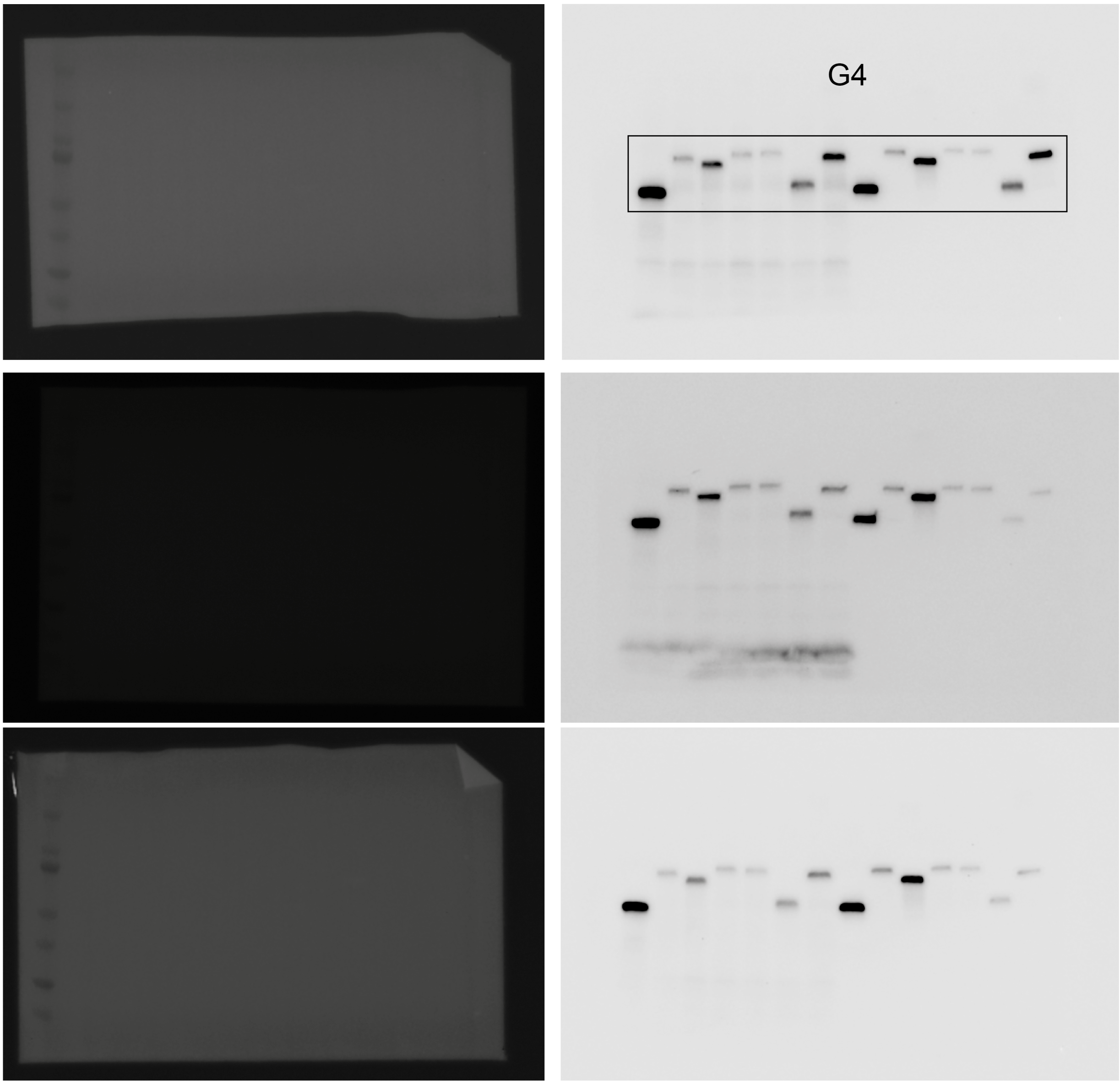

Replicate 1

Replicate 2

Replicate 3

Raw data Sup. Figure 4D

Myc

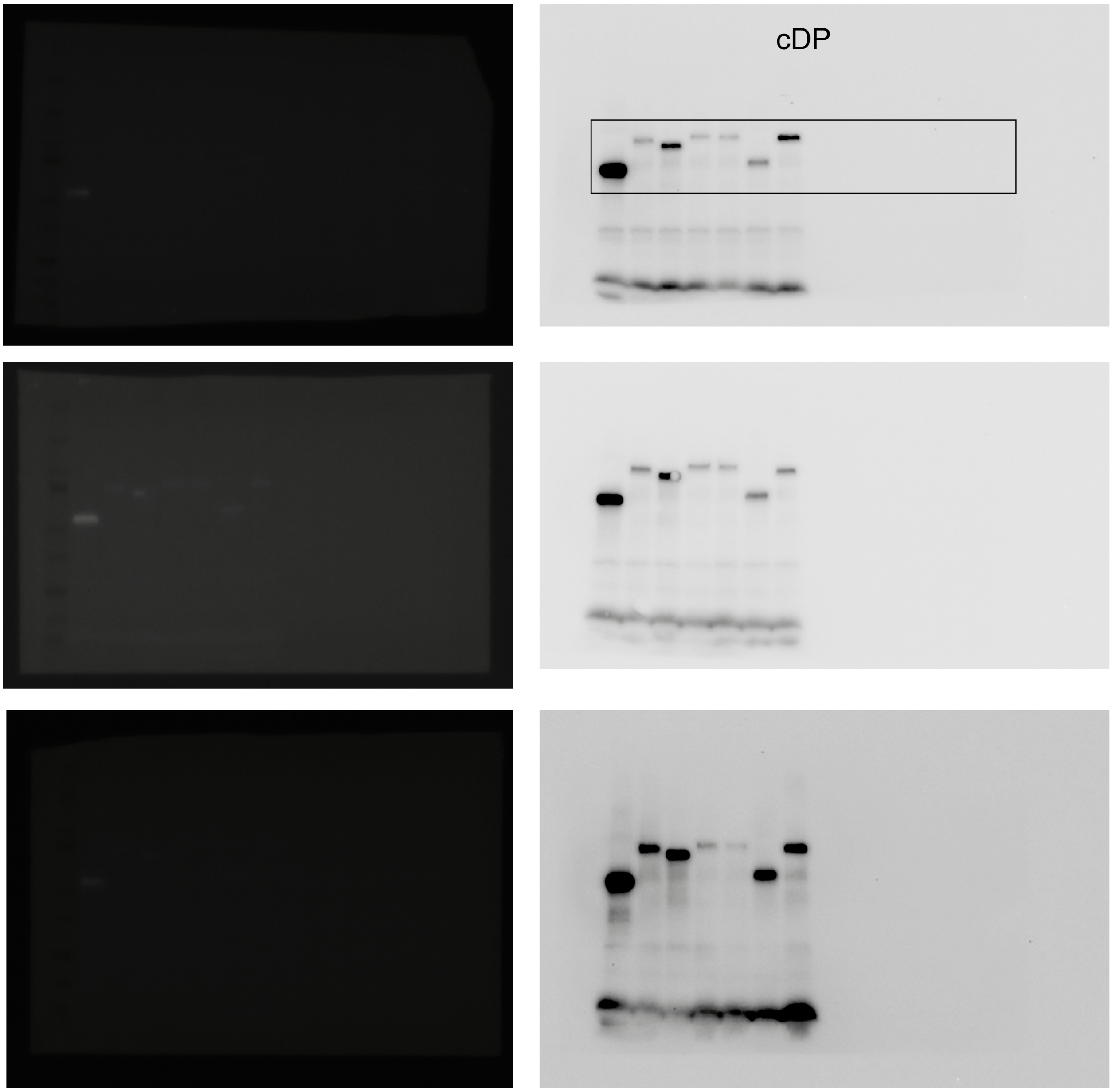

Replicate 1

Replicate 2

Replicate 3

Raw data Sup. Figure 6A,B p53 TAp73α

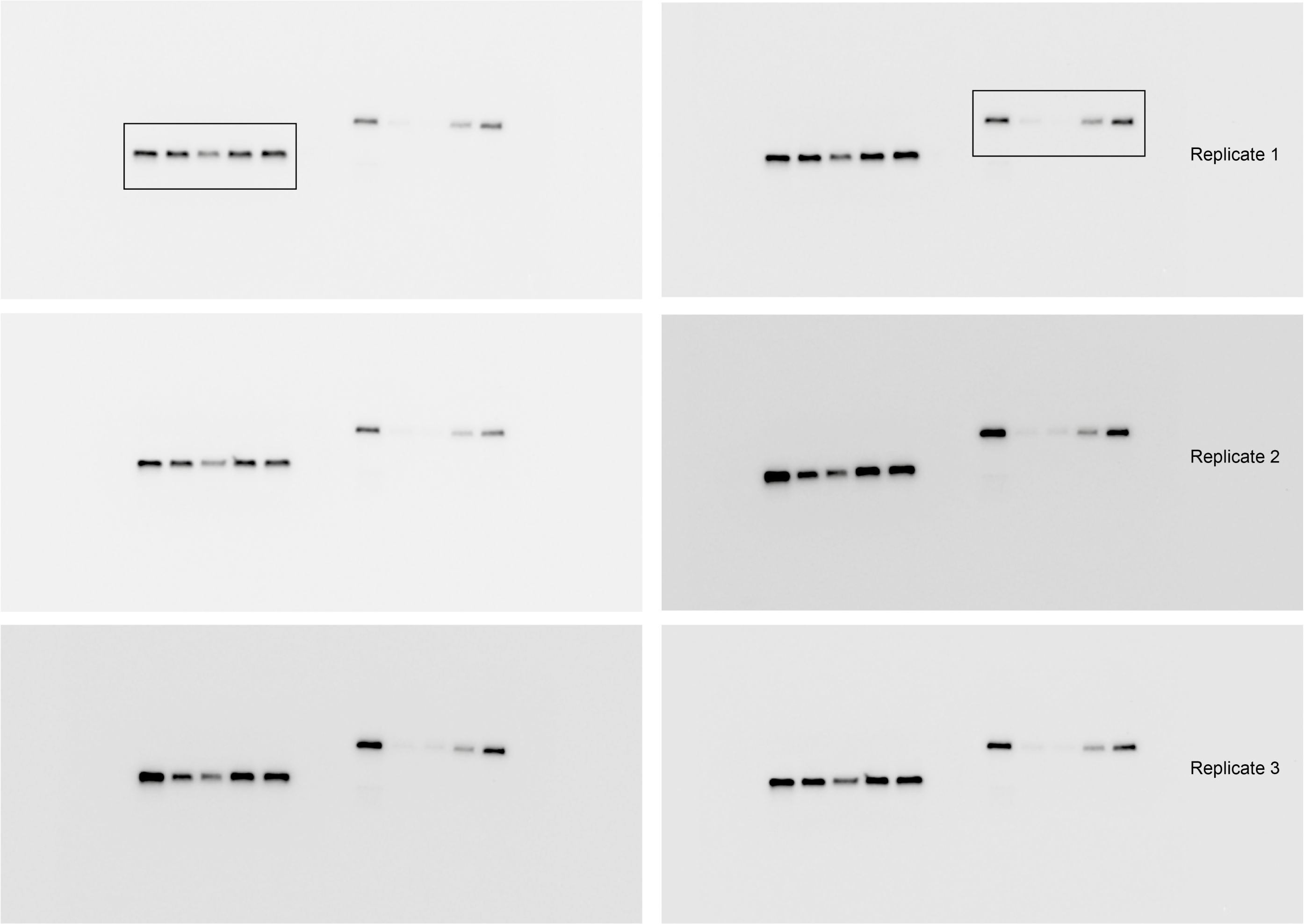

Top Replicate 1

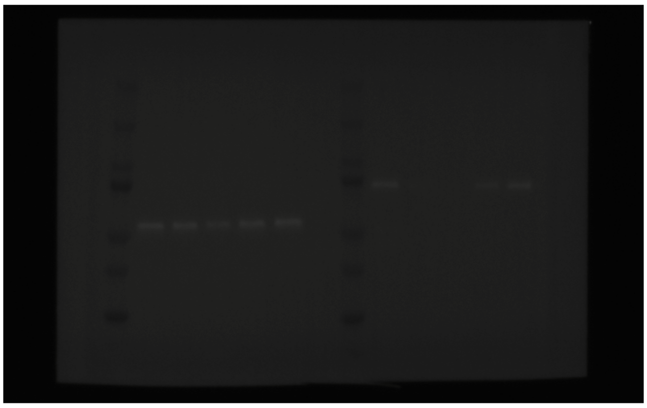

Top Replicate 2

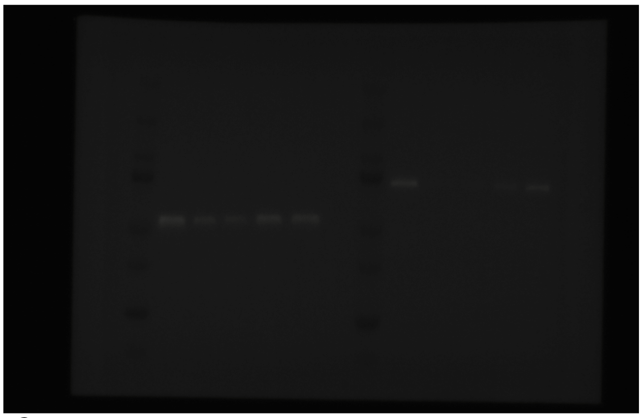

Top Replicate 3

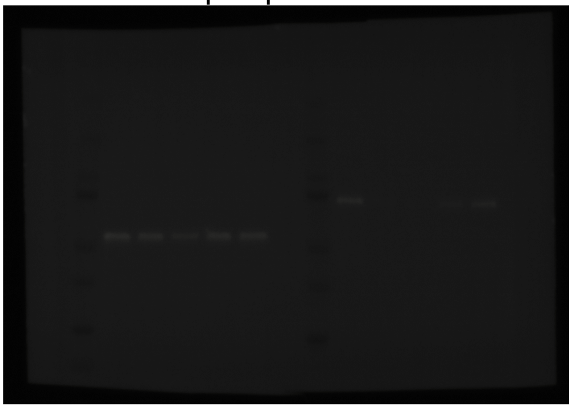

Supplement: Supplementary file 8 — Supplementary Figure7 [file 41418_2022_1030_MOESM8_ESM.pdf]
