# Supplementary material for: Designed Ankyrin Repeat Proteins as a tool box for analyzing p63
Source: Cell Death Differ. 2022 Jun 18;29(12):2445–58. doi: 10.1038/s41418-022-01030-y (PMC9751120; doi:10.1038/s41418-022-01030-y)
Supplement: Supplementary file 11 — Supplementary Table3 [file 41418_2022_1030_MOESM11_ESM.docx]

| **Complex** | DARPin C14‑p63 DBD | DARPin A5‑p63 SAM | DARPin 8F1‑p63 TD | DARPin  G4-p63 DBD |
| --- | --- | --- | --- | --- |
| **PDB accession code** | **7Z71** | **7Z72** | **7Z73** | **7Z7E** |
| ***Data Collection*** |  |  |  |  |
| Resolution^a^ (Å) | 44.64-1.85  (1.91-1.85) | 45.92-1.80  (1.86-1.80) | 47.06-2.27  (2.35-2.27) | 48.29-1.80  (1.86-1.80) |
| Spacegroup | *P* 1 | *P* 2_1_ | *P* 1 | P 3_1_ 2 1 |
| Cell dimensions | *a* = 53.0,  *b* = 63.9,  *c* = 65.5 Å | *a* = 48.5,  *b* = 43.9,  *c* = 50.0 Å | *a* = 42.6,  *b* = 50.8,  *c* = 65.9 Å | *A* = 96.6,  *b* = 96.6,  *c* = 77.1 Å |
|  | *α* = 114.5°*,*  *β* = 94.6°*,*  *γ* = 104.1° | *α, γ* = 90.0°,  *β* = 113.3° | *α* = 80.9°,  *β* = 79.9°,  *γ* = 68.8° | *α,β* = 90.0°,  *γ* = 120.0° |
| No. unique reflections^a^ | 58,153 (5,768) | 17,870 (1,745) | 21,523 (2,091) | 37,301 (2,211) |
| Completeness^a^ (%) | 91.6 (92.6) | 99.0 (99.6) | 92.6 (91.5) | 96.4 (96.6) |
| I/σI^a^ | 22.0 (9.5) | 11.8 (2.0) | 11.3 (2.2) | 23.0 (1.9) |
| R_merge_^a^ | 0.034 (0.110) | 0.090(0.802) | 0.055 (0.442) | 0.019 (0.32) |
| CC (1/2) | 0.998 (0.975) | 0.998 (0.688) | 0.997 (0.900) | 0.99 (0.826) |
| Redundancy^a^ | 3.7 (3.8) | 5.9 (6.0) | 3.8 (3.9) | 4.25 (1.07) |
| ***Refinement*** |  |  |  |  |
| No. atoms in refinement (P/O)^b^ | 5,390/ 690 | 1,733/ 167 | 3,514/ 51 | 2,676/ 183 |
| B factor (P/L/O)^b^ (Å^2^) | 26/ 35 | 25/ 36 | 63/ 55 | 44/ 32/ 43 |
| R_fact_ (%) | 14.2 | 15.9 | 18.7 | 19.8 |
| R_free_ (%) | 18.1 | 18.8 | 23.5 | 24.7 |
| rms deviation bond length^c^ (Å) | 0.016 | 0.014 | 0.011 | 0.010 |
| rms deviation angle^c^ (°) | 1.5 | 1.4 | 1.2 | 1.6 |
| Crystallization condition | 25% PEG 3350, 0.2 M sodium chloride, 0.1 M HEPES pH 7.5 | 25% PEG3350, 0.1 M citrate pH 3.5 | 25% PEG3350, 0.2 M sodium chloride, 0.1 M bis-tris pH 5.5 | 25% PEG 3350, 0.2 M lithium sulfate, 0.1 M HEPES pH 7.5 |

^a^ Values in brackets show the statistics for the highest resolution shells.

^b^ P/O indicate protein and others (water and solvent molecules), respectively.

^c^ rms indicates root-mean-square
