# Supplementary material for: Designed Ankyrin Repeat Proteins as a tool box for analyzing p63
Source: Cell Death Differ. 2022 Jun 18;29(12):2445–58. doi: 10.1038/s41418-022-01030-y (PMC9751120; doi:10.1038/s41418-022-01030-y)
Supplement: Supplementary file 16 — PDB validation report 4 [file 41418_2022_1030_MOESM16_ESM.pdf]

Full wwPDB X-ray Structure Validation Report (i)

Mar 23, 2022 – 06:25 pm GMT

PDB ID	:	7Z7E
Title	:	Crystal structure of p63 DNA binding domain in complex with inhibitory DARPin G4
Deposited on	:	2022-03-15
Resolution	:	1.80 Å(reported)

This wwPDB validation report is for manuscript review

This is a Full wwPDB X-ray Structure Validation Report.

This report is produced by the wwPDB biocuration pipeline after annotation of the structure.

We welcome your comments at *validation@mail.wwpdb.org*
A user guide is available at
https://www.wwpdb.org/validation/2017/XrayValidationReportHelp
with specific help available everywhere you see the (i) symbol.

The following versions of software and data (see references (i)) were used in the production of this report:

MolProbity	:	4.02b-467
Xtriage (Phenix)	:	1.13
EDS	:	2.27
buster-report	:	1.1.7 (2018)
Percentile statistics	:	20191225.v01 (using entries in the PDB archive December 25th 2019)
Refmac	:	5.8.0267
CCP4	:	7.1.010 (Gargrove)
Ideal geometry (proteins)	:	Engh & Huber (2001)
Ideal geometry (DNA, RNA)	:	Parkinson et al. (1996)
Validation Pipeline (wwPDB-VP)	:	2.27

For Manuscript Review

1 Overall quality at a glance (i)

The following experimental techniques were used to determine the structure:
X-RAY DIFFRACTION

The reported resolution of this entry is 1.80 Å.

Percentile scores (ranging between 0-100) for global validation metrics of the entry are shown in the following graphic. The table shows the number of entries on which the scores are based.

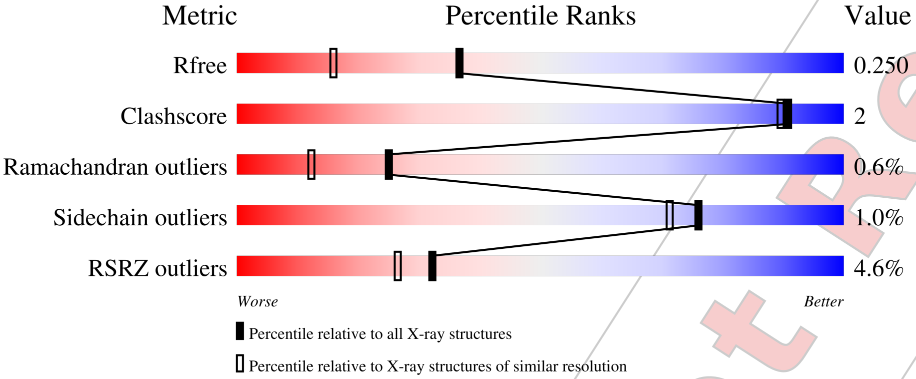

Metric	Whole archive (#Entries)	Similar resolution (#Entries, resolution range(Å))
R_{free}	130704	5950 (1.80-1.80)
Clashscore	141614	6793 (1.80-1.80)
Ramachandran outliers	138981	6697 (1.80-1.80)
Sidechain outliers	138945	6696 (1.80-1.80)
RSRZ outliers	127900	5850 (1.80-1.80)

The table below summarises the geometric issues observed across the polymeric chains and their fit to the electron density. The red, orange, yellow and green segments of the lower bar indicate the fraction of residues that contain outliers for >=3, 2, 1 and 0 types of geometric quality criteria respectively. A grey segment represents the fraction of residues that are not modelled. The numeric value for each fraction is indicated below the corresponding segment, with a dot representing fractions <=5% The upper red bar (where present) indicates the fraction of residues that have poor fit to the electron density. The numeric value is given above the bar.

Mol	Chain	Length	Quality of chain
1	A	204	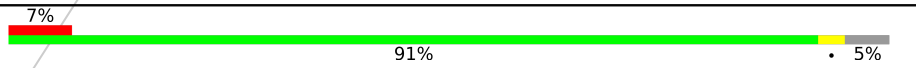
2	B	159	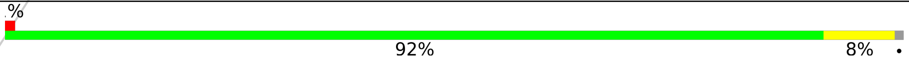

For Manuscript Review

2 Entry composition (i)

There are 4 unique types of molecules in this entry. The entry contains 5519 atoms, of which 2653 are hydrogens and 0 are deuteriums.

In the tables below, the ZeroOcc column contains the number of atoms modelled with zero occupancy, the AltConf column contains the number of residues with at least one atom in alternate conformation and the Trace column contains the number of residues modelled with at most 2 atoms.

- Molecule 1 is a protein called Isoform 4 of Tumor protein 63.

Mol	Chain	Residues	Atoms						ZeroOcc	AltConf	Trace
			Total	C	H	N	O	S			
1	A	193	2997	943	1495	267	280	12	53	0	0

There are 2 discrepancies between the modelled and reference sequences:

Chain	Residue	Modelled	Actual	Comment	Reference
A	122	GLY	-	expression tag	UNP Q9H3D4
A	123	SER	-	expression tag	UNP Q9H3D4

- Molecule 2 is a protein called DARPIN.

Mol	Chain	Residues	Atoms						ZeroOcc	AltConf	Trace
			Total	C	H	N	O	S			
2	B	158	2335	740	1158	203	231	3	35	0	0

- Molecule 3 is ZINC ION (three-letter code: ZN) (formula: Zn) (labeled as "Ligand of Interest" by depositor).

Mol	Chain	Residues	Atoms		ZeroOcc	AltConf
			Total	Zn		
3	A	1	1	1	0	0

- Molecule 4 is water.

Mol	Chain	Residues	Atoms		ZeroOcc	AltConf
			Total	O		
4	A	104	104	104	0	0
4	B	82	82	82	0	0

For Manuscript Review

3 Residue-property plots (i)

These plots are drawn for all protein, RNA, DNA and oligosaccharide chains in the entry. The first graphic for a chain summarises the proportions of the various outlier classes displayed in the second graphic. The second graphic shows the sequence view annotated by issues in geometry and electron density. Residues are color-coded according to the number of geometric quality criteria for which they contain at least one outlier: green = 0, yellow = 1, orange = 2 and red = 3 or more. A red dot above a residue indicates a poor fit to the electron density (RSRZ > 2). Stretches of 2 or more consecutive residues without any outlier are shown as a green connector. Residues present in the sample, but not in the model, are shown in grey.

- Molecule 1: Isoform 4 of Tumor protein 63

Chain A:

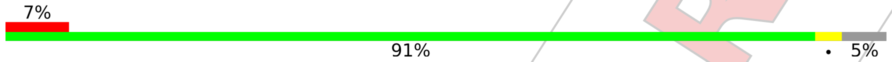

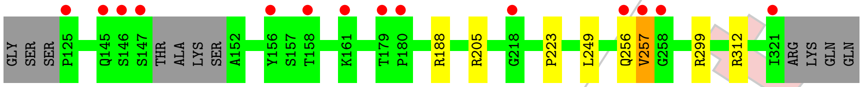

- Molecule 2: DARPIN

Chain B:

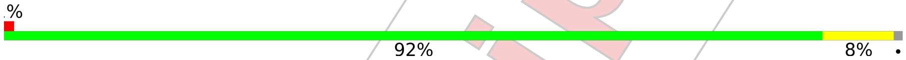

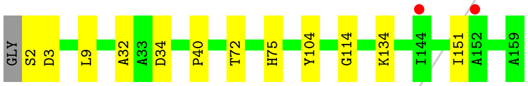

For Manuscript Review

4 Data and refinement statistics (i)

Property	Value	Source
Space group	P 31 2 1	Depositor
Cell constants<br>a, b, c, α, β, γ	96.58Å 96.58Å 77.07Å<br>90.00° 90.00° 120.00°	Depositor
Resolution (Å)	48.29 – 1.80<br>48.29 – 1.80	Depositor<br>EDS
% Data completeness<br>(in resolution range)	96.0 (48.29-1.80)<br>95.9 (48.29-1.80)	Depositor<br>EDS
R_{merge}	0.01	Depositor
R_{sym}	(Not available)	Depositor
$< I/\sigma(I) >$ [1]	1.34 (at 1.79Å)	Xtriage
Refinement program	REFMAC 5.8.0267	Depositor
R, R_{free}	0.197 , 0.249<br>0.204 , 0.250	Depositor<br>DCC
R_{free} test set	1862 reflections (5.00%)	wwPDB-VP
Wilson B-factor (Å^2)	37.3	Xtriage
Anisotropy	0.232	Xtriage
Bulk solvent k_{sol}(e/Å^3), B_{sol}(Å^2)	(Not available) , (Not available)	EDS
L-test for twinning[2]	$< \|L\| > = 0.50$, $< L^2 > = 0.33$	Xtriage
Estimated twinning fraction	0.020 for -h,-k,l	Xtriage
F_o,F_c correlation	0.96	EDS
Total number of atoms	5519	wwPDB-VP
Average B, all atoms (Å^2)	43.0	wwPDB-VP

Xtriage's analysis on translational NCS is as follows: *The largest off-origin peak in the Patterson function is 4.72% of the height of the origin peak. No significant pseudotranslation is detected.*

[1] Intensities estimated from amplitudes.

[2] Theoretical values of $< |L| >$, $< L^2 >$ for acentric reflections are 0.5, 0.333 respectively for untwinned datasets, and 0.375, 0.2 for perfectly twinned datasets.

For Manuscript Review

5 Model quality (i)

5.1 Standard geometry (i)

Bond lengths and bond angles in the following residue types are not validated in this section: ZN

The Z score for a bond length (or angle) is the number of standard deviations the observed value is removed from the expected value. A bond length (or angle) with $|Z| > 5$ is considered an outlier worth inspection. RMSZ is the root-mean-square of all Z scores of the bond lengths (or angles).

Mol	Chain	Bond lengths		Bond angles	
		RMSZ	#|Z| >5	RMSZ	#|Z| >5
1	A	0.73	0/1538	0.89	1/2091 (0.0%)
2	B	0.76	0/1198	0.82	0/1630
All	All	0.75	0/2736	0.86	1/3721 (0.0%)

There are no bond length outliers.

All (1) bond angle outliers are listed below:

Mol	Chain	Res	Type	Atoms	Z	Observed(^{o})	Ideal(^{o})
1	A	299	ARG	NE-CZ-NH1	5.30	122.95	120.30

There are no chirality outliers.

There are no planarity outliers.

5.2 Too-close contacts (i)

In the following table, the Non-H and H(model) columns list the number of non-hydrogen atoms and hydrogen atoms in the chain respectively. The H(added) column lists the number of hydrogen atoms added and optimized by MolProbity. The Clashes column lists the number of clashes within the asymmetric unit, whereas Symm-Clashes lists symmetry-related clashes.

Mol	Chain	Non-H	H(model)	H(added)	Clashes	Symm-Clashes
1	A	1502	1495	1481	3	0
2	B	1177	1158	1143	6	0
3	A	1	0	0	0	0
4	A	104	0	0	0	0
4	B	82	0	0	0	0
All	All	2866	2653	2624	9	0

The all-atom clashscore is defined as the number of clashes found per 1000 atoms (including

For Manuscript Review

hydrogen atoms). The all-atom clashscore for this structure is 2.

All (9) close contacts within the same asymmetric unit are listed below, sorted by their clash magnitude.

Atom-1	Atom-2	Interatomic distance (Å)	Clash overlap (Å)
2:B:72:THR:H	2:B:75:HIS:HD2	1.33	0.74
1:A:188:ARG:HB2	1:A:249:LEU:HD23	1.85	0.58
2:B:32:ALA:O	2:B:40:PRO:HD3	2.13	0.48
1:A:205:ARG:HD3	1:A:223:PRO:O	2.14	0.46
2:B:72:THR:H	2:B:75:HIS:CD2	2.22	0.46
1:A:256:GLN:O	1:A:257:VAL:HG22	2.18	0.43
2:B:114:GLY:HA2	2:B:151:ILE:HD12	2.01	0.42
2:B:9:LEU:HD13	2:B:34:ASP:HB3	2.03	0.41
2:B:75:HIS:HE1	2:B:104:TYR:O	2.05	0.40

There are no symmetry-related clashes.

5.3 Torsion angles (i)

5.3.1 Protein backbone (i)

In the following table, the Percentiles column shows the percent Ramachandran outliers of the chain as a percentile score with respect to all X-ray entries followed by that with respect to entries of similar resolution.

The Analysed column shows the number of residues for which the backbone conformation was analysed, and the total number of residues.

Mol	Chain	Analysed	Favoured	Allowed	Outliers	Percentiles	
1	A	189/204 (93%)	183 (97%)	5 (3%)	1 (0%)	29	15
2	B	156/159 (98%)	154 (99%)	1 (1%)	1 (1%)	25	12
All	All	345/363 (95%)	337 (98%)	6 (2%)	2 (1%)	25	12

All (2) Ramachandran outliers are listed below:

Mol	Chain	Res	Type
1	A	257	VAL
2	B	3	ASP

For Manuscript Review

5.3.2 Protein sidechains (i)

In the following table, the Percentiles column shows the percent sidechain outliers of the chain as a percentile score with respect to all X-ray entries followed by that with respect to entries of similar resolution.

The Analysed column shows the number of residues for which the sidechain conformation was analysed, and the total number of residues.

Mol	Chain	Analysed	Rotameric	Outliers	Percentiles	
1	A	169/180 (94%)	168 (99%)	1 (1%)	86	84
2	B	118/120 (98%)	116 (98%)	2 (2%)	60	51
All	All	287/300 (96%)	284 (99%)	3 (1%)	76	71

All (3) residues with a non-rotameric sidechain are listed below:

Mol	Chain	Res	Type
1	A	312	ARG
2	B	2	SER
2	B	134	LYS

Sometimes sidechains can be flipped to improve hydrogen bonding and reduce clashes. All (2) such sidechains are listed below:

Mol	Chain	Res	Type
1	A	198	HIS
2	B	75	HIS

5.3.3 RNA (i)

There are no RNA molecules in this entry.

5.4 Non-standard residues in protein, DNA, RNA chains (i)

There are no non-standard protein/DNA/RNA residues in this entry.

5.5 Carbohydrates (i)

There are no monosaccharides in this entry.

For Manuscript Review

5.6 Ligand geometry (i)

Of 1 ligands modelled in this entry, 1 is monoatomic - leaving 0 for Mogul analysis.

There are no bond length outliers.

There are no bond angle outliers.

There are no chirality outliers.

There are no torsion outliers.

There are no ring outliers.

No monomer is involved in short contacts.

5.7 Other polymers (i)

There are no such residues in this entry.

5.8 Polymer linkage issues (i)

There are no chain breaks in this entry.

For Manuscript Review

6 Fit of model and data (i)

6.1 Protein, DNA and RNA chains (i)

In the following table, the column labelled '#RSRZ> 2' contains the number (and percentage) of RSRZ outliers, followed by percent RSRZ outliers for the chain as percentile scores relative to all X-ray entries and entries of similar resolution. The OWAB column contains the minimum, median, 95th percentile and maximum values of the occupancy-weighted average B-factor per residue. The column labelled 'Q< 0.9' lists the number of (and percentage) of residues with an average occupancy less than 0.9.

Mol	Chain	Analysed	<RSRZ>	#RSRZ>2			OWAB(Å^2)	Q<0.9
1	A	193/204 (94%)	0.34	14 (7%)	15	11	28, 41, 67, 107	0
2	B	158/159 (99%)	0.26	2 (1%)	77	74	29, 40, 66, 83	0
All	All	351/363 (96%)	0.30	16 (4%)	32	26	28, 41, 67, 107	0

All (16) RSRZ outliers are listed below:

Mol	Chain	Res	Type	RSRZ
1	A	257	VAL	4.8
1	A	125	PRO	4.8
1	A	158	THR	4.1
1	A	256	GLN	4.0
1	A	145	GLN	3.6
1	A	258	GLY	3.3
1	A	146	SER	3.0
1	A	147	SER	2.9
2	B	152	ALA	2.9
1	A	321	ILE	2.8
1	A	180	PRO	2.7
1	A	156	TYR	2.6
2	B	144	ILE	2.4
1	A	161	LYS	2.4
1	A	218	GLY	2.3
1	A	179	THR	2.2

6.2 Non-standard residues in protein, DNA, RNA chains (i)

There are no non-standard protein/DNA/RNA residues in this entry.

For Manuscript Review

6.3 Carbohydrates (i)

There are no monosaccharides in this entry.

6.4 Ligands (i)

In the following table, the Atoms column lists the number of modelled atoms in the group and the number defined in the chemical component dictionary. The B-factors column lists the minimum, median, 95th percentile and maximum values of B factors of atoms in the group. The column labelled 'Q< 0.9' lists the number of atoms with occupancy less than 0.9.

Mol	Type	Chain	Res	Atoms	RSCC	RSR	B-factors($Å^2$)	Q<0.9
3	ZN	A	401	1/1	0.99	0.14	32,32,32,32	0

The following is a graphical depiction of the model fit to experimental electron density of all instances of the Ligand of Interest. In addition, ligands with molecular weight > 250 and outliers as shown on the geometry validation Tables will also be included. Each fit is shown from different orientation to approximate a three-dimensional view.

For Manuscript Review

Electron density around ZN A 401:

$2mF_o$-DF_c (at 0.7 rmsd) in gray
mF_o-DF_c (at 3 rmsd) in purple (negative)
and green (positive)

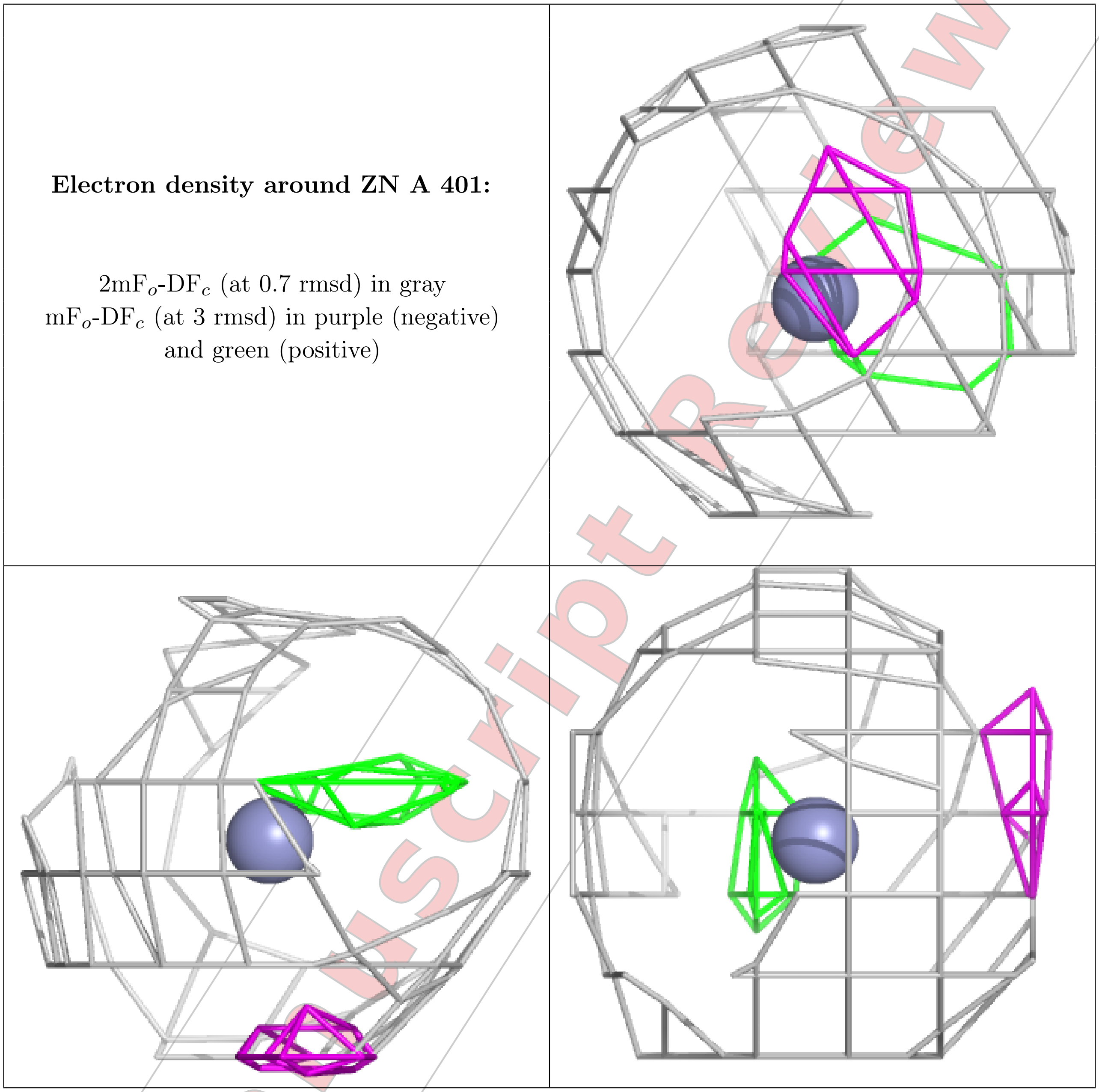

6.5 Other polymers (i)

There are no such residues in this entry.

For Manuscript Review
